# Donor and Geometry Optimization: Fresh Perspectives for the Design of Polyoxometalate Charge Transfer Chromophores

**DOI:** 10.1021/acs.inorgchem.5c00915

**Published:** 2025-04-14

**Authors:** Bethany R. Hood, Yovan de Coene, Claire F. Jones, Noah Deveaux, Jack M. Barber, Charlotte G. Marshall, Chloe A. Jordan, Nathan R. Halcovitch, Benoît Champagne, Koen Clays, John Fielden

**Affiliations:** † Department of Chemistry, Lancaster University, Lancaster LA1 4YW, U.K.; ‡ School of Chemistry, University of East Anglia, Norwich NR4 7TJ, U.K.; § School of Chemistry, Pharmacy and Pharmacology, University of East Anglia, Norwich NR4 7TJ, U.K.; ∥ Department of Chemistry, University of Leuven, Celestijnenlaan 200D, Leuven 3001, Belgium; ⊥ Unit of Theoretical and Structural Physical Chemistry, Namur Institute of Structured Matter, University of Namur, Namur B-5000, Belgium

## Abstract

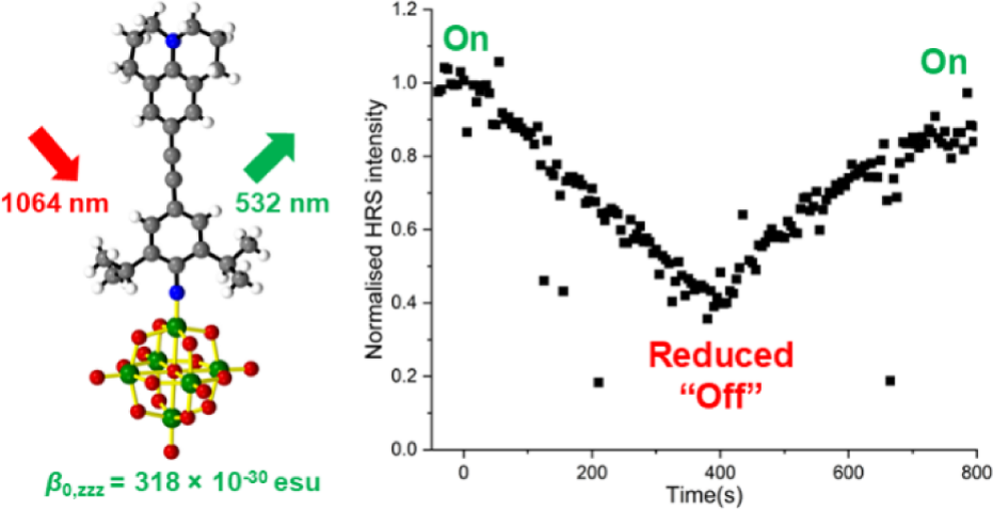

Three linear, dipolar arylimido-polyoxometalate (POM) and one 2-dimensional *bis*-functionalized arylimido-polyoxometalate charge transfer chromophore, with diphenylacetylene bridges, have been synthesized and studied by spectroelectrochemistry, hyper-Rayleigh scattering (HRS), and DFT/TD-DFT calculations. The linear systems show that with julolidinyl (Jd) and −NTol_2_ donor groups, the alkyne bridge yields high second-order nonlinear optical (NLO) coefficients β (Jd, β_0,*zzz*_ = 318 × 10^–30^ esu; −NTol_2_, β_0,*zzz*_ = 222 × 10^–30^ esu), indeed the Jd compound gives the highest NLO activity of any organoimido-POM to date with minimal decrease in transparency. The *bis*-functionalized 2D (*C*_2v_) POM derivative showed increased activity over its monofunctionalized analogue with no decrease in transparency, although the NLO response was only minimally two dimensional. Spectroelectrochemistry and TD-DFT calculations showed switchable linear optical responses for the monofunctionalized derivatives due to the weakened charge transfer character of the electronic transitions in the reduced state, while TD-DFT also indicated potential for switched NLO responses. These have been demonstrated by electrochemistry-HRS for the Jd compound, but cyclability is limited by relatively poor stability in the reduced state. IR and CV studies for these sterically protected arylimido polyoxometalates indicate that decomposition proceeds via a breakdown of the {Mo_6_} cluster in the reduced state, rather than simple solvolysis of the Mo≡N bond.

## Abstract 2

Use of a very strong alkylamino (julolidinyl) donor group and connection of a second donor unit to the arylimido-polyoxometalate acceptor are shown to be effective strategies to enhance second-order nonlinear optical (NLO) responses in hybrid polyoxometalate charge transfer chromophores. The julolidinyl derivative has the highest NLO activity yet reported for a polyoxometalate derivative (β_0,*zzz*_ = 318 × 10^−30^ esu), and its response can be attenuated by electrochemical reduction.

## Introduction

Polyoxometalates (POMs) are a vast family of anionic cluster compounds consisting primarily of transition metal ions (typically Mo and W) in their highest oxidation state and both bridging and terminal oxo ligands.^1^ Their extensive range of sizes—all the way from subnanometer {M_5_} and {M_6_} clusters to giant, multinanometer architectures such as {Mo_368_}—shapes,^2^ rich redox chemistry, and ability to incorporate heteroatoms lead to a wide range of potential uses in areas including catalysis,^3^ medicine,^4^ and data storage.^5^ Moreover, POMs can be derivatized with organic ligands,^6^ further expanding and modulating their range of properties and applications. Here and in previous work, our focus is on arylimido Lindqvist POMs because conjugation between aryl unit and POM across the imido bond enables stronger electronic communication between the organic donor/π-system and POM core than typically seen in other functionalized POMs based on more electronically isolating W-O-X-Ar linkages (where X = main group elements, e.g., Si, P, As, and Sn). This is reflected in strongly shifted redox potentials, new electronic transitions, and *emergent* properties such as nonlinear optical (NLO) activity^7^—most of all, second-order effects (e.g., laser frequency doubling). These are essential to many technologies that rely on manipulation of laser light, and the fast responses obtained from molecular chromophores (including “POMophore” systems) could enable potential uses in telecommunications and optical/electro-optical computing.^8^

Two key challenges for molecular NLO materials are addressing nonlinearity/transparency trade-offs,^9^ and making materials whose properties can be reversibly switched between two or more states.^10^ The former is important because modifications that increase activity tend to increase visible and NIR light absorption, potentially affecting efficiency and stability of any device. The latter is relevant to the development of molecular optical and nonlinear optical transistors, memory devices, and sensors. POMophores^7^ have shown promise in these areas, by enabling high NLO coefficients β to be obtained from short, stable π-bridges that minimize visible-light absorption, and in one example showing highly cyclable electrochemically switched NLO responses,^11^ without using metal donors (e.g., Ru^II^) which tend to introduce low-energy visible absorptions.

In this work, we explore developing POM-based NLO chromophores and redox switches in terms of activity, transparency, and ease of synthesis. Stabilizing the arylimido Mo≡N bond enough to allow reversible multicycle redox switching depends on use of bulky groups (i.e., ^*i*^Pr) in the 2,6 positions of the ring, and our previous work featured these on a single phenyl bridge linking the POM to an −NMe_2_ donor.^11^ Changes of donor (D) can give big increases in β and thus higher “on” state signal, but suitable ^*i*^Pr-derivatized phenylaniline precursors are not readily accessible. Therefore, we investigate diphenylacetylene-bridged POM chromophores for redox-mediated NLO switching for the first time, investigating the effect of −NMe_2_, −NTol_2_, and julolidinyl (Jd) donors that can be attached to iodo-arylimido-POM precursors through well-established Sonogashira methods, and also test the effect of introducing a second donor group in a 2-dimensional (*C*_2v_) D-A-D geometry (A = acceptor POM). The results reveal a new record POMophore response for the -Jd donor (β_0,*zzz*_ = 320 × 10^–30^ esu), showing for the first time that very strong alkylamino donors can exceed the performance of arylamino systems (−NTol_2_ and −NPh_2_), and indicate that D-A-D POMophores can increase response without sacrificing transparency. They also show the diphenylethyne-bridged systems can be redox switched but undergo a redox-induced decomposition of the POM core.

## Experimental Section

### Materials and Procedures

Anhydrous dimethyl sulfoxide (DMSO) was purchased from Sigma-Aldrich (SureSeal) and Acros Organics (AcroSeal) and used as supplied. All other reagents and solvents were obtained as ACS grade from Sigma-Aldrich, Alfa Aesar, Fisher Scientific, Fluorochem, Acros Organics, or Apollo Scientific and used as supplied. Deuterated solvents were obtained from Eurisotop, Cambridge Isotope Laboratories, or Acros Organics and used as supplied. Tetrabutylammonium hexamolybdate was synthesized according to previously reported methods.^12^ 4-Iodo-2,6-diisopropylaniline (**P1**), iodo-arylimido polyoxometalates [NBu_4_]_2_[Mo_6_O_18_NC_12_H_16_I] ([NBu_4_]_2_[**P2**]) and [NBu_4_]_2_[Mo_6_O_17_(NC_12_H_16_I)_2_] ([NBu_4_]_2_[**P3**]), and all other precursor compounds (**P4** to **P8**) were synthesized by using or adapting known methods, with full details and references given in the Supporting Information. Unless otherwise stated, reactions were performed under an atmosphere of dry argon using standard Schlenk techniques.

### General Physical Measurements

FT-IR spectra were measured using a Bruker FT-IR XSA spectrometer. ^1^H and ^13^C NMR spectra were acquired using a Bruker Ascend 500 (500 MHz) spectrometer and all shifts are quoted with respect to TMS using the solvent signals as a secondary standard (s = singlet, d = doublet, t = triplet, q = quartet, quin = quintet, sex = sextet, *a*sex = apparent sextet, hept = heptet, m = multiplet). Some quaternary carbon signals were not observed for these compounds, similar to previously synthesized arylimido-polyoxometalates.^7^ Elemental analyses and accurate mass spectrometry were outsourced to the University of Manchester and the John Innes Centre (Norwich), respectively. UV–vis spectra were obtained using an Agilent Cary 60 UV–vis spectrophotometer.

### Preparation of [NBu_4_]_2_[Mo_6_O_18_N_2_C_22_H_26_] ([NBu_4_]_2_[**1**])

Compound **P2** (0.617 g, 0.374 mmol), 4-ethynyl-*N*,*N*-dimethylaniline (0.0652 g, 0.449 mmol), bis(triphenylphosphine) palladium(II) dichloride (7.4 mg, 0.011 mmol), copper iodide (3.4 mg, 0.018 mmol), and potassium carbonate (0.375 g, 2.7 mmol) were dissolved in dry acetonitrile (15 mL) before dry triethylamine (0.75 mL, 5.375 mmol) was added. The resulting mixture was stirred at room temperature for 30 min before the filtrate was evaporated to 2 mL and poured into diethyl ether (50 mL) resulting in a dark-red precipitate. This was collected by filtration, washed with diethyl ether, and then dried in vacuo to give [NBu_4_]_2_[**1**] as a dark-red solid (0.332 g, 0.201 mmol, 54%). ^1^H NMR (500 MHz, CD_3_CN): δ 7.37 (d, *J* = 9.0 Hz, 2H), 7.25 (s, 2H), 6.73 (d, *J* = 9.0 Hz, 2H), 3.83 (hept, *J* = 6.9 Hz, 2H), 3.13–3.06 (m, 16H), 2.98 ppm (s, 6H), 1.66–1.56 (m, 16H), 1.36 (*a*sex, *J* = 7.5 Hz, 16H), 1.31 (d, *J* = 6.8 Hz, 12H), 0.97 (t, *J* = 7.4 Hz, 24H). ^13^C NMR (400 MHz, CD_3_CN): δ 151.64, 149.83, 133.62, 125.71, 124.59, 112.85, 109.87, 94.12, 88.41, 68.25, 59.30, 40.32, 29.43, 24.32, 23.97, 20.31, 13.79. Anal. calcd % for C_54_H_98_N_4_O_18_Mo_6_ ([NBu_4_]_2_[**1**]): C, 38.90 (38.90); H, 5.93 (5.89); N, 3.36 (3.35). HRMS (ESI, MeCN) *m*/*z*: 592.7750, calcd for C_22_H_26_N_2_Mo_6_O_18_^2–^ ([**1**]^2–^), 591.7767. FTIR (ATR) cm^–1^: 2960 (m), 2932 (sh), 2872 (m), 2187 (m), 1606 (m), 1585 (m), 1585 (s), 1521 (m), 1445 (m), 1350 (w), 1227 (w), 1191 (m), 1169 (m), 1150 (m), 1063 (w), 974 (s), 943 (m), 882 (w), 855 (sh), 769 (w), 740 (sh). UV–vis (MeCN) λ, nm (ε, M^–1^ cm^–1^): 203 (73.6 × 10^3^), 293 (37.1 × 10^3^), 424 (37.6 × 10^3^).

### Preparation of [NBu_4_]_2_[Mo_6_O_18_N_2_C_34_H_34_] ([NBu_4_]_2_[**2**])

Compound **P2** (1.433 g, 0.941 mmol), 4-ethylyphenyl-4,4′-ditolylamine (**P6**) (0.4163 g, 1.428 mmol), (PPh_3_)_2_PdCl_2_ (12.6 mg, 0.018 mmol), copper iodide (8 mg, 0.042 mmol), and potassium carbonate (0.898 g, 6.50 mmol) were dissolved in dry acetonitrile (35 mL) before dry triethylamine (1.8 mL, 12.9 mmol) was added. The mixture was stirred for 80 min at room temperature before the filtrate was evaporated to 3 mL and poured into diethyl ether (200 mL). The resulting dark red precipitate was collected by filtration, washed with ethanol and diethyl ether, and then dried in vacuo to give compound [NBu_4_]_2_[**2**] as a dark-red solid (0.795 g, 0.438 mmol, 47%). ^1^H NMR (500 MHz, CD_3_CN): δ 7.33 (d, *J* = 8.8 Hz, 2H), 7.28 (s, 2H), 7.16 (d, *J* = 8.1 Hz, 4H), 7.00 (d, *J* = 8.4 Hz, 4H), 6.86 (d, *J* = 8.8 Hz, 2H), 3.83 (hept, *J* = 6.7 Hz, 2H), 3.15–3.06 (m, 16H), 2.31 (s, 6H), 1.66–1.56 (m, 16H), 1.38 (*a*sex, *J* = 8.0 Hz, 16H), 1.31 (d, *J* = 6.8 Hz, 12H), 0.97 (t, *J* = 7.4 Hz, 24H). ^13^C NMR (126 MHz, CD_3_CN): δ 149.80, 145.35, 134.99, 133.43, 131.11, 126.50, 126.00, 121.05, 59.28, 29.43, 24.31, 23.94, 20.84, 20.30, 13.78. Anal. calcd % for C_66_H_106_N_4_O_18_Mo_6_ ([NBu_4_]_2_[**2**]): C, 43.57 (43.89); H, 5.87 (6.03); N, 3.08 (3.02). HRMS (ESI, MeCN) *m*/*z*: 666.8062, calcd for C_34_H_34_N_2_Mo_6_O_18_^2–^ ([**2**]^2–^), 666.8079. FTIR (ATR) cm^–1^: 2959 (s), 2932 (m), 2872 (m), 2189 (w), 1600 (sh), 1585 (s), 1503 (vs), 1480 (s), 1380 (m), 1320 (s), 1294 (m), 1277 (m), 1151 (w), 1107 (w), 1067 (w), 1031 (w), 974 (s), 944 (vs), 880 (s), 774 (vs). UV–vis (MeCN) λ, nm (ε, M^–1^ cm^–1^): 206 (90.4 × 10^3^), 294 (33.1 × 10^3^), 329 (19.0 × 10^3^), 423 (39.4 × 10^3^).

### Preparation of [NBu_4_]_2_[Mo_6_O_18_N_2_C_26_H_30_] ([NBu_4_]_2_[**3**])

To a mixture of compound **P3** (0.618 g, 0.388 mmol), 4-ethynyljuloloidine (**P8**) (0.106 g, 0.537 mmol), bis(triphenylphosphine)palladium(II) chloride (0.009 g, 0.013 mmol), copper iodide (0.003 g, 0.027 mmol), and potassium carbonate (0.361 g, 2.61 mmol) in 15 mL of dry acetonitrile, dry triethylamine (0.8 mL, 5.74 mmol) was added. The resulting mixture was stirred at room temperature for 30 min before filtering, evaporating to 2 mL, and pouring into 50 mL of diethyl ether. The resulting red oil was collected by decanting the solution then washing with ethyl acetate and diethyl ether. Drying in vacuo yielded crude compound [NBu_4_]_2_[**3**] as a dark-red solid (0.372 g, 0.216 mmol, 56%). Further purification of 90 mg of the sample was achieved by crystallization by diffusion of diethyl ether into an acetone solution, to give pure [NBu_4_]_2_[**3**] (0.041 g, 0.024 mmol) in an overall 26% yield. ^1^H NMR (500 MHz, (CD_3_)_2_CO): δ 7.18 (s, 2H), 6.88 (s, 2H), 3.98 (sept, *J* = 6.8 Hz, 2H), 3.47–3.44 (m, 16H), 3.21 (t, *J* = 5.7 Hz, 4H), 2.70 (t, *J* = 6.3, 4H), 1.92 (m, 4H), 1.86–1.80 (m, 16H), 1.45 (*a*sex, *J* = 6.8 Hz, 16H), 1.32 (d, *J* = 6.9 Hz, 12H), 0.98 (t, *J* = 7.3 Hz, 24H). ^13^C NMR (126 MHz, (CD_3_)_2_CO): δ 131.09, 125.29, 121.85, 59.37, 50.42, 28.17, 24.54, 24.16, 22.44, 20.41, 15.61, 13.96. Anal. calcd % for C_58_H_102_N_4_O_18_Mo_6_: C, 40.52 (40.86); H, 5.98 (5.95); N, 3.26 (3.24). HRMS (ESI, MeCN) *m*/*z*: 617.7923, calcd for C_26_H_30_N_2_Mo_6_O_18_^2–^ ([**3**]^2–^), 617.7933. FTIR (ATR) cm^–1^: 2960 (m), 2933 (sh), 2872 (m), 2187 (m), 1605 (sh), 1584 (m), 1511 (m), 1464 (m), 1380 (w), 1310 (m), 1249 (vw), 1208 (vw), 1185 (vw), 1145 (vw), 1170 (vw), 1052 (vw), 1030 (vw), 973 (m), 943 (s), 880 (m), 765 (s), 609 (w). UV–vis (MeCN) λ, nm (ε, M^–1^ cm^–1^): 212 (65.3 × 10^3^), 259 (30.3 × 10^3^), 308 (30.0 × 10^3^), 445 (37.8 × 10^3^).

### Preparation of [NBu_4_][Mo_6_O_17_(N_2_C_22_H_16_)_2_] ([NBu_4_]_2_[**4**])

To compound **P8** (0.116 g, 0.060 mmol) were added 4-ethynyl-*N*,*N*-dimethylaniline (0.029 g, 0.20 mmol), bis(triphenylphosphine) palladium(II) dichloride (8.5 mg, 0.012 mmol), copper iodide (5.2 mg, 0.027 mmol), potassium carbonate (0.597 g, 4.31 mmol), 8 mL dry acetonitrile, and triethylamine (0.8 mL, 5.74 mmol). The resulting red solution was stirred for 40 min to give a brown solution which was filtered, evaporated to ∼1 mL, and then precipitated with 15 mL of diethyl ether. The resulting dark-red precipitate was collected by filtration, washed with ethyl acetate and diethyl ether, and then dried in vacuo to yield compound [NBu_4_]_2_[**4**] as a dark-red solid (0.079 g, 0.040 mmol, 67%). ^1^H NMR (500 MHz, CD_3_CN): δ 7.36 (d, *J* = 9.0 Hz, 4H), 7.23 (s, 4H), 6.73 (d, *J* = 9.0 Hz, 4H), 3.88 (hept, *J* = 6.8 Hz, 16H), 3.11–3.03 (m, 16H), 2.89 (s, 12H), 1.65–1.55 (m, 16H), 1.36 (*a*sex, *J* = 7.4 Hz, 16H), 1.30 (d, *J* = 6.8 Hz, 24H), 0.96 (t, *J* = 7.4 Hz, 24H). ^13^C NMR (126 MHz, CD_3_CN): δ 149.12, 133.60, 125.74, 112.92, 59.36, 47.72, 40.38, 29.35, 24.34, 24.12, 20.35, 13.82. Anal. calcd % for C_76_H_124_N_6_O_17_Mo_6_: C, 46.35 (43.33); H, 6.35 (5.92); N, 4.27 (4.12). HRMS (ESI, MeCN): calcd for C_44_H_52_N_4_Mo_6_O_17_^2–^, 742.8852; found, 742.8844. FTIR (ATR) cm^–1^: 2960 (m), 2932 (sh), 2871 (m), 2810 (sh), 2188 (m), 1731 (w), 1606 (m), 1585 (s), 1521 (m), 1460 (m), 1444 (m), 1350 (m), 1242 (w), 1191 (m), 1150 (w), 1062 (w), 1043 (w), 965 (m), 942 (vs), 880 (m), 760 (vs). UV–vis (MeCN) λ, nm (ε, M^–1^ cm^–1^): 204 (108.7 × 10^3^), 246 (48.1 × 10^3^), 295 (50.6 × 10^3^), 420 (56.7 × 10^3^).

### Electrochemistry

Cyclic voltammetry and bulk electrolysis experiments were carried out using an Autolab PGstat 30 or PGstat 302 potentiostat/galvanostat. Measurements were performed in a three compartment cell using a silver wire reference electrode, a glassy carbon working electrode, and a platinum wire counter electrode. Acetonitrile was freshly distilled (from CaH_2_) and [N(C_4_H_9_-*n*)_4_]BF_4_^13^ was used as the supporting electrolyte. Solutions containing ca. 0.8 mM analyte (0.1 M electrolyte) were degassed by purging with argon and blanketed with a continuous flow of argon throughout the experiments. *E*_1/2_ values were calculated from (*E*_pa_ + *E*_pc_)/2 at a scan rate of 100 mV s^–1^ and referenced to Fc/Fc^+^. For bulk electrolysis, reduction of the bulk sample was achieved by application of a potential of −0.7 V vs Ag^0/+^ by a Pt gauze to the vigorously stirred solution for a total of 23 min. After this time, the current had plateaued at a current of near 0 A showing no further reduction was taking place.

### Spectroelectrochemistry

All measurements were performed using a Spectroelectrochemistry Reading RT OTTLE cell,^14^ with an Agilent Cary 60 UV–vis spectrophotometer and Autolab μ-III potentiostat. The analyte concentration was ca. 0.8 × 10^–3^ M in 0.3 M NBu_4_BF_4_ in dry acetonitrile. A reductive potential of between −0.8 and −0.5 V vs Ag was applied for between two and 5 min, varied to account for potential drift determined by a cyclic voltammogram taken immediately prior. UV–vis spectra were recorded at 15 s intervals. When no further changes were observed, a reoxidizing potential of −0.3 to 0 V vs Ag was applied for between two and 5 min until the continuous UV–vis monitoring showed no further changes.

### X-ray Crystallography

X-ray quality crystals of [NBu_4_]_2_[**1**], [NBu_4_]_2_[**2**]·0.25MeCN·0.25Et_2_O and [NBu_4_]_2_[**3**] and [NBu_4_]_2_[**4**], and iodo-precursor [NBu_4_]_2_[**P2**]·Et_2_O were grown by diffusion of diethyl ether into acetonitrile or acetone. Data were collected on a Rigaku XtalLab Synergy S diffractometer using a Photon-Jet Mo or Cu microfocus source and Hypix hybrid photon counting detector. Data reduction, cell refinement, and absorption correction were performed using Rigaku CrysAlisPro,^15^ and the structure was solved with SHELXT^16^ in Olex2 V1.5.^17^ Refinement was achieved by full-matrix least-squares on all *F*_0_^2^ data using SHELXL (v. 2018–3),^18^ also in Olex 2 V1.5. Full crystallographic data and refinement details are presented in Table S1 (in the Supporting Information) and ORTEP representations of the asymmetric units are provided in Figures S9–S13 (Supporting Information).

### Hyper-Rayleigh Scattering

General details of the hyper-Rayleigh scattering (HRS) experiment have been discussed elsewhere,^19^ and the experimental procedure and data analysis protocol used for the fs measurements used in this study were as previously described.^20^ Measurements were carried out using dilute (ca. 10^–5^ M) filtered (Millipore, 0.45 μm) acetonitrile solutions, such that self-absorption of the second harmonic (SH) signal was negligible, verified by the linear relation between signal and concentration. The 1064 nm source was a Spectra-Physics InSight DS+ laser (1 W average power, sub-100 fs pulses, 80 MHz). The collection optics were coupled to a spectrograph (model Bruker 500is/sm), together with an EMCCD camera (Andor Solis model iXon Ultra 897). Correction for multiphoton fluorescence (MPF) was done by subtracting the broad MPF background signal from the narrow HRS peak (fwhm ± 9 nm). The high accuracy and sensitivity of this setup enable us to use the solvent as an internal reference (acetonitrile, β_HRS,1064_ = 0.258 × 10^–30^ esu; β_*zzz*,1064_ = 0.623 × 10^–30^ esu).^21^ The depolarization ratio ρ for 2D compound [NBu_4_]_2_[**4**] was determined following established methods.^22^ β tensor components β_*zzz*_ and β_*zyy*_ were extracted by assuming Kleinman and planar symmetry for *C*_2v_ molecules, yielding only two significant components of the β tensor, β_*zzz*_ and β_*zyy*_. These are determined from orientationally averaged ⟨β_HRS_^2^⟩ and ρ by applying eqs 1 to 3(22c)
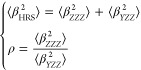
1

The HRS intensities with parallel polarization for fundamental and SH wavelengths, ⟨β_*ZZZ*_^2^⟩, and those for perpendicular polarization, ⟨β_*YZZ*_^2^⟩, can be expressed in terms of the molecular components β_*zzz*_ and β_*zyy*_ as follows
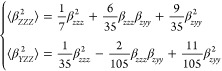
2

The depolarization ratio ρ can be expressed in terms of the ratio between the molecular β components, *k* = β_*zyy*_/β_*zzz*_.
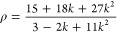
3

This approach can also be applied to non-*C*_2v_ geometries in which case it produces *effective* values of the tensor components that give an indication of the dimensionality of β response.

Electrochemically switched HRS measurements were performed as described previously,^11^ and further details are provided in the Supporting Information.

### Quantum Chemical Calculations

Geometry optimizations were performed by density functional theory (DFT), using the range-separated ωB97X-D exchange-correlation functional (XCF)^23^ with 6-311G(d)^24^ (C, H, N, O) and LANL2TZ^25^ (Mo) basis sets. Solvent effects (acetonitrile) were modeled using the integral equation formalism of the polarizable continuum model (IEF-PCM).^26^ The reliability of the ωB97X-D/6-311G(d)/LANL2TZ method for the geometry optimization of POM derivatives was demonstrated in comparison with other XC functionals in a previous work.^7e^ Excited-state properties were calculated for the optimized geometries using time-dependent density functional theory (TD-DFT),^27^ with the same XCF, basis set, and IEF-PCM solvation. The 30 lowest excitation energies, oscillator strengths, and transition dipole moments μ_ge_ were calculated together with the ground-to-excited state dipole moment changes Δμ_ge_, charge transfer distances *d*_CT_ and amounts of charge transferred *q*_CT_, according to the scheme presented by Le Bahers et al.^28^ Again with the same optimized geometries, XCF, basis set, and IEF-PCM scheme, SHG β tensor components were evaluated using the quadratic response TD-DFT method:^29^ ωB97X-D has been shown to be a reliable XCF for calculating the β tensors owing to its substantial amount of long-range HF exchange.^30^ Both static and dynamic (incident wavelength of 1064 nm) responses were calculated. For 2D anion [**4**]^2–^, the molecular response has been analyzed using the computed depolarization ratios and assuming Kleinman symmetry to obtain the two components β_*zzz*_ and β_*zyy*_, as described above for the HRS results. Further details of all computational aspects are provided in the Supporting Information.

## Results and Discussion

### Chromophore Design and Synthesis

**Figure 1:**
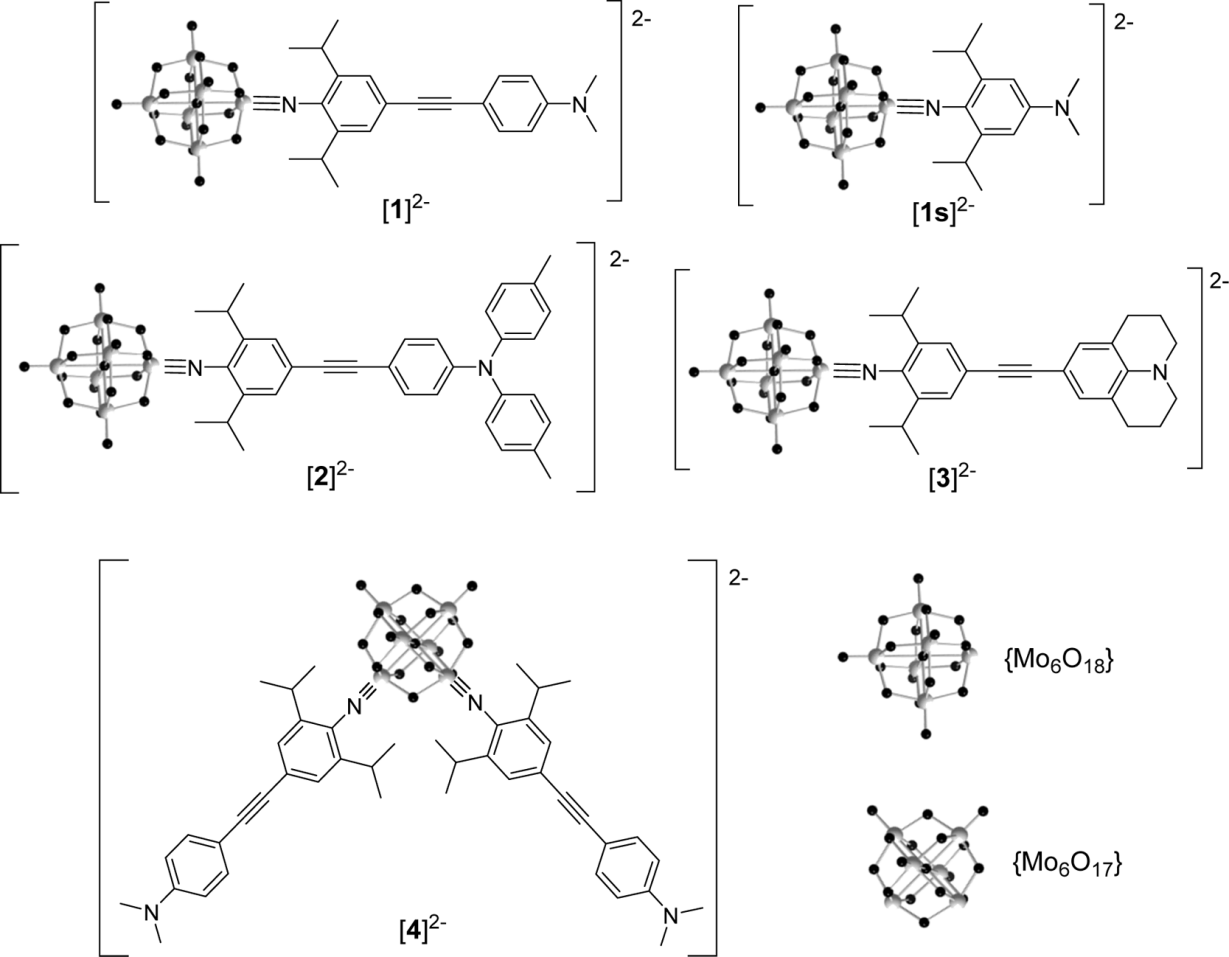
Structures of complex anions [**1**]^2–^ to [**4**]^2–^ synthesized for this study and previously published^11^ [**1s**]^2–^ included for comparison. All compounds were isolated as tetrabutylammonium salts.

Anions [**1**]^2–^ to [**4**]^2–^ (Figure 1) were designed to test the effects of donor type and chromophore geometry in isopropyl-protected arylimido hexamolybdate derivatives. In the linear series, these comprise a moderately strong resonance electron donor (−NMe_2_, [**1**]^2–^), a weakened resonance electron donor that allows delocalization of charge onto an electron-rich aromatic (−NTol_2_, [**2**]^2–^), and a very strong resonance electron donor (julolidinyl, Jd, [**3**]^2–^), which has previously yielded very high NLO activities in organic charge transfer (CT) chromophores.^31^ V-shaped (*C*_2v_) *bis*-imido derivative [**4**]^2–^ features the −NMe_2_ donor. The four compounds were obtained as their tetrabutylammonium salts by first synthesizing the required *mono*- or *bis*-(iodoarylimido) polyoxometalate derivatives [NBu_4_]_2_[**P2**] and [NBu_4_]_2_[**P3**] from tetrabutylammonium hexamolybdate using a well-established DCC-mediated imido coupling.^7,11,32^ We tested alternative, potentially more efficient syntheses of the *bis*-arylimido-functionalized anion [**P3**]^2–^ starting from [Mo_8_O_26_]^4–^,^33^ but in our hands, these yielded inseparable mixtures of products. Subsequently, Sonogashira protocols were used to attach the required donor group via an alkyne bridge (Scheme S1, Supporting Information).^ ^7a,7c^,33,33^ Identity and purity of the chromophores were confirmed using ^1^H NMR, ^13^C NMR, mass spectrometry, CHN analysis, and IR and UV–vis spectroscopy, and structures (including the *cis* substitution pattern in [**4**]^2–^) were also confirmed by X-ray diffraction.

^1^H NMR analysis of the *mono*-donor series (Figures S1–S3, Supporting Information) showed chemical shifts for the aromatic protons closest to the POM of 7.25 ppm for [**1**]^2–^, 7.28 ppm for [**2**]^2–^, and 7.20 ppm for [**3**]^2–^, consistent with the expected trend for the strength of the electron donors, with −NTol_2_ ([**2**]^2–^) shielding the protons least and Jd ([**3**]^2–^) shielding the most. Similarly, for [**4**]^2–^ (Figure S4), these protons are slightly more shielded (7.23 ppm) than for [**1**]^2–^ as *bis* functionalization of the POM core results in weaker electron withdrawal from the individual ligands by the POM. Similar but weaker effects are observed on the isopropyl CH_3_ protons of the four compounds.

### X-ray Crystallography

**Figure 2:**
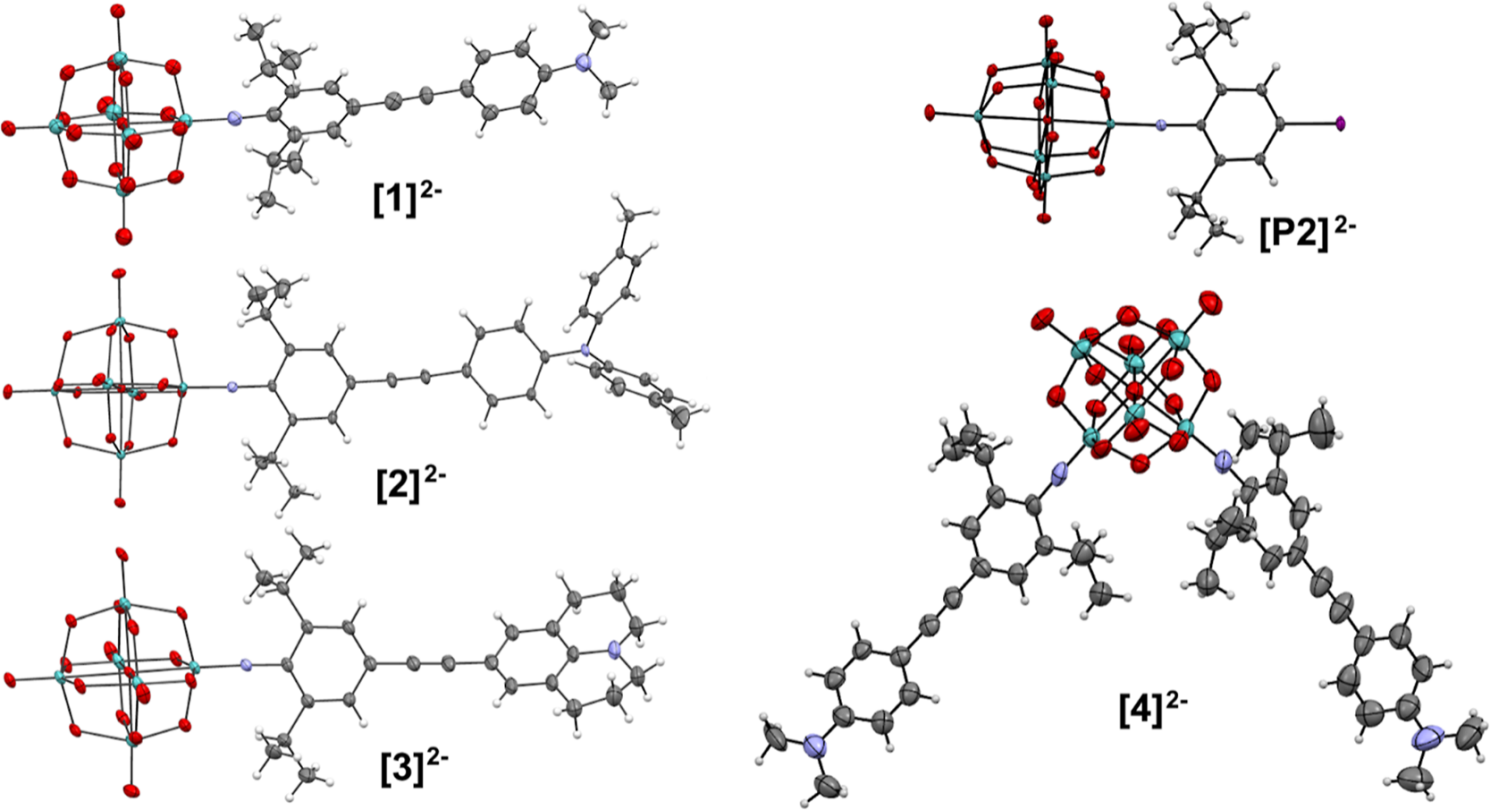
ORTEP representations of the complex anions in [NBu_4_]_2_[**P2**] and [NBu_4_]_2_[**1**] to [NBu_4_]_2_[**4**]. Thermal ellipsoids are drawn at the 30% probability level. Disorder in the julolidinyl group of [**3**]^2–^ has been omitted for clarity but is shown in Figure S12 (Supporting Information). Color scheme: C is gray; N, purple; O, red; Mo, green; I, purple. H atoms are represented by white spheres of arbitrary radii.

X-ray quality crystals were obtained for all four compounds and the precursor polyoxometalate [NBu_4_]_2_[**P2**], from ether diffusion into either acetone or acetonitrile solutions, enabling determination of crystal structures (Figures 2 and S9–S13 and Tables S1 and S2, Supporting Information). These confirm the *cis*-geometry of [NBu_4_]_2_[**4**]: both dipolar (*C*_2v_) *cis*- and centrosymmetric, quadrupolar *trans*-isomers of *bis*-substituted imido-Lindqvist species are possible,^34^ but show centrosymmetric (i.e., bulk SHG inactive) space groups for all compounds. Mo–O and Mo–N bond lengths (Table S2) of [NBu_4_]_2_[**P2**] and [NBu_4_]_2_[**1**] to [NBu_4_]_2_[**3**] are comparable to those seen for previous derivatized {Mo_6_} clusters, and while there is no significant trend in the length of the imido bonds, all three did show the increased linearity seen of previously published compounds featuring ^*i*^Pr groups adjacent to the imido bond, as steric repulsion between ^*i*^Pr and the POM restricts deviation of the Mo–N–C angle from 180°. Relatively low precision on bond lengths of [**1**]^2–^ and [**4**]^2–^ precludes in depth comparison of the structures of the π-bridges in this study and with those of prior derivatives, but it is worth noting that data obtained for *bis*-derivative [NBu_4_]_2_[**4**] do not indicate substantial differences to the bond lengths and angles observed for the *mono* analogue [NBu_4_]_2_[**1**]. The low-energy barriers of rotation in unhindered phenylacetylenes (ca. 1 kcal mol^–1^)^35^ result in both twisted and planar arrangements in crystal structures; however, previous crystallographic work has determined a pattern of increasingly planarity with weaker donor groups, ascribed to stronger conjugation in systems with less charge asymmetry.^7b^ Here, a similar trend is observed, with twist around the alkyne bond smallest in [**2**]^2–^, the weakest donor, and largest in [**3**]^2–^, the strongest donor. The average twist observed for the two donor arms of [**4**]^2–^ is very similar to that of [**1**]^2–^. Compared to previously published analogues with H atoms *ortho* to the imido groups instead of ^*i*^Pr, [**1**]^2–^ to [**4**]^2–^ all show a much less twisted diphenylacetylene bridge—for example, the 2,6-H analogue of **[1**]^2–^ shows a torsion angle of 86° vs 17.6° observed here. This suggests that the more linear imido bonds of the sterically protected compounds lead to increased conjugation through the π-bridge, although the influence of different crystal packing effects cannot be excluded.

### Electronic Spectroscopy and Electrochemistry

**Figure 3:**
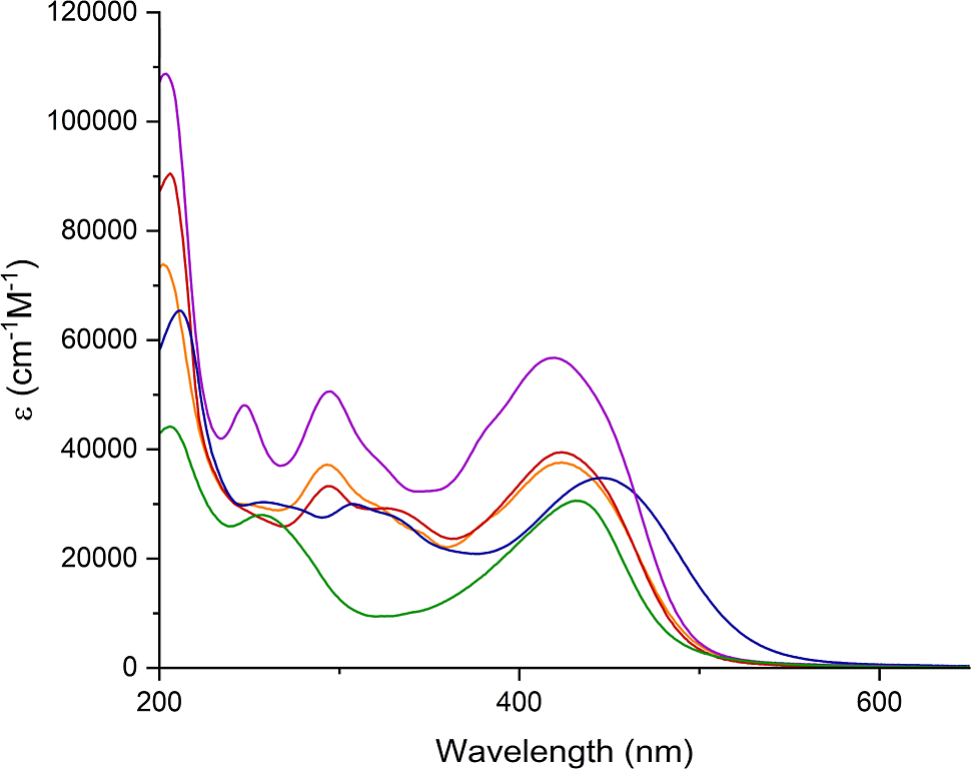
UV–vis spectra of compound [NBu_4_]_2_[**1**] (orange), [NBu_4_]_2_[**2**] (red), [NBu_4_]_2_[**3**] (blue), [NBu_4_]_2_[**4**] (purple), and [NBu_4_]_2_[**1s**] (green) obtained in acetonitrile.

UV–vis absorption spectra of [NBu_4_]_2_[**1**] to [NBu_4_]_2_[**4**] (Figure 3 and Table 1) all revealed Mo–O and π–π* peaks in the 200–300 nm region, with the higher peak extinction coefficients of [NBu_4_]_2_[**4**] a consequence of the second ligand—although notably, the absorption of this compound tails off rapidly so that there is no more absorption at the 532 nm second harmonic wavelength than for the other *mono*-derivatives. In addition, ligand-to-POM charge transfer (LPCT) peaks were seen in the 420–455 nm range for all four compounds. Compared to its phenyl-bridged analogue [NBu_4_]_2_[**1s**],^11^ the LPCT peak of compound [NBu_4_]_2_[**1**] shows a blue shift, from 431 to 424 nm. Commonly, extending conjugation red shifts CT transitions, but in arylimido-POMs with strong donors (−NMe_2_ and −NPh_2_), we have seen the opposite when a phenyl bridge is replaced by diphenylacetylene.^7d^ This is likely a consequence of weakened electronic communication due to free rotation around the alkyne bond, which reduces the extent of charge transfer onto the POM core. Spectrally, [NBu_4_]_2_[**1**] and [NBu_4_]_2_[**2**] are very similar, with only a 1 nm blue shift of the LPCT peak of compound **2** from 424 to 423 nm despite the slight decrease in donor strength. Larger blue shifts were previously observed on changing −NMe_2_ for −NPh_2_,^7c^ showing that the methyl groups in −NTol_2_ result in stronger electron donation. The LPCT peak of the julolidinyl derivative [NBu_4_]_2_[**3**] (445 nm) was red-shifted compared to [NBu_4_]_2_[**1**] and [NBu_4_]_2_[**2**] due to the stronger donor group, decreasing the transparency. The LPCT peak of [NBu_4_]_2_[**4**] is blue-shifted 4 nm from [NBu_4_]_2_[**1**], suggesting that addition of a second donor unit weakens the donor–acceptor character of the transition, by weaking the acceptor strength of the POM as reflected in a more negative redox potential for the first reduction (vide infra).

**Table 1:** UV–Vis Absorption and Electrochemical Data for the Oxidized and Reduced States of Compounds **1**, **2**, and **3** in Acetonitrile^d^

	λ_max_/nm^a^ (ε, 10^3^ M^–1^ cm^–1^)	*E*_max_/eV	*E*/V vs Fc/Fc^+^ (Δ*E*_p_/mV)^b^		
			*E*_pc_ [X]^3–/4–^	*E*_1/2_ or *E*_pc_ [X]^2–/3–^	*E*_pa_ [X]^1–/2–^
[**1**]^2–^	203 (73.6)	6.11	–1.837	–1.013 (85)	0.338 irr
	293 (37.1)	4.23			
	424 (37.6)	2.92			
[**1**]^3–^	202 (71.8)	6.14			
	296 (34.6)	4.19			
	406 (45.9)	3.05			
[**2**]^2–^	206 (90.4)	6.02	–1.905	–1.009 (73)	0.493 quas
	294 (33.1)	4.22			
	329 (19.0)	3.77			
	423 (39.4)	2.93			
[**2**]^3–^	204 (96.4)	6.08			
	296 (34.6)	4.19			
	406 (48.7)	3.05			
[**3**]^2–^	212 (65.3)	5.85	–1.831	–1.020 (85)	0.119 irr
	259 (30.3)	4.79			
	308 (30.0)	4.03			
	445 (37.8)	2.79			
[**3**]^3–^	210 (68.0)	5.90			
	308 (29.2)	4.03			
	414 (42.4)	2.99			
[**4**]^2–^	204 (108.7)	6.08		–1.056^c^	
	246 (48.1)	5.04			
	295 (50.6)	4.20			
	420 (56.7)	2.95			

a UV–vis data for [**1**/**2**/**3/4**]^2–^ were obtained from ca. 10^–5^ M solutions in MeCN. UV–vis data for [**1**/**2**/**3**]^3–^ were obtained from ca. 10^–2^ M solutions in 0.3 M [NBu_4_][BF_4_] using a thin layer spectroelectrochemistry cell, with ε for [**1**/**2**/**3**]^2–^ prior to reduction used to determine concentration.

b Analyte concentrations ca. 10^–3^ M in 0.1 M [NBu_4_][BF_4_] in MeCN glassy carbon working electrode, scan rate of 100 mV s^–1^.

c *E*_pc_, [**4**]^3–^ is unstable on the CV time scale.

d UV–vis Absorption and electrochemical data of compound **4** is included for the oxidized state only.

Cyclic voltammograms (Table 1 and Figure S14) of *mono*-derivatives [NBu_4_]_2_[**1**] to [NBu_4_]_2_[**3**] revealed [Mo_6_O_18_NAr]^2–/3–^ reduction peaks with near ideal reversibility and slight shifts in redox potential consistent with donor strength—the shifts are small as the diphenylethyne bridge (compared to phenyl) gives a degree of electronic isolation between the donor and POM core.^7b^ Logically, the irreversible or weakly reversible donor oxidations occurred at less positive potential for compounds with stronger donors, with oxidation of [**3**]^2–^ (Jd) occurring more than 380 mV more negative than [**2**]^2–^ (−NTol_2_). Reduction of [**4**]^2–^ (Figure S15) did not find the reversible [Mo_6_O_18_NAr]^2–/3–^ reduction typically seen of hexamolybdate derivatives, with the quasi-reversible [Mo_6_O_17_(NAr)_2_]^2–/3–^ reduction peak showing that significant decomposition occurred even at fast scan rates. Moreover, no [Mo_6_O_19_]^2–/3–^ peaks emerged, suggesting that decomposition of [**4**]^2–^ does not proceed through the solvolysis pathway observed for analogues without steric protection.^11,36^

**Figure 4:**
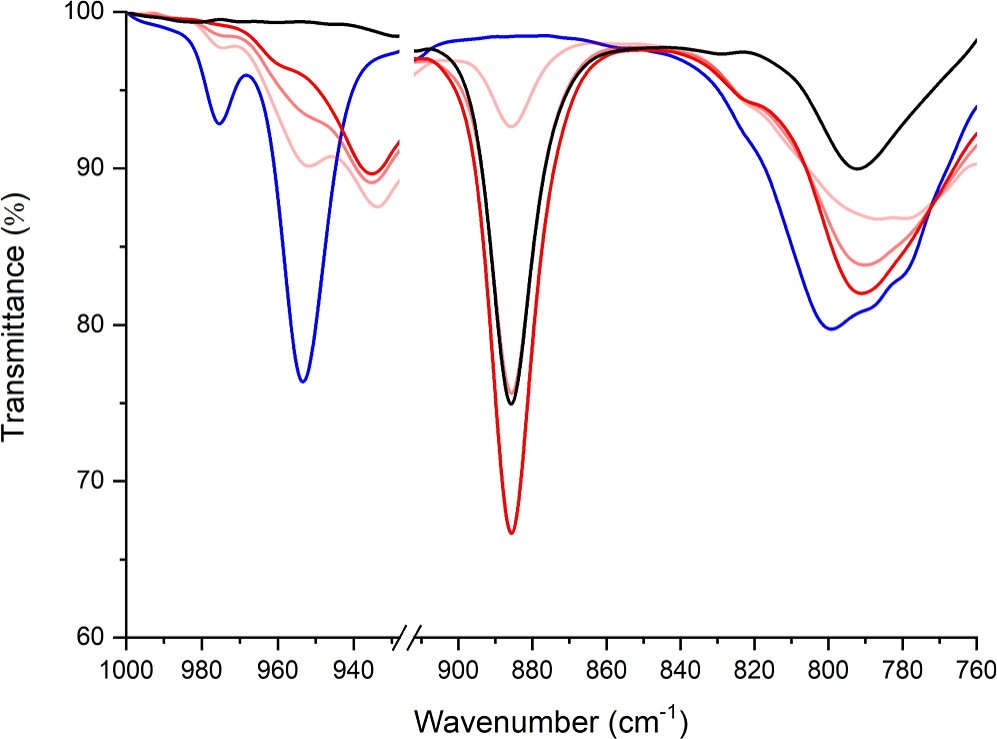
IR spectra of [NBu_4_]_2_[**1**] taken before (blue) and during (darkening red) with the spectra of [Mo_2_O_7_]^2–^ included (black) for comparison. All spectra were obtained in MeCN solution with supporting electrolyte 0.1 M [NBu_4_][BF_4_].

However, study of [NBu_4_]_2_[**1**] to [NBu_4_]_2_[**3**] by bulk electrolysis revealed greatly improved stability compared to previous compounds without ^*i*^Pr groups:^7,11,36^ after 23 min bulk electrolysis (near complete reduction), 83% of [**2**]^2–/3–^ remained and 75% of [**1**]^2–/3–^ and [**3**]^2–/3–^ (Figure S16)—compared to typically no more than 20% of compounds with only H in the 2 and 6 positions of the imido ring. Thus, the steric bulk of the ^*i*^Pr groups appears to be effective in protecting the imido bond from solvent or adventitious water. Interestingly, post-electrolysis cyclic voltammetry showed no signal for [Mo_6_O_19_]^2–/3–^ suggesting, as for [**4**]^3–^, decomposition was not occurring via the expected solvolysis pathway, and no other new electrochemically active species were observed. This suggested breakdown of {Mo_6_} to form other isopolyoxometalate species, many of which, as compounds with *cis*-terminal dioxo groups, show only destructive redox processes at highly negative potentials that can overlap with solvent reduction.^37^ IR analysis of a sample of [**1**]^2–/3–^ through a bulk electrolysis (Figure 4) showed emergence of new peaks at ca. 885 cm^–1^ and 790 cm^–1^ consistent with the presence of [Mo_2_O_7_]^2–^ and decomposition via fragmentation of the POM core, potentially after departure of a {MoNAr} subunit. Fragmentation of the hexamolybdate core of organoimido derivatives to smaller Mo-containing species including HMo_2_O_7_^–^ and {MoNAr} units has previously been observed in MS–MS experiments—most of all, for a system where the Ar unit carried a resonance (−OMe) donor.^38^ It is likely similar pathways are also responsible for the lower stability of [**4**]^3–^ and are encouraged by imido functionalization. Thus, slowing the hydrolysis/solvolysis pathway using steric bulk reveals slower decomposition pathways for the reduced {Mo_6_} unit that may be a result of increased charge asymmetry on the cluster due to the electron-donating imido fragment. In previously published phenyl-bridged, ^*i*^Pr-protected [NBu_4_][**1s**],^11^ this type of decomposition was much less evident, suggesting that the extended conjugated ligands play a role in enabling it—possibly by stabilizing or helping solvate the proposed {MoNAr} subunits. In future work, the stability of the reduced states may be improved by using noncoordinating, water-immiscible solvents—for example, dichloromethane—although the increased resistance of such generally less polar media can be a challenge in electrochemical experiments. The ideal solvent for redox-switching in solution would be highly polar, but noncoordinating, water-immiscible, and low viscosity.

**Figure 5:**
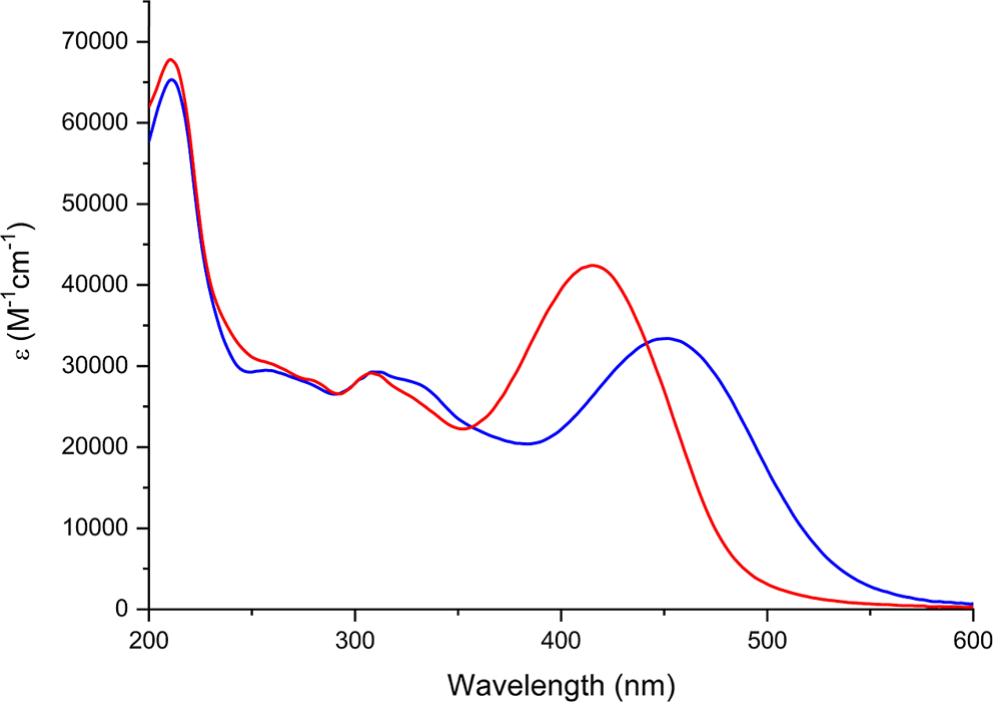
UV–vis spectra of [**3**]^2–^ (blue) and [**3**]^3–^ (red) obtained by spectroelectrochemistry (electrolyte 0.3 M NBu_4_BF_4_ in dry MeCN). A 97% recovery of the original spectrum was revealed upon reoxidation.

Study of the reduced anions [**1**]^3–^ to [**3**]^3–^by UV–vis spectroelectrochemistry (Figures 5 and S17) revealed a ca. 17 nm blue shift of the LPCT peaks of [**1**]^3–^ and [**2**]^3–^ relative to the parent dianions, and this increased to 31 nm for [**3**]^3–^. This shows that reduction of the POM anion lessens its ability as an electron acceptor, increasing the energy of the LPCT transition. A similar shift was previously observed for [**1s**]^2–/3–^, which showed reversible switching of the second-order NLO response.^11^ The change in the spectrum was reversible for all three *mono*-functionalized compounds, with 98% recovery of the absorbance of the LPCT peak for [NBu_4_]_2_[**2**] and 97% recovery for [NBu_4_]_2_[**1**] and [NBu_4_]_2_[**3**].

### Hyper-Rayleigh Scattering

Hyper-Rayleigh Scattering measurements performed at 1064 nm were used to investigate the NLO activity of [NBu_4_]_2_[**1**] to [NBu_4_]_2_[**4**], including depolarization measurements to determine the β components of [NBu_4_]_2_[**4**] (Table 2). Similar to previous studies with POMophores,^7b^ extending from phenyl-bridged −NMe_2_ donor [**1s**]^2–^ to phenylacetylene [**1**]^2–^ substantially enhances the response (close to 100%) while producing a blue shift rather than red shift in λ_max_. Changing to the −NTol_2_ donor [**2**]^2–^ increases β-values further, as previously observed for −NPh_2_,^7c,7d^ due to the extension of the HOMO onto the aryl groups, increasing ground-to-excited state dipole moment changes Δμ, and also the extension of the π-system strengthening communication right across the bridge. Both [**1**]^2–^ and [**2**]^2–^ show an increase (ca. 15%) in NLO activity over similar compounds without steric protection.^7b,7c^ This should be treated with caution, as it is within typical HRS measurement errors, but is consistent with the possibility that increased linearity of the Mo–N-Aryl linkage imposed by the ^*i*^Pr groups, and resulting strengthened communication through the π-system (suggested by smaller twist angles across the alkyne bond), plus increased electron density of the π-system due to the inductive effect of ^*i*^Pr, may be leading to increased β. Maximizing the electron donor strength with the julolidinyl group in [**3**]^2–^ produced the highest NLO activity of any POM chromophore to date, with a 10% increase in β_0,*zzz*_ and only a 7 nm increase in λ_max_ over a system with a stilbene bridge and −NPh_2_ donor.^7d^ This reveals maximizing donor strength, with ^*i*^Pr groups to control the imido-geometry and a diphenylacetylene bridge as an attractive approach to increasing β in POMophores, as synthetically such systems are more straightforward to access than stilbene-derivatized POMs.

**Table 2:** Experimental Values of Hyperpolarizability, β for [NBu_4_]_2_[**1**] to [NBu_4_]_2_[**4**], and [NBu_4_]_2_[**1s**] Measured by Hyper-Rayleigh Scattering in Acetonitrile^a^

	LPCT λ_max_ (nm)	β_HRS,1064_^b^ (×10^–30^ esu)	β_HRS,0_^c^ (×10^–30^ esu)	ρ	β_*zzz*,1064_ (×10^–30^ esu)	β_*zzz*,0_ (×10^–30^ esu)	β_*zyy*,1064_ (×10^–30^ esu)	β_*zyy*,0_ (×10^–30^ esu)
[NBu_4_]_2_[**1**]	424	220	68	n.d.	531^d^	162^f^	n.d.	n.d.
[NBu_4_]_2_[**1s**]	431	128	37	n.d.	309^d^	89^f^	n.d.	n.d.
[NBu_4_]_2_[**2**]	423	297	92	n.d.	717^d^	222^f^	n.d.	n.d.
[NBu_4_]_2_[**3**]	445	532	132	n.d.	1285^d^	318^f^	n.d.	n.d.
[NBu_4_]_2_[**4**]	420	260	83	3.83	654^e^	208^g^	–83.3^e^	–26.6^g^

a Data for [NBu_4_]_2_[**1s**] are taken from ref (11).

b Total molecular HRS response measured using 1064 nm fundamental laser beams. The quoted units (esu) can be converted into SI units (C^3^ m^3^ J^–2^) by dividing by a factor of 2.693 × 10^20^.

c Nonresonant, static β estimated from β_HRS_ using the two-state model.^39^

d Dynamic β_*zzz*_ derived assuming a single dominant tensor component due to linear, dipolar symmetry.

e Dynamic β tensor components derived from the HRS intensity and depolarization ratio, ρ.

f Static β_*zzz*_ derived assuming a single dominant tensor component due to linear, dipolar symmetry.

g Static β tensor components derived from the HRS intensity and depolarization ratio, ρ. Note that ρ for [NBu_4_]_2_[**4**] is high enough to be treated using a two-state model and a single dominant tensor component, yielding β_*zzz*,0_ = 192 × 10^–30^ esu with all other components zero.

Comparison of β_HRS,0_ of compound [**4**]^2–^ to that of monofunctionalized analogue [**1**]^2–^ reveals a 22% increase in orientationally averaged β_0,HRS_, from 68 × 10^–30^ esu to 83 × 10^–30^ esu. Combined with a decrease in λ_max_, this gives an excellent combination of activity and transparency. Measurement of a depolarization ratio and extraction of β_*zzz*_ and β_*zyy*_ tensor components reveals only a relatively minimally 2-dimensional response: β_*zyy*,0_, at −26.6 × 10^–30^ esu is only a little more than 10% of β_*zzz*,0_, likely a result of the relatively tight D···A···D angle of 90°. Nonetheless, this result shows promise for 2D D···A···D POMophores in increasing β without extending visible absorption profiles.

The large blue shift in linear absorption obtained for [NBu_4_]_2_[**3**] upon reduction (vide supra) implies a significant change in nonlinear optical properties, and as the compound with the highest oxidized state β_0,*zzz*_, it was decided to investigate the electrochemical switching of the HRS response for this compound. Reduction produced a 60% decrease in the intensity of the second harmonic scattered light over 400 seconds (Figure S18, Supporting Information). However, no plateau of the HRS signal intensity could be obtained, suggesting an ongoing degradation process forming byproducts with low, or zero activity, as well as the electrochemical reduction. Reoxidation restored 90% of the NLO activity of the sample, indicating that the NLO responses can be switched on and off, but combined with the bulk electrolysis data, it shows that [**3**]^3–^ is not sufficiently stable for multiple cycles under these conditions.

### DFT and TD-DFT Calculations

DFT and TD-DFT calculations were performed on anions [**1**]^2–^ to [**4**]^2–^ and their reduced states in Gaussian 2016 at the ωB97X-D, 6-311G(d)/LanL2TZ, IEF-PCM(acetonitrile) level of theory. Computed geometries (Table S3, Supporting Information) of the oxidized states generally show a good agreement with the experimentally determined X-ray crystal structures. Experimental data for the [**X**]^3–^ reduced-state structures is not available, but trends in computed oxidized and reduced-state electronic absorption spectra (vide infra) are a good match for spectroelectrochemical findings. The electronic structures (Figures S19–S23, Supporting Information) show for the oxidized states of all compounds a HOMO based on the ligand(s) and imido-group and a LUMO delocalized across the POM. Similar to previous findings for [**1s**]^3–^, for the reduced monodonor compounds [**1**]^3–^ to [**3**]^3–^, a spin-α HOMO–1 is found based on the POM, between 0.06 (−NMe_2_ donor [**1**]^3–^) and 0.31 eV (Jd donor [**3**]^3–^) lower in energy than a closely spaced pair of donor/aryl-imido-based HOMOs (one spin-α, one spin-β). In the reduced *bis*-donor system [**4**]^3–^, the increased electron density on the POM lifts the POM-based spin-α SOMO to higher energy than a group of four ligand-based HOMO/HOMO-x orbitals (spin-α and spin-β, located on each ligand). Spin-density calculations for the compounds indicate that the spin is either largely, or entirely located on the POM anions (Figure S24).

**Figure 6:**
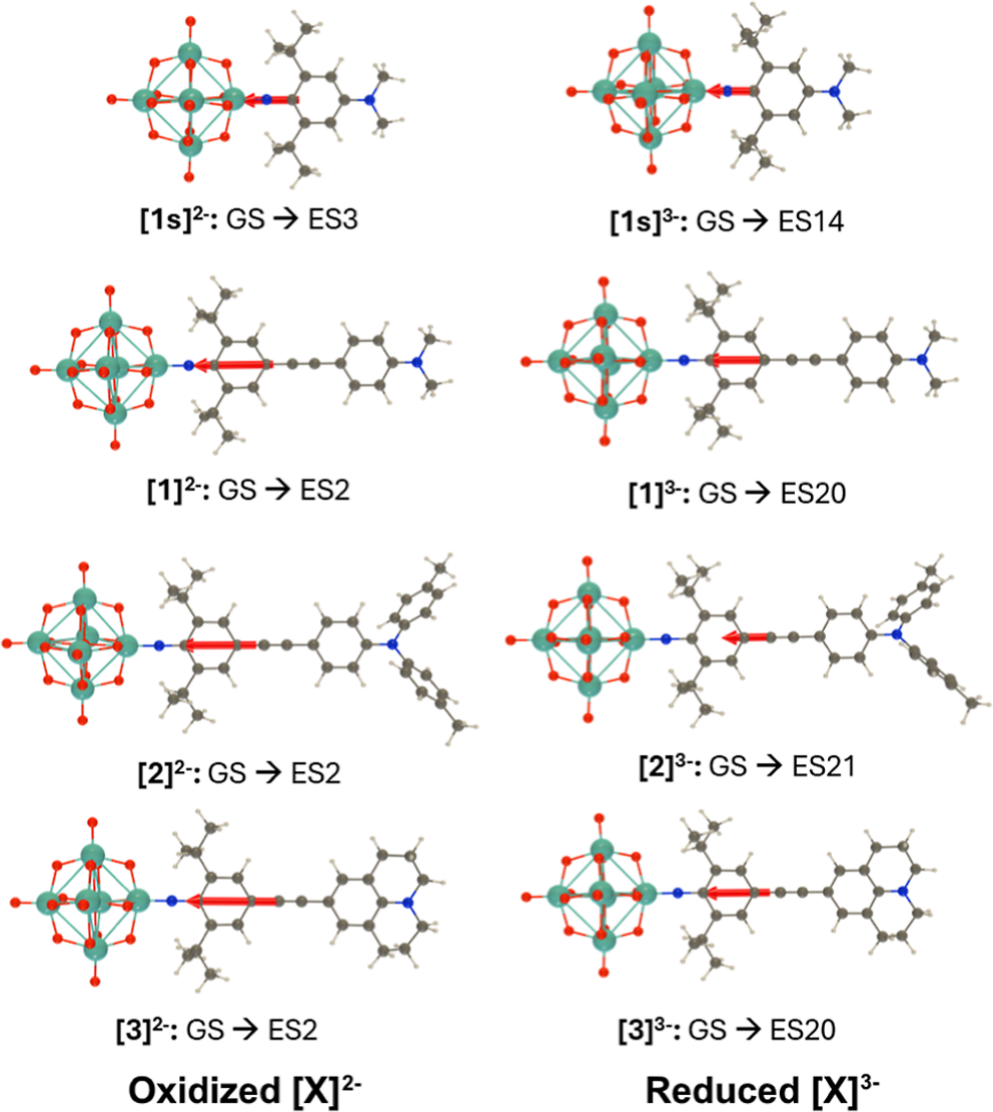
Charge transfer vectors (from the negative to the positive barycenter of Δρ) upon excitation from GS to the *n*th ES of the oxidized and reduced states of the 1-D anions [**1s**]^2–/3–^ and [**1**]^2–/3–^ to [**3**]^2–/3–^, as evaluated at the IEF-PCM(acetonitrile)/TDDFT/ωB97X-D/6-311G(d)/LanL2TZ level. The nonequilibrium solvation approach was adopted.

For the oxidized, monodonor species [**1**]^2–^ to [**3**]^2–^, trends in the TD-DFT computed vertical transition energies (Table 3 and Figure S25) match experimental observations—the red shift for the strong julolidinyl donor is smaller than observed experimentally, but vertical transition energies do not account for geometry relaxation of the excited state, or vibronic structure, that are pertinent to experimental measurements. The red, rather than experimentally observed blue shift in the lowest energy transition of V-shaped anion [**4**]^2–^ compared to [**1**]^2–^ is likely to be a result of the two bands (3.15 and 3.30 eV) overlapping in the experimental spectrum so that the peak is seen at higher energy. In all cases, calculated charge transfer vectors and changes in electron density distribution (Δρ) indicate CT from the arylimido ligands, toward the polyoxometalate, with increases in electron density at the POM and decreases on the donor (Figures 6 (left), S26, and S27); however, compared to previously studied analogues without isopropyl groups,^7e^ dipole moment changes Δμ_ge_ and charge transfer distances *d*_CT_ (Table S4) for [**1**]^2–^ and [**2**]^2–^ are lower, likely because the acceptor ring and POM are more electron rich. The powerful Jd donor group, however, gives the highest Δμ_ge_ yet computed for an arylimido-POM, consistent with its high β-value.

**Table 3:** TD-DFT-Computed Electronic Transitions, 1064 nm and Static β Responses for [NBu_4_]_2_[**1**] to [NBu_4_]_2_[**4**] and [NBu_4_]_2_[**1s**] in Acetonitrile^a^

	LPCT λ_max_^b^/nm	LPCT *E*_max_^b^ (f)/eV	β_HRS,1064_^c^/10^–30^ esu	β_*zzz*,1064_^c^/10^–30^ esu	β_*zyy*,1064_^c^/10^–30^ esu	β_HRS,0_^d^/10^–30^ esu	β_*zzz*,0_^d^/10^–30^ esu	β_*zyy*,0_^d^/10^–30^ esu
[**1]**^2–^	388	3.20 (1.98)	175	424^e^	n.d.	66.2	160^e^	n.d.
[**1**]^3–^	354	3.50 (1.93)	11.7	28.2^e^^,^^f^		36.4	88.0^e^	
[**1s**]^2–^	393	3.16 (1.02)	73.9	178^e^	n.d.	28.6	69.1^e^	n.d.
[**1s**]^3–^	369	3.36 (0.98)	45.8	111^e^		22.8	55.0^e^	
[**2**]^2–^	386	3.21 (2.25)	178	430^e^	n.d.	66.7	161^e^	n.d.
[**2**]^3–^	358	3.46 (2.25)	10.8	26.1^e^^,^^f^		34.3	82.7^e^	
[**3**]^2–^	397	3.12 (2.06)	245	593^e^	n.d.	85.2	206^e^	n.d.
[**3**]^3–^	360	3.44 (1.95)	33.3	80.3^e^^,^^f^		47.3	114^e^	
[**4**]^2–^	393	3.15 (2.08)	213	528^g^	–38.3^g^	80.4	194^g^	–19.6^g^
	376	3.30 (1.68)						
[**4**]^3–^	379	3.27 (1.22)	112	277^g^	–19.2^g^	46.1	111^g^	–14.1^g^
	378	3.28 (0.34)						
	352	3.52 (1.50)						
	350	3.54 (0.31)						

a All calculations carried out by TD-DFT at the ωB97X-D/6-311G(d)/LanL2TZ level of theory with acetonitrile solvation by IEFPCM. β-Values are reported following B-convention to facilitate comparison with experiment.

b Vertical transitions.

c Dynamic HRS β responses, calculated at 1064 nm.

d Static response extracted from calculations at λ = 1500 nm, to avoid resonance effects.

e β_*zzz*_ calculated assuming a single dominant tensor component.

f Large difference vs [**X**]^2–^ state is a consequence of resonance effects.

g Tensor components deduced from computed ρ and β_HRS_ values, assuming planar *C*_2v_ symmetry. Alternative treatment of [**4**]^2–^ assuming a single dominant tensor component yields β_*zzz*_,_1064_ = 513 × 10^–30^ esu with all other components zero.

Consistent with experiment, the computed LPCT UV–vis absorption bands of the reduced-state spectra ([**1**]^3–^ to [**3**]^3–^) show blue shifts (Table 3 and Figure S28), versus the oxidized states. Weaker, low-energy peaks in the computed spectra are at energies consistent with reported experimental spectra for [Mo_6_O_19_]^3–^,^40^ concentrations used in spectroelectrochemical measurements were too low for detection of these peaks, which may be merged with the absorption tail of the LPCT band. Similar to the experimental data, the largest blue shift is observed for [**3**]^3–^ vs [**3**]^2–^; however, the computed blue shifts are generally larger than those observed by experiment. This, and the tendency of experimental ε values to increase upon reduction, while computed oscillator strengths decrease slightly, is likely a consequence of differences in geometry relaxation and vibronic structure between the oxidized and reduced states, which are not captured by the calculation. Computed charge transfer vectors (Figures 6 and S26), dipole moment changes (Table S4), and changes in electron density (Figures S19 and S27) show that CT is still from the ligand to POM, but less strongly, with Δμ_ge_ dropping more dramatically from [**X**]^2–^ to [**X**]^3–^ state for the new, extended systems [**1**]^2–/3–^ to [**4**]^2–/3–^ than for previously studied, phenyl-bridged [**1s**]^2–^.

Computed dynamic first hyperpolarizabilities β_HRS,1064_ and β_*zzz*,1064_ of the oxidized species (Table 3) follow experimental trends in finding that of the phenylacetylene-bridged systems [**1**]^2–^ to [**4**]^2–^, *mono*-NMe_2_ donor [**1**]^2–^ has the lowest activity, and julolidinyl derivative [**3**]^2–^ the highest. Generally, similar to previous studies, the computed values are lower than those found experimentally, most notably for [**2**]^2–^ and [**3**]^2–^. In the case of [**3**]^2–^, it is possible that the more red-shifted absorption profile leads to an increased resonance contribution to the experimental result, but the computed value still represents a new record for a POMophore. For [**2**]^2–^, there is no red-shift vs [**1**]^2–^, but we have noted previously that computed β-values tend to be underestimated for −NAr_2_ donors with diphenylacetylene and stilbene bridges. As found by experiment, adding a second donor arm ([**4**]^2–^ vs [**1**]^2–^) yields a ca. 20% increase in activity (orientationally averaged β_HRS_), and computationally determined tensor components β_*zzz*_ and β_*zyy*_ for this *C*_2v_ anion are similar to experiment in showing only very minimal 2D character in the response, which can be adequately described by a single tensor component β_*zzz*_. Computed static hyperpolarizabilities β_0_ are also shown in Table 3, extracted from values calculated at 1500 nm, away from resonance effects. Generally, these are closer in magnitude to experimental values than the dynamic 1064 nm ones and follow experimental trends, although values for −NTol_2_ and Jd donors are comparatively underestimated.

**Figure 7:**
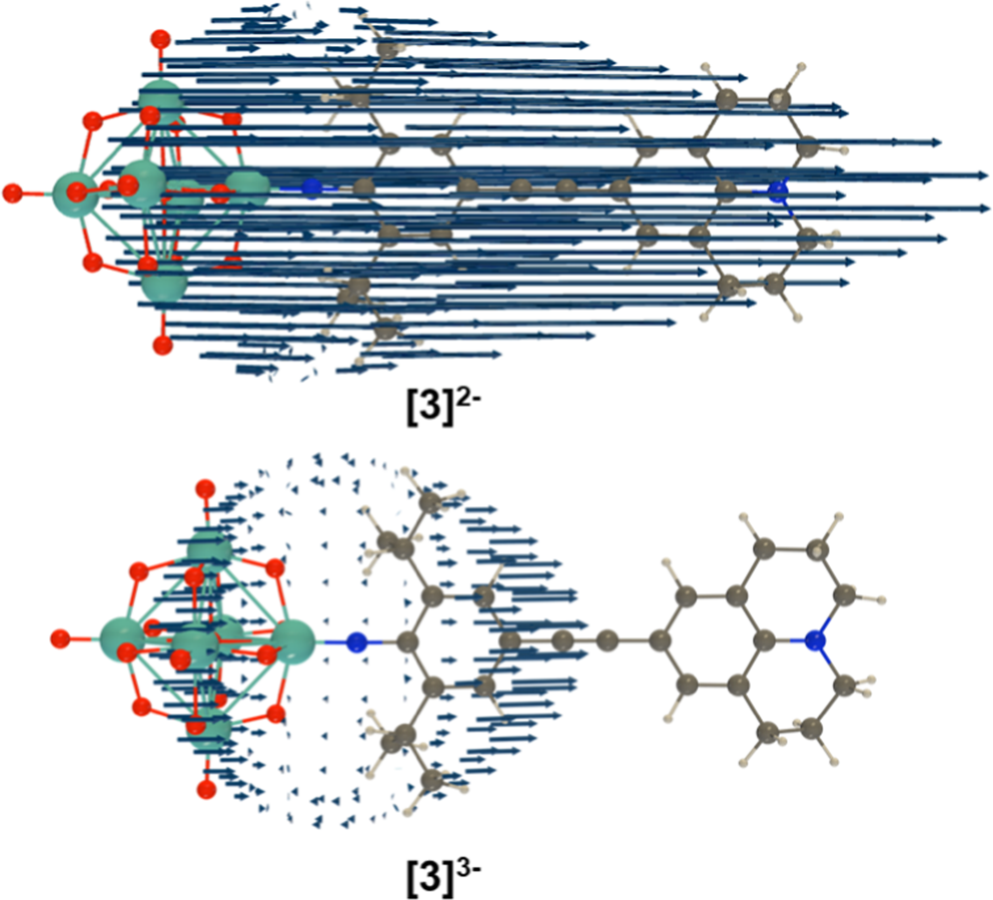
Unit sphere representation (USR) of the first hyperpolarizability tensor (λ = 1064 nm) of [**3**]^2–^ and [**3**]^3–^ as calculated at the IEFPCM (solvent = acetonitrile) TDDFT/wB97X-D/6-311G(d)/LanL2TZ level of approximation. Factor = 10^–4^ Å/a.u._β_.

In addition to the oxidized species, hyperpolarizabilities have been calculated for the reduced states [**X**]^3–^. These show that in all cases, the increase in CT transition energies caused by reduction of the POM feeds through into lowered β values. Decreases in β_0_ are around 50%, as signal intensity is proportional to β^2^, this would be consistent with a 75% decrease in signal intensity in the absence of resonance effects. Larger changes computed for β_1064_ in [**1**]^2–/3–^ to [**3**]^2–/3–^ result from resonance effects that may not be experimentally observed—depending on the accuracy of computed absorption profiles—and are not pertinent to all wavelengths. Thus, the experimental 60% decrease in signal obtained for reduction of [**3**]^2–^ to [**3**]^3–^ is consistent with the calculated changes in β. The origin of the decreased β values is primarily the smaller (by up to 45%) Δμ_ge_ found for the reduced states, and the effect of this on the β tensors is graphically represented for [**3**]^2–/3–^ (Figure 7) and the other compounds (Figure S30) in unit sphere representations. The relatively low on/off contrast computed across the series, and observed experimentally for [**3**]^2–/3–^, is consistent with delocalization of charge across the {Mo_6_O_18_N} cluster—one electron reduction results in an average oxidation state change of only −0.167 per Mo, and an increase in average d-orbital occupancy from 0 to 0.167, so that switch off of LPCT will be far from complete. Thus, it seems likely that the much larger contrast experimentally reported^11^ for [**1s**]^2–/3–^ is partly a result of different resonance and absorption effects in the two states.

## Conclusions

A family of new, sterically protected POM-based charge transfer chromophores with diphenylacetylene bridges has been synthesized. All four new compounds show high second-order nonlinear optical activity, without long wavelength absorption (λ_max_ < 450 nm in all cases), and using a julolidine donor system produced the highest β_0,*zzz*_ yet reported for this class of compounds, with only a small red shift compared to the next highest performing. Moreover, extending the study to include a 2-dimensional (V-shaped) CT system based on a *C*_2v_*bis*-donor POM indicates that such derivatives can have higher NLO activity than 1-D dipolar analogues, with no red shift in absorption profile. Redox-switched HRS experiments on the julolidinyl derivatized compound demonstrated reversible responses from a higher starting activity than seen previously; however, contrast was less than previously reported for an −NMe_2_ donor system with a phenyl bridge. Nonetheless, TD-DFT calculations suggest that extended conjugated systems may increase on/off contrast and indicate that experimentally observed contrast may be strongly affected by resonance effects. The reduced states of the extended species studied were not sufficient for multicycle switching in solution, likely due to a decomposition mechanism that results in production of [Mo_2_O_7_]^2–^ and appears to be favored by extended arylimido ligands. Consequently, while steric bulk around the imido-group clearly stabilizes arylimido-polyoxometalate-reduced states, switched second-order NLO contrast and multicycle stability appear to vary quite strongly with the imido-aryl ligand. Work to better understand this, mitigate decomposition processes, and extend our study to more multidimensional POM-based chromophores is ongoing.

## Data Availability

In addition to the supporting information and deposited cif files, data can be obtained by contacting the corresponding author and will be deposited at DOI: 10.17635/Lancaster/researchdata/705.

## Supporting Information Available

Full synthetic details for organic precursor compounds, including characterization, X-ray crystallographic details including tabulated bond lengths, cyclic voltammograms, full computational details including calculated bond lengths, UV–vis spectra, and CT descriptors (PDF)
Cartesian coordinates of computed geometries (ZIP)

## Abbreviation

POM: polyoxometalate

## References

[1] aPolyoxometalates: from Platonic Solids to Anti-Retroviral Activity; Pope M. T., Müller A., Eds.; Kluwer Academic Publishers: Dordrecht, The Netherlands, 1994.

[2] aMüller A.; Krickenmeyer E.; Bögge H.; Schmidtmann M.; Peters F. Organizational Forms of Matter: An Inorganic Super Fullerene and Keplerate Based on Molybdenum Oxide. Angew. Chem., Int. Ed. 1998, 37, 3359–3363. 10.1002/(SICI)1521-3773(19981231)37:24<3359::AID-ANIE3359>3.0.CO;2-J. 29711296

[3] aNeumann R.; Dahan M. A Ruthenium-Substituted Polyoxometalate as an Inorganic Dioxygenase for Activation of Molecular Oxygen. Nature 1997, 388, 353–355. 10.1038/41039.

[4] aBijelic M.; Aureliano A.; Rompel A. Polyoxometalates as Potential Next-Generation Metallodrugs in the Combat Against Cancer. Angew. Chem., Int. Ed. 2019, 58, 2980–2999. 10.1002/anie.201803868. PMC6391951 29893459

[5] aBusche C.; Vilà-Nadal L.; Yan J.; Miras H. N.; Long D.-L.; Georgiev V. P.; Asenov A.; Pedersen R. H.; Gadegaard N.; Mirza M. M.; Paul D. J.; Poblet J. M.; Cronin L. Design and Fabrication of Memory Devices based on Nanoscale Polyoxometalate Clusters. Nature 2014, 515, 545–549. 10.1038/nature13951. 25409147

[6] aDolbecq A.; Dumas E.; Mayer C. R.; Mialane P. Hybrid Organic–Inorganic Polyoxometalate Compounds: From Structural Diversity to Applications. Chem. Rev. 2010, 110, 6009–6048. 10.1021/cr1000578. 20666374

[7] aAl-Yasari A.; Van Steerteghem N.; El Moll H.; Clays K.; Fielden J. Donor–Acceptor Organo-Imido Polyoxometalates: High Transparency, High Activity Redox-Active NLO Chromophores. Dalton Trans. 2016, 45, 2818–2822. 10.1039/C6DT00115G. 26815652

[8] aNonlinear Optics of Organic Molecules and Polymers; Nalwa H. S., Miyata S., Eds.; CRC Press: Boca Raton, FL, 1997.

[9] aKang H.; Facchetti A.; Jiang H.; Cariati E.; Righetto S.; Ugo R.; Zuccaccia C.; Macchioni A.; Stern C. L.; Liu Z.; Ho S. T.; Brown E. C.; Ratner M. A.; Marks T. J. Ultralarge Hyperpolarizability Twisted π-Electron System Electro-Optic Chromophores: Synthesis, Solid-State and Solution-Phase Structural Characteristics, Electronic Structures, Linear and Nonlinear Optical Properties, and Computational Studies. J. Am. Chem. Soc. 2007, 129, 3267 10.1021/ja0674690. 17309258

[10] aCoe B. J.; Houbrechts S.; Asselberghs I.; Persoons A. Efficient, Reversible Redox-Switching of Molecular First Hyperpolarizabilities in Ruthenium(II) Complexes Possessing Large Quadratic Optical Nonlinearities. Angew. Chem., Int. Ed. 1999, 38, 366–369. 10.1002/(SICI)1521-3773(19990201)38:3<366::AID-ANIE366>3.0.CO;2-D. 29711640

[11] Hood B. R.; de Coene Y.; Torre Do Vale Froes A. V.; Jones C. F.; Beaujean P.; Liégeois V.; MacMillan F.; Champagne B.; Clays K.; Fielden J. Electrochemically-Switched 2nd Order Non-Linear Optical Response in an Arylimido-Polyoxometalate with High Contrast and Cyclability. Angew. Chem., Int. Ed. 2023, 62, e202215537 10.1002/anie.202215537. PMC10107823 36448963

[12] Klemperer W. G. Tetrabutylammonium Isopolyoxometalates. Inorg. Synth. 1990, 27, 74–85. 10.1002/9780470132586.ch15.

[13] Ibrahim S. K.. Ph.D. Thesis, University of Sussex, 1992.

[14] Krejčik M.; Daněk M.; Hartl F. Simple Construction of an Infrared Optically Transparent Thin-Layer Electrochemical Cell: Applications to the Redox Reactions of Ferrocene, Mn_2_(CO)_10_ and Mn(CO)_3_(3,5-di-t-Butyl-catecholate)^−^. J. Electroanal. Chem. 1991, 317, 179–187. 10.1016/0022-0728(91)85012-E.

[15] CrysAlisPro, (Version 1.171.40.68a); Rigaku Oxford Diffraction, Rigaku Corporation: Tokyo, Japan, 2019.

[16] Sheldrick G. M. SHELXT—Integrated Space-Group and Crystal-Structure Determination. Acta Crystallogr., Sect. A: Found. Adv. 2015, 71, 3–8. 10.1107/S2053273314026370. 25537383 PMC4283466

[17] Dolomanov O. V.; Bourhis L. J.; Gildea R. J.; Howard J. A. K.; Puschmann H. OLEX2: A Complete Structure Solution, Refinement and Analysis Program. J. Appl. Crystallogr. 2009, 42, 339–341. 10.1107/S0021889808042726.

[18] Sheldrick G. M. Crystal Structure Refinement with SHELXL. Acta Crystallogr., Sect. C: Struct. Chem. 2015, 71, 3–8. 10.1107/S2053229614024218. 25567568 PMC4294323

[19] aClays K.; Persoons A. Hyper-Rayleigh Scattering in Solution. Phys. Rev. Lett. 1991, 66, 2980–2983. 10.1103/PhysRevLett.66.2980. 10043668

[20] aOlbrechts G.; Strobbe R.; Clays K.; Persoons A. High-Frequency Demodulation of Multi-Photon Fluorescence in Hyper-Rayleigh Scattering. Rev. Sci. Instrum. 1998, 69, 2233–2241. 10.1063/1.1148926.

[21] Campo J.; Desmet F.; Wenseleers W.; Goovaerts E. Highly Sensitive Setup for Tunable Wavelength Hyper-Rayleigh Scattering with Parallel Detection and Calibration Data for Various Solvents. Opt. Express 2009, 17, 4587–4604. 10.1364/OE.17.004587. 19293888

[22] aHeesink G. J. T.; Ruiter A. G. T.; van Hulst N. F.; Bölger B. Determination of Hyperpolarizability Tensor Components by Depolarized Hyper-Rayleigh Scattering. Phys. Rev. Lett. 1993, 71, 999–1002. 10.1103/PhysRevLett.71.999. 10055423

[23] Chai J. D.; Head-Gordon M. Long-Range Corrected Hybrid Density Functionals with Damped Atom-Atom Dispersion Corrections. Phys. Chem. Chem. Phys. 2008, 10, 6615–6620. 10.1039/b810189b. 18989472

[24] Krishnan R.; Binkley J. S.; Seeger R.; Pople J. A. Self-Consistent Molecular Orbital Methods. XX. A Basis Set for Correlated Wavefunctions. J. Chem. Phys. 1980, 72, 650–654. 10.1063/1.438955.

[25] Roy L. E.; Hay P. J.; Martin R. L. Revised Basis Sets for the LANL Effective Core Potentials. J. Chem. Theory Comput. 2008, 4, 1029–1031. 10.1021/ct8000409. 26636355

[26] Tomasi J.; Mennucci B.; Cammi R. Quantum Mechanical Continuum Solvation Models. Chem. Rev. 2005, 105, 2999–3094. 10.1021/cr9904009. 16092826

[27] Casida M. E.. In Recent Advances in Density Functional Theory; Chong D. P., Ed.; World Scientific: Singapore, 1995; pp 155–192.

[28] Le Bahers T.; Adamo C.; Ciofini I. A. Qualitative Index of Spatial Extent in Charge-Transfer Excitations. J. Chem. Theory Comput. 2011, 7, 2498–2506. 10.1021/ct200308m. 26606624

[29] aVan Gisbergen S. J. A.; Snijders J. G.; Baerends E. J. Calculating Frequency-Dependent Hyperpolarizabilities using Time-Dependent Density Functional Theory. J. Chem. Phys. 1998, 109, 10644–10656. 10.1063/1.477762.

[30] ade Wergifosse M.; Champagne B. Electron Correlation Effects on the First Hyperpolarizability of Push-Pull π-Conjugated Systems. J. Chem. Phys. 2011, 134, 074113 10.1063/1.3549814. 21341834

[31] Coe B. J.; Fielden J.; Foxon S. P.; Harris J. A.; Helliwell M.; Brunschwig B. S.; Asselberghs I.; Clays K.; Garín J.; Orduna J. Diquat Derivatives: Highly Active, Two-Dimensional Nonlinear Optical Chromophores with Potential Redox Switchability. J. Am. Chem. Soc. 2010, 132, 10498–10512. 10.1021/ja103289a. 20617798

[32] aWei Y.; Xu B.; Barnes C. L.; Peng Z. An Efficient and Convenient Reaction Protocol to Organoimido Derivatives of Polyoxometalates. J. Am. Chem. Soc. 2001, 123, 4083–4084. 10.1021/ja004033q. 11457161

[33] Xu L.; Lu M.; Xu B.; Wei Y.; Peng Z.; Powell D. R. Towards Main-Chain-Polyoxometalate-Containing Hybrid Polymers: A Highly Efficient Approach to Bifunctionalized Organoimido Derivatives of Hexamolybdates. Angew. Chem., Int. Ed. 2002, 41, 4129–4132. 10.1002/1521-3773(20021104)41:21<4129::AID-ANIE4129>3.0.CO;2-R. 12412104

[34] aXia Y.; Wei Y.; Wang Y.; Guo H. A Kinetically Controlled Trans Bifunctionalized Organoimido Derivative of the Lindqvist-Type Hexamolybdate: Synthesis, Spectroscopic Characterization, and Crystal Structure of (*n*-Bu_4_N)_2_{*trans*-[Mo_6_O_17_(NAr)_2_]. Inorg. Chem. 2005, 44, 9823–9828. 10.1021/ic051319t. 16363852

[35] aLin V. S.; Therien M. J. The Role of Porphyrin-to-Porphyrin Linkage Topology in the Extensive Modulation of the Absorptive and Emissive Properties of a Series of Ethynyl- and Butadiynyl-Bridged Bis- and Tris(porphinato)zinc Chromophores. Chem.—Eur. J. 1995, 1 (9), 645–651. 10.1002/chem.19950010913.

[36] Al-Yasari A. Synthesis, Non-Linear Optical and Electrochemical Properties of Novel Organoimido Polyoxometalate Derivatives. Ph.D. Thesis, University of East Anglia, 2016.

[37] Weinstock I. A. Homogeneous-Phase Electron-Transfer Reactions of Polyoxometalates. Chem. Rev. 1998, 98, 113–170. 10.1021/cr9703414. 11851501

[38] Cao J.; Wang Q.; Liu C.; An S. Gas-Phase Chemistry of Arylimido-Functionalized Hexamolybdates [Mo_6_O_19_]^2–^. J. Am. Soc. Mass Spectrom. 2018, 29, 1331–1334. 10.1007/s13361-018-1948-4. 29671275

[39] aOudar J. L.; Chemla D. S. Hyperpolarizabilities of the Nitroanilines and their Relations to the Excited State Dipole Moment. J. Chem. Phys. 1977, 66, 2664–2668. 10.1063/1.434213.

[40] Che M.; Fournier M.; Launay J. P. The Analog of Surface Molybdenyl Ion in Mo/SiO_2_ supported catalysts: the Isopolyanion Mo_6_O_19_^3–^ Studied by EPR and UV-Visible Spectroscopy. Comparison with Other Molybdenyl Compounds. J. Chem. Phys. 1979, 71, 1954–1960. 10.1063/1.438549.

