# Supplementary material for: Donor and Geometry Optimization: Fresh Perspectives for the Design of Polyoxometalate Charge Transfer Chromophores
Source: Inorg Chem. 2025 Apr 14;64(16):8408–20. doi: 10.1021/acs.inorgchem.5c00915 (PMC12042256; doi:10.1021/acs.inorgchem.5c00915)
Supplement: Supplementary file 1 — ic5c00915_si_001.pdf [file ic5c00915_si_001.pdf]

***Supporting Information for*:**

Donor and Geometry Optimization: Fresh Perspectives for the Design of Polyoxometalate Charge Transfer Chromophores

Bethany R. Hood,[a,b] Yovan de Coene,[d] Claire F. Jones,[c] Noah Deveaux,[e] Jack M. Barber,[b] Charlotte G. Marshall,[b] Chloe A. Jordan,[b] Nathan R. Halcovitch,[a] Benoît Champagne,*[e] Koen Clays*[e] and John Fielden.*[a,b]

[a] Department of Chemistry, Lancaster University, Lancaster, United Kingdom, LA1 4YB. E-mail: j.fielden@lancaster.ac.uk.
[b] School of Chemistry, University of East Anglia, Norwich, United Kingdom, NR4 7TJ.
[c] School of Chemistry, Pharmacy and Pharmacology, University of East Anglia, Norwich, United Kingdom, NR4 7TJ.
[d] Department of Chemistry, University of Leuven, Celestijnenlaan 200D, 3001 Leuven, Belgium. E-mail: Koen.Clays@kuleuven.be.
[e] Unit of Theoretical and Structural Physical Chemistry, Namur Institute of Structured Matter, University of Namur, B-5000 Namur, Belgium. E-mail: Benoit.Champagne@unamur.be.

1. NMR and HRMS spectra

Presented below are ^{1}H-NMR and HRMS spectra of the new polyoxometalate derivatives $[NBu_4]_2$[**1**] to $[NBu_4]_2$[**4**].

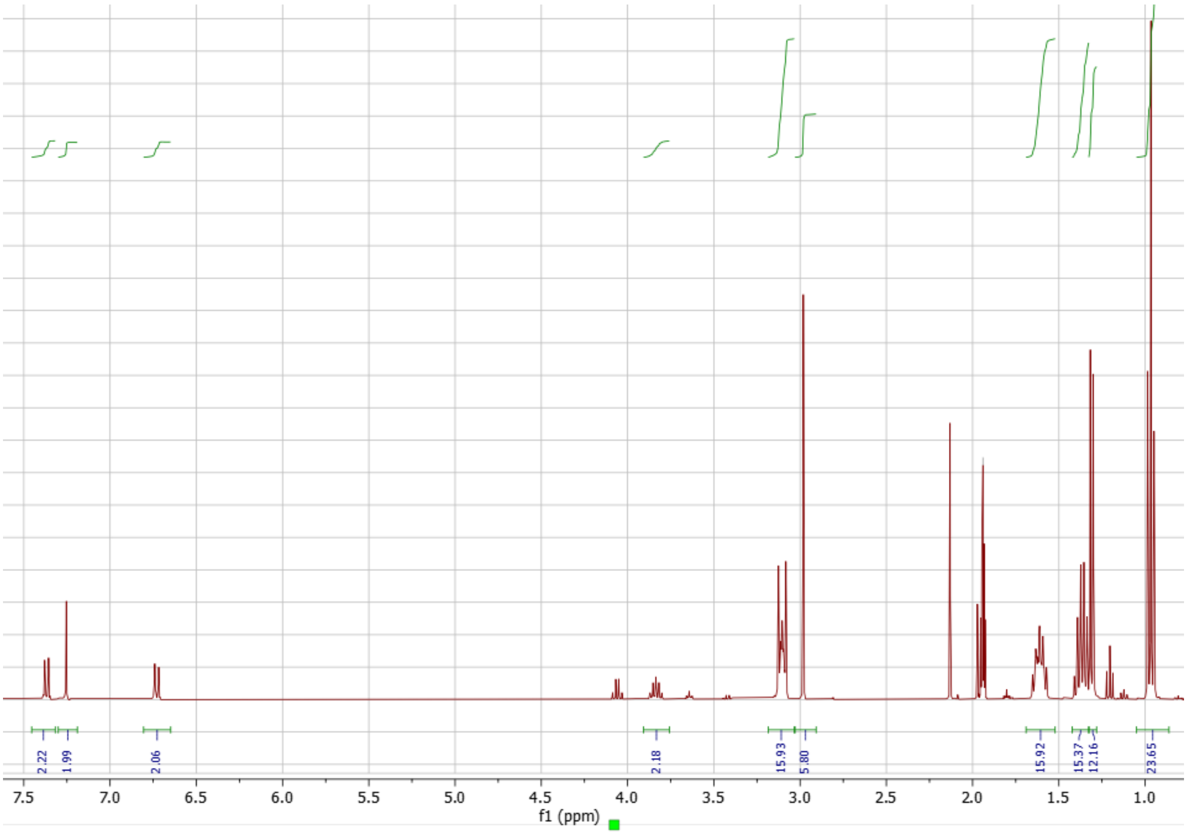

Figure S1 ^{1}H-NMR (500 MHz) spectrum of $[NBu_4]_2$[**1**] in CD_3CN. Unintegrated signals are from solvent contamination (ethyl acetate, diethyl ether, water).

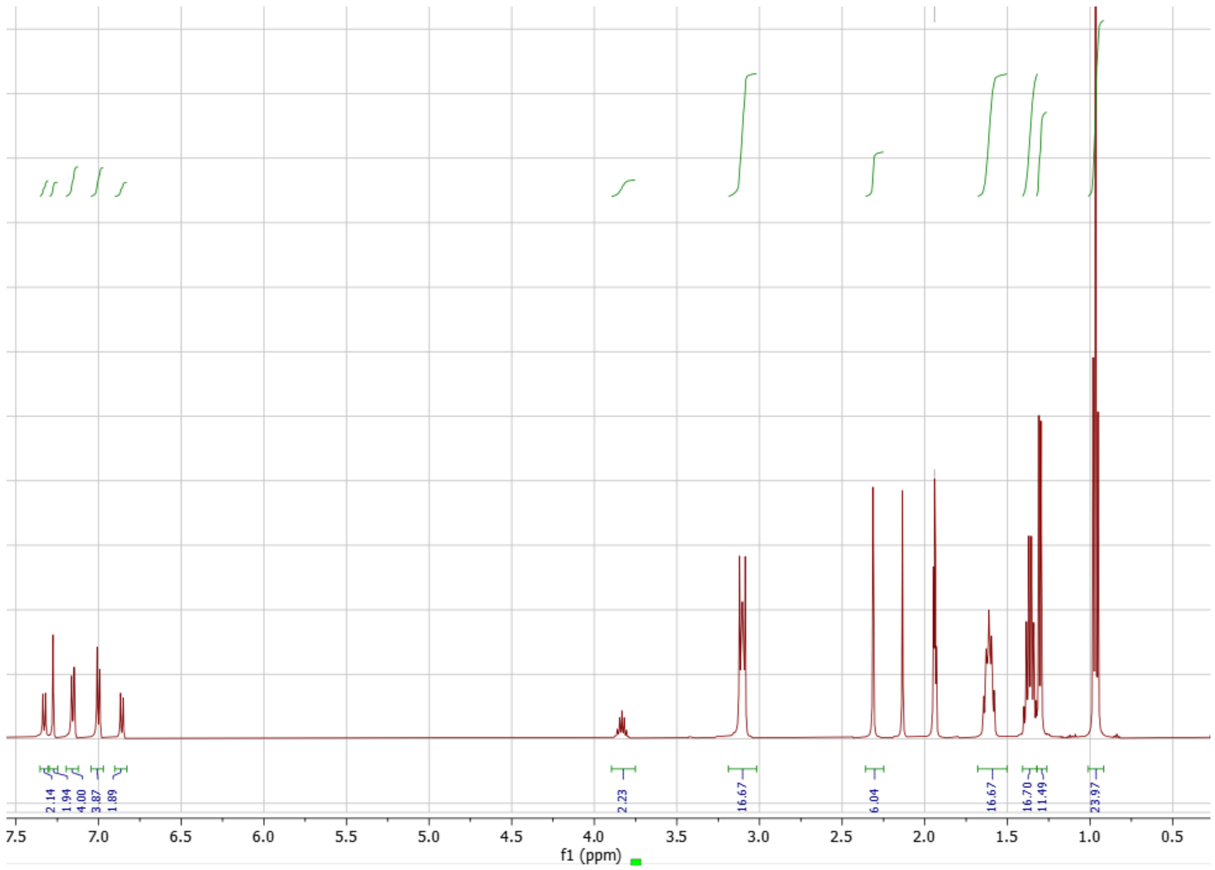

Figure S2 ^{1}H-NMR (500 MHz) spectrum of $[NBu_4]_2[\mathbf{2}]$ in CD_3CN.

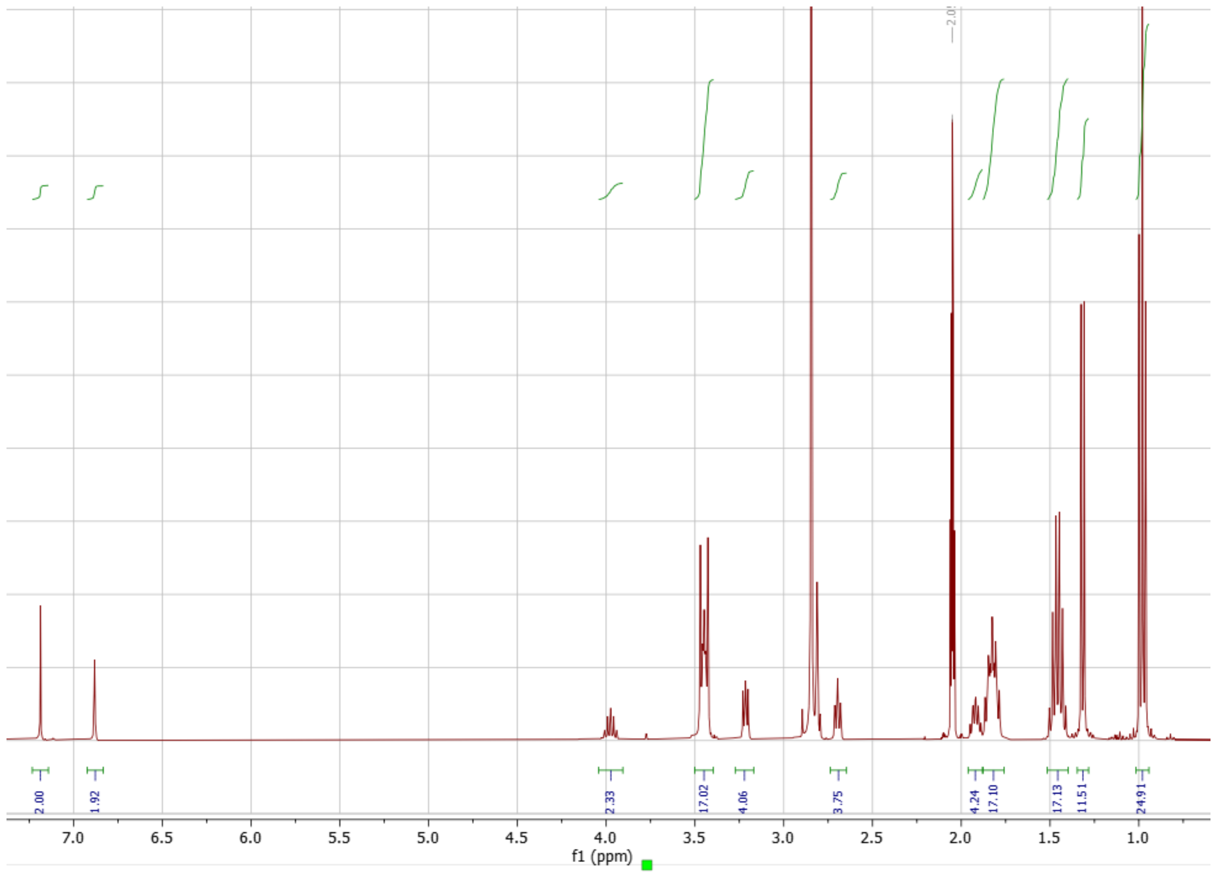

Figure S3 ^{1}H-NMR (500 MHz) spectrum of $[NBu_4]_2[\mathbf{3}]$ in CD_3CN.

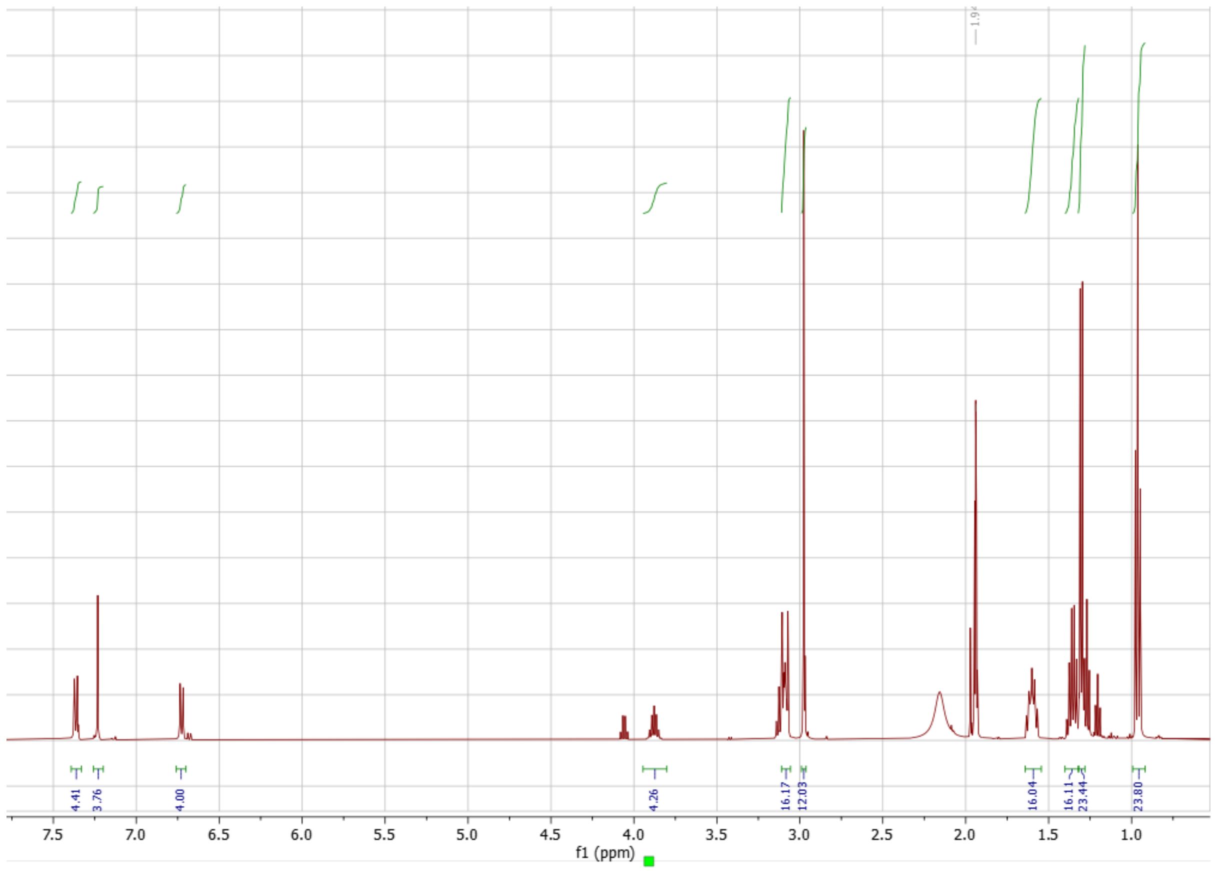

Figure S4 ^{1}H-NMR (500 MHz) spectrum of $[NBu_4]_2$[**4**] in CD_3CN.

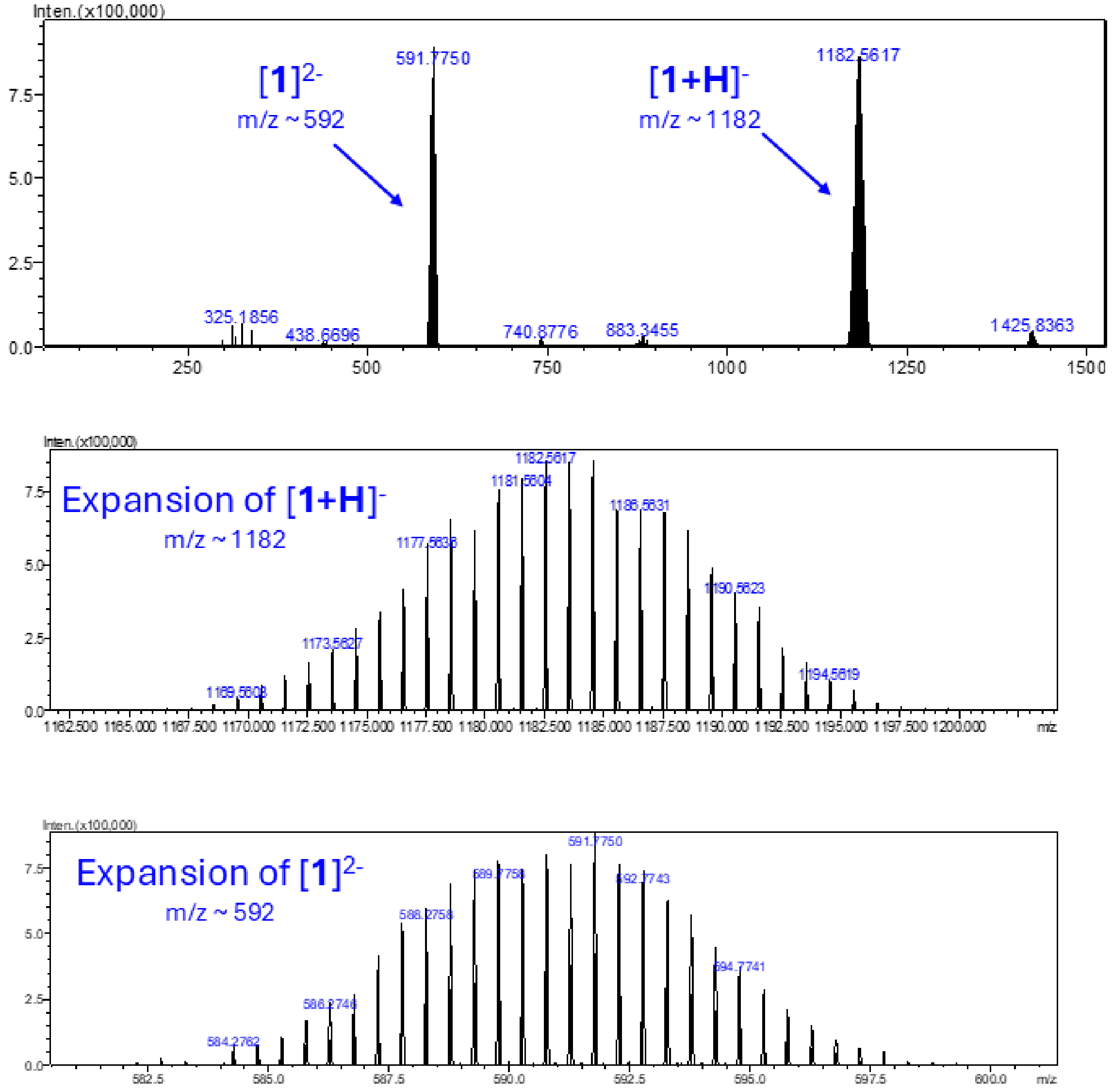

Figure S5 ESI negative mass spectrum of $[NBu_4]_2$[**1**]. Spectrum has been cut for presentation at *ca. m/z* = 1500 as there are no significant higher *m/z* signals.

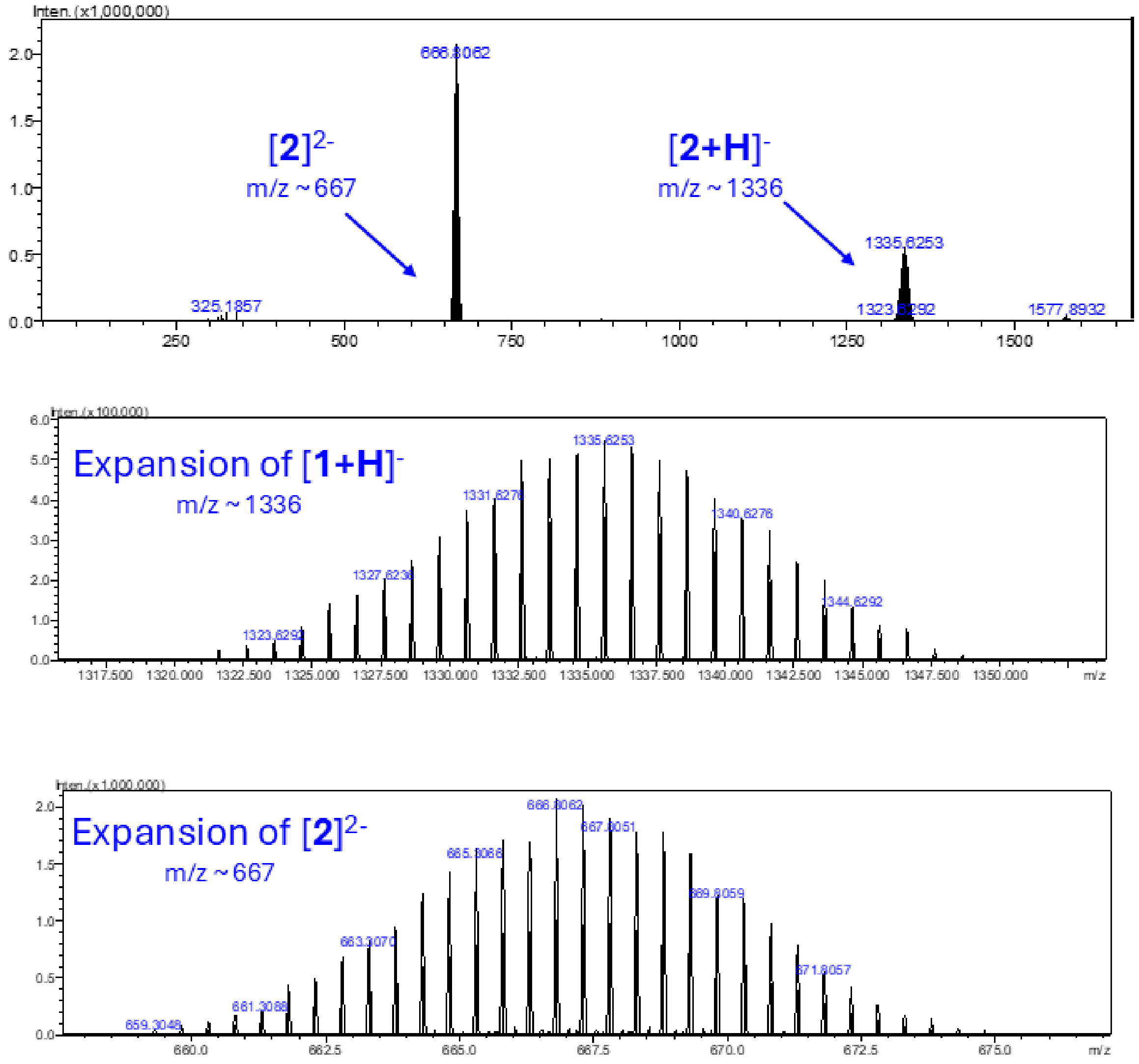

Figure S6 ESI negative mass spectrum of $[NBu_4]_2$[**2**]. Spectrum has been cut for presentation at *ca. m/z* = 1600 as there are no significant higher *m/z* signals.

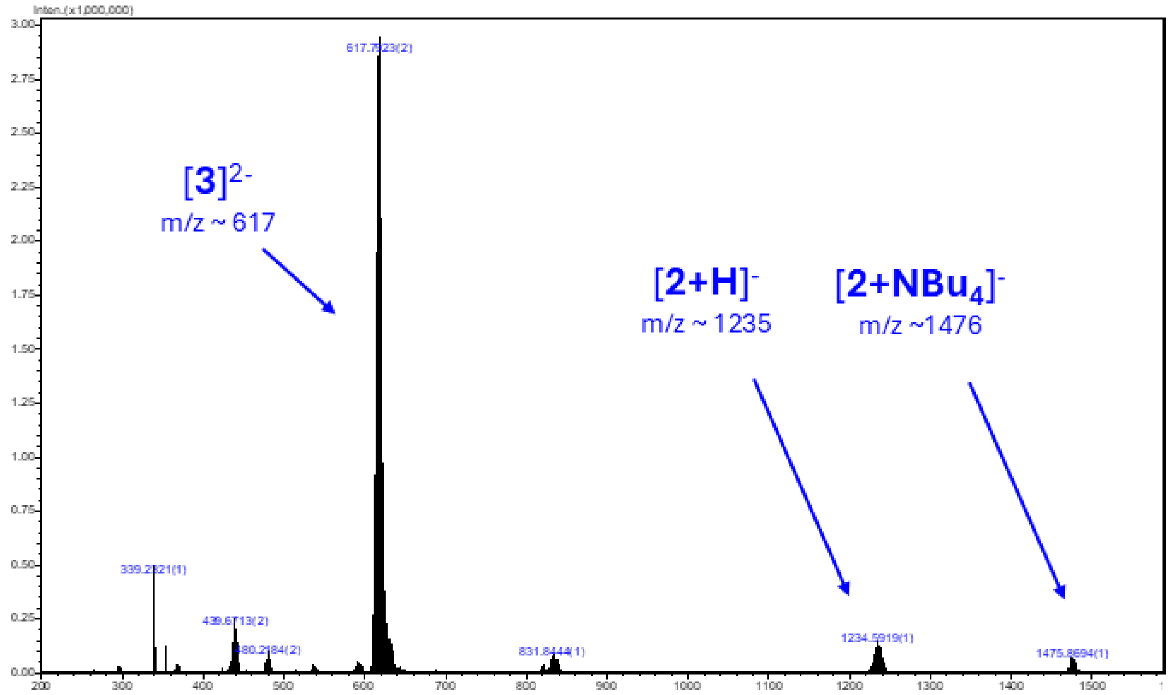

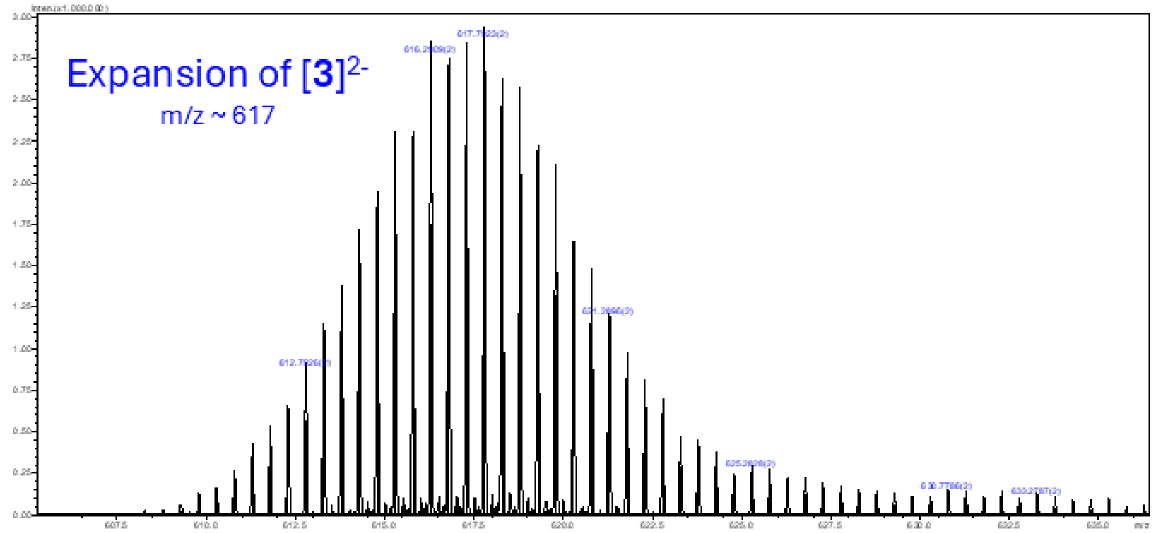

Figure S7 ESI negative mass spectrum of $[NBu_4]_2$[**3**]. Spectrum has been cut for presentation at *ca. m/z* = 1600 as there are no significant higher *m/z* signals.

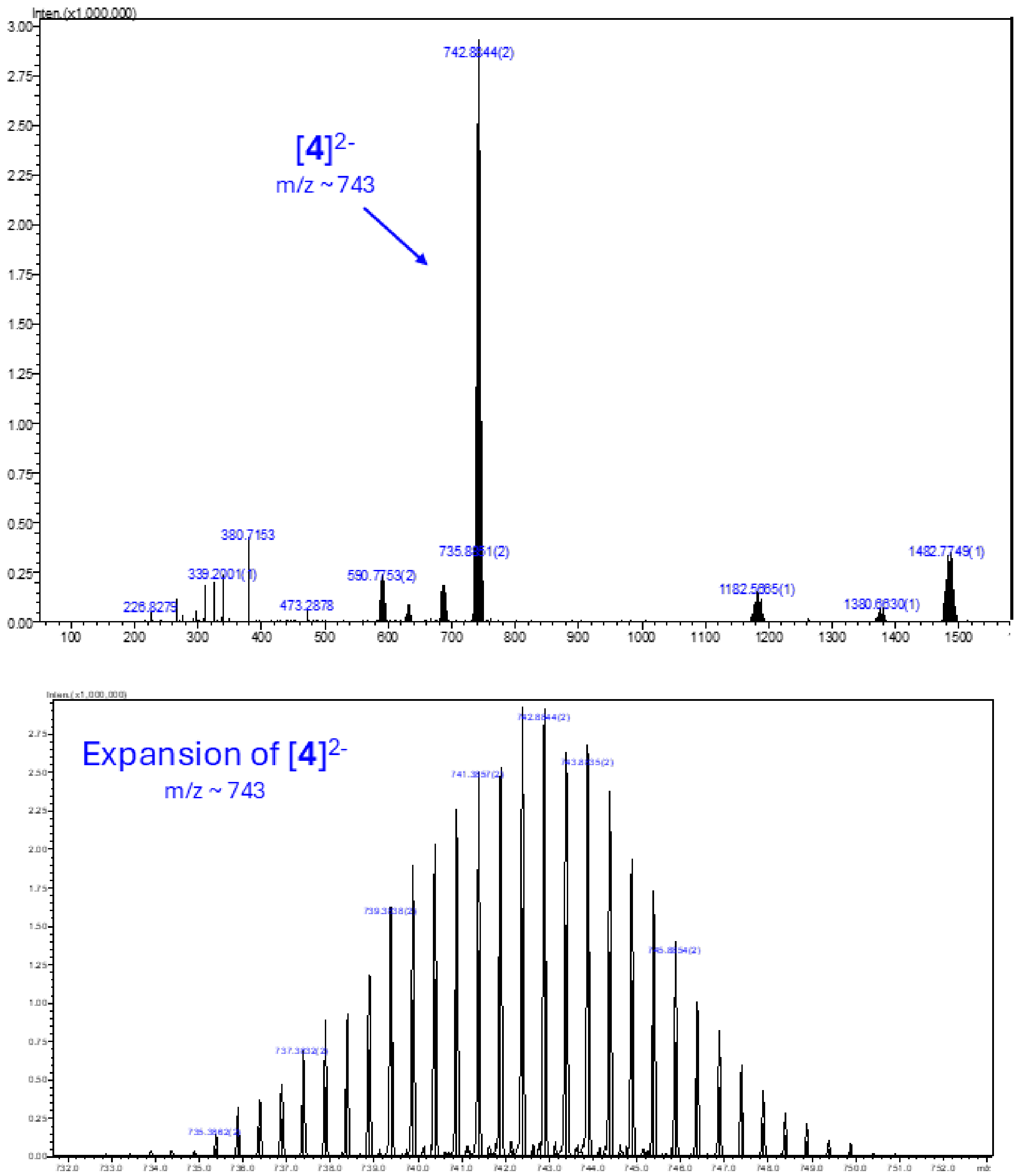

Figure S8 ESI negative mass spectrum of $[NBu_4]_2$[**4**]. Spectrum has been cut for presentation at *ca. m/z* = 1500 as there are no significant higher *m/z* signals.

2. Additional Synthetic Methods

General Methods and Materials, Synthesis of final Polyoxometalate Derivatives. These are reported in the main paper.

Precursor Synthesis. Syntheses of the final compounds $[NBu_4]_2$[**1**] to $[NBu_4]_2$[**4**] are described in the main paper. The organic precursor compounds described below: 4-iodo-2,6-diisopropylaniline (**P1**),[1] 4-bromo-N,N-di-*p*-ditolylaniline (**P4**),[1] 4-trimethylsilyethylphenyl-4,4'-ditolylamine (**P5**),[2] 4-ethylphenyl-4,4'-ditolylamine (**P6**),[3] and 4-ethynyljuloloidine

(**P8**)[4] were synthesised using or adapting known methods. These known compounds (**P1**, **P3** to **P8**) were characterized only by ^{1}H-NMR as data obtained matched that previously published. The two polyoxometalate precursor compounds $[NBu_4]_2[Mo_6O_{18}NC_{12}H_{16}I]$ ($[NBu_4]_2$[**P2**]),[5] and $[NBu_4]_2[Mo_6O_{17}(NC_{12}H_{16}I)_2]$ ($[NBu_4]_2$[**P3**])[6] were both previously published, but our methods differ in using DMSO as solvent,[7] which we have generally found to give better results than acetonitrile,[8] and using $[Mo_6O_{19}]^{2-}$ rather than $[Mo_8O_{26}]^{24-}$ as the starting material for *bis*-derivative $[NBu_4]_2$[**P3**]. Unless otherwise stated, all reactions were carried out under an atmosphere of dry argon using standard Schlenk techniques. Synthetic schemes are provided overleaf in Schemes S1 to S4.

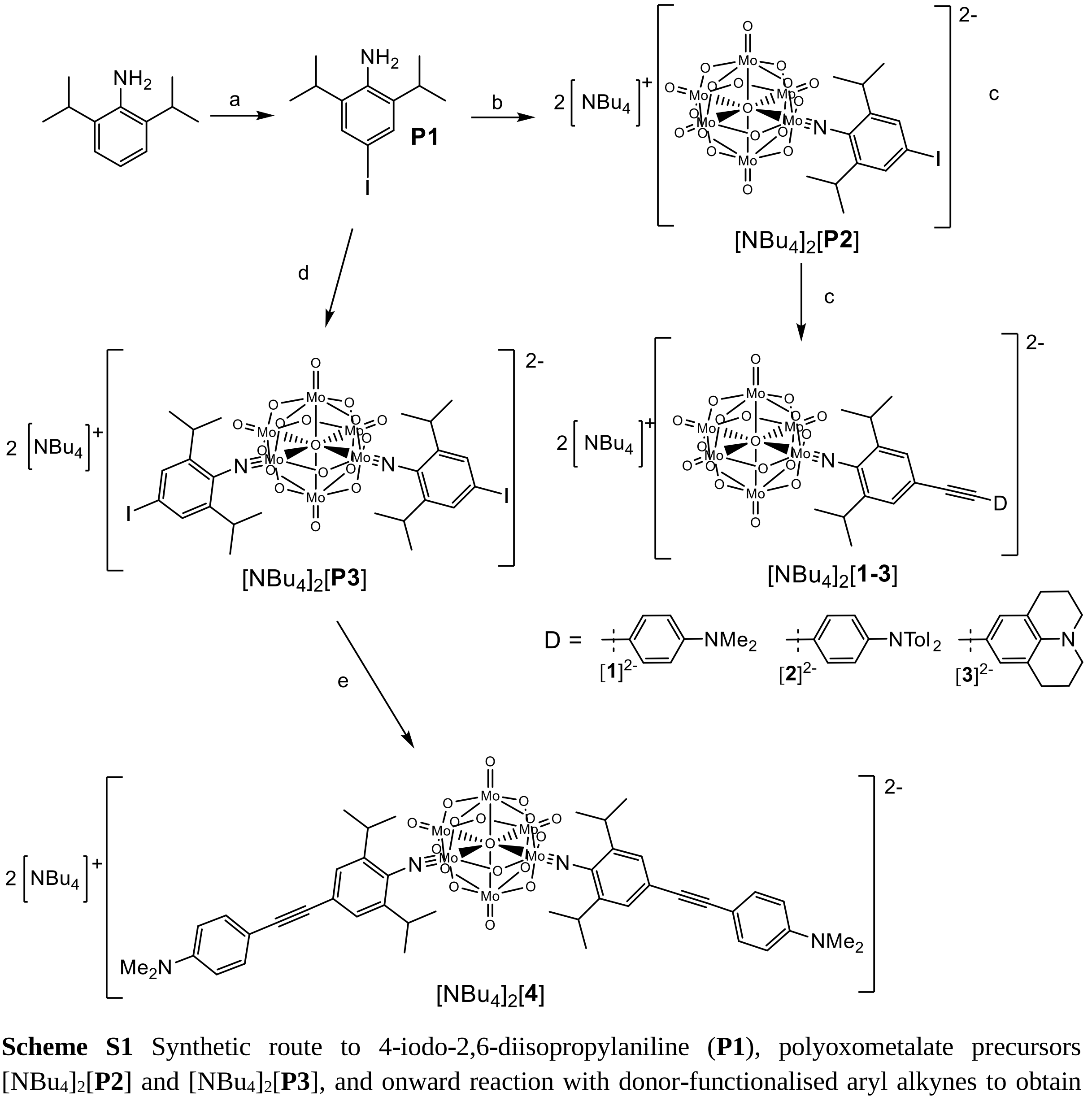

Scheme S1 Synthetic route to 4-iodo-2,6-diisopropylaniline (**P1**), polyoxometalate precursors $[NBu_4]_2$[**P2**] and $[NBu_4]_2$[**P3**], and onward reaction with donor-functionalised aryl alkynes to obtain $[NBu_4]_2$[**1**] to $[NBu_4]_2$[**4**]. Reagents, conditions and yields: (a) ICl, $NaHCO_3$, rt, 18 h, 82%. (b) $[NBu_4]_2[Mo_6O_{19}]$ (1.04 eq), DCC, DMSO, 70 °C, 10 h, 36%. (c) Alkyne (4-ethynyl-N,N-dimethylaniline, 4-ethynylphenyl-4,4'-ditolylamine (**P6**) or 4-ethynyljulolidine (**P8**)), CuI, $Pd(PPh_3)_2Cl_2$, K_2CO_3, Et_3N, MeCN, rt, 0.5 h, 47–57%. (d) $[NBu_4]_2[Mo_6O_{19}]$ (0.5 eq), DCC, DMSO, 95 °C, 24 h, 6%. (e) 4-ethynyl-N,N-dimethylaniline, CuI, $Pd(PPh_3)_2Cl_2$, K_2CO_3, Et_3N, MeCN, rt, 0.5 h, 67%.

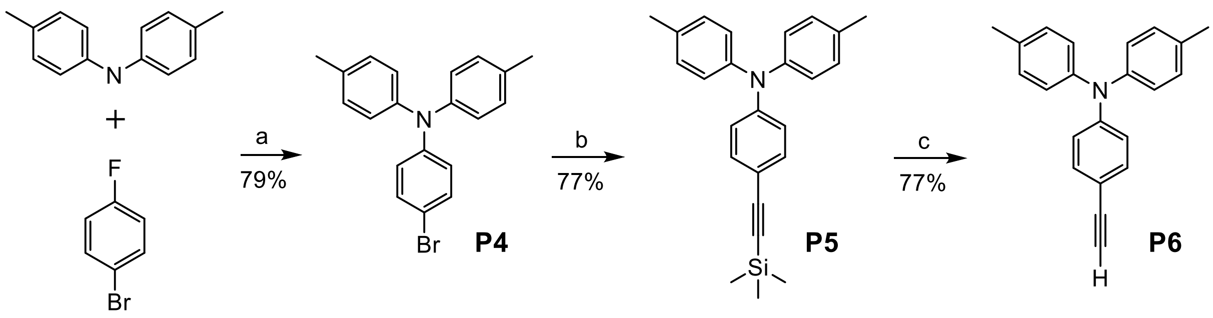

Scheme S2 Synthetic route to donor alkyne precursor **P6** used for $[NBu_4]_2$[**2**]. Reagents and conditions: (a) $Pd_2(dba)_3$, DPPF, ^tBuONa, toluene, reflux, 75 h. (b) CuI, PPh_3, $Pd(PPh_3)_2Cl_2$, TMSA, Et_3N, reflux, 20 h. (c) K_2CO_3, MeOH, THF, rt, 3h.

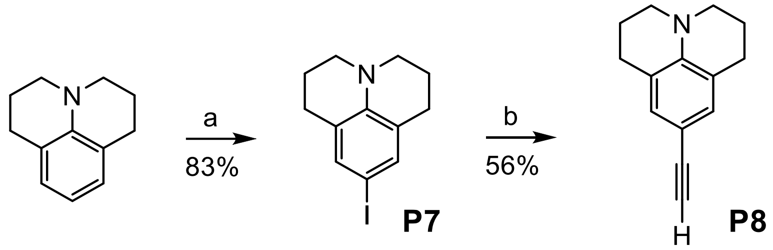

Scheme S3 Synthetic route to donor alkyne precursor **P8** used for $[NBu_4]_2$[**3**]. Reagents and conditions: (a) I_2, dioxane, Py, rt, 2.5h. (b) i: CuI, PPh_3, $Pd(PPh_3)_2Cl_2$, TMSA, Et_3N, reflux, 20h, ii: K_2CO_3, MeOH, THF, rt, 3h.

Preparation of 4-iodo-2,6-diisopropylaniline (P1). To a mixture of 2,6-diisopropylaniline (5.3 mL, 4.98 g, 28.1 mmol) and sodium bicarbonate (7.106 g, 84.6 mmol) in 40 mL of dry methanol, a solution of iodine monochloride in dichloromethane (1 M, 30 mL, 30 mmol) was added dropwise over the course of five minutes. The mixture was stirred for 18 hours at room temperature before the solids were removed by filtration to give a red solution from which the solvent was removed to give a red oil. 150 mL of saturated sodium thiosulfate solution was added and the mixture was stirred for 10 minutes before extracting with 3 × 100 mL of ethyl acetate. The organic extracts were dried over magnesium sulfate, filtered, and the solvent was removed in vacuo to give 4-iodo-2,6-diisopropylaniline as a brown oil (7.002 g, 23.1 mmol, 82%). ^{1}H-NMR (500 MHz, $CDCl_3$) δ 7.28 (s, 2H), 3.78 (s, 2H), 2.85 (hept, J = 6.3, 2H), 1.25 (d, J = 6.8 Hz, 12H). ATR: 3486 (w), 3400 (w), 2960 (s), 2928 (sh), 2870 (m), 1739 (w), 1616 (s), 1571 (m), 1459 (s), 1437 (s), 1384 (m), 1363 (sh), 1348 (s), 1299 (w) 1249 (s) 1207 (s), 1124 (m), 1098 (sh), 1062 (m), 923 (w), 887 (w), 864 (s), 832 (m), 765 (m), 715 (m) cm^{-1}. UV-vis (MeCN) λ, nm (ε, M^{-1} cm^{-1}): 210 (28.3×10^3); 252 (10.9×10^3); 296 (2.60×10^3).

Preparation of $[NBu_4]_2[Mo_6O_{18}NC_{12}H_{16}I]$ (P2). Tetrabutylammonium hexamolybdate (1.778 g, 1.32 mmol), DCC (0.310 g, 1.50 mmol), and 4-iodo-2,6-diisopropanylaniline (0.386 g, 1.27 mmol) were heated in 15 mL of dry dimethylsulfoxide at 70ºC for 10 hours. After cooling, a pale precipitate was removed by filtration and the solution precipitated by slow addition to a mixture of 200 mL of diethyl ether and 50 mL of ethanol. The resulting orange precipitate was collected and washed with ethanol and ether to give crude compound.

Purification was achieved by recrystallisation from hot acetonitrile to give $[NBu_4]_2$[**P2**] (0.751 g, 0.455 mmol) as dark orange crystals with a 36% yield. ^{1}H-NMR (500 MHz, CD_3CN): δ 7.51 (s, 2H), 3.78 (hept, *J* = 6.8 Hz, 2H), 3.26 – 2.95 (m, 16 H), 1.65 – 1.55 (m, 16H), 1.36 (*a*sex, *J* = 7.0 Hz, 16H), 1.27 (d, *J* = 6.8 Hz, 12H), 0.97 (t, *J* = 7.5 Hz, 24H). ^{13}C-NMR (126 MHz, CD_3CN): δ 151.26, 132.51, 95.28, 59.29, 29.44, 24.32, 23.86, 20.30, 13.80. Anal (Calcd) % for $C_{44}H_{88}N_3O_{18}Mo_6I$: C 32.02 (32.18), H 5.38 (5.48), N 2.55 (2.60). HRMS (ESI, MeCN) = calcd for $C_{12}H_{16}NO_{18}Mo_6I^{2-}$ 581.6888, found 581.6875. ATR: 2960 (m), 2933 (sh), 2873 (m), 1560 (w), 1479 (m), 1461 (w), 1418 (m), 1380 (m), 1363 (sh), 1337 (m), 1168 (w), 1148 (w), 1107 (w), 1068 (w), 1028 (w), 974 (m), 943 (s), 882 (w), 855 (sh), 769 (w). UV-vis (MeCN) λ, nm (ε, M^{-1} cm^{-1}): 204 (47.9×10^3); 247 (33.3×10^3); 364 (23.5×10^3).

Preparation of $[NBu_4]_2[Mo_6O_{17}(NC_{12}H_{16}I)_2]$ ($[NBu_4]_2$[P3]). To tetrabutylammonium hexamolybdate (1.999 g, 1.46 mmol) and DCC (0.756 g, 3.68 mmol) dissolved in 15 mL of dry dimethylsulfoxide, 4-iodo-2,6-diisopropanylaniline (**P1**) (0.887 g, 2.93 mmol) was added. The resulting brown solution was heated at 95 ºC for 24 hours. When cool, a pale yellow precipitate was removed by filtration and the solution precipitated by slow addition to a mixture of 400 mL of diethyl ether and 80 mL of ethanol. The yellow precipitate was removed, and more diethyl ether added until an orange precipitate formed. This was collected by filtration, washed with ethanol and diethyl ether, and then purified by recrystallisation from hot acetonitrile to yield $[NBu_4]_2$[**P3**] (0.160 g, 0.083 mmol, 6%) as dark orange crystals. ^{1}H-NMR (500 MHz, CD_3CN): δ 7.47 (s, 2H), 3.82 (hept, *J* = 6.8 Hz, 2H), 3.13 – 3.06 (m, 16H), 1.60 (tt, *J* = 8.2, 6.2 Hz, 16H), 1.36 (*a*sex, *J* = 7.4 Hz, 16H), 1.26 (d, *J* = 6.9 Hz, 12H), 0.96 (t, *J* = 7.4 Hz, 24H). ^{13}C-NMR (126 MHz, CD_3CN) δ 150.63, 132.38, 94.01, 59.28, 29.31, 24.29, 23.94, 20.31, 20.29, 13.77. Anal (Calcd) % for $C_{56}H_{104}N_4O_{17}Mo_6I_2$: C, 34.76 (34.60); H, 5.42 (5.54); N, 2.90 (2.99). HRMS (ESI, MeCN) = calcd for $C_{24}H_{32}N_2Mo_6O_{17}I^{2-}$ 725.7080, found 725.7063. FTIR: 2959 (m), 2931 (sh), 2872 (m), 1560 (m), 1476 (m), 1417 (sh), 1381 (m), 1361 (w), 1336 (m), 1309 (w), 1293 (s) 1229 (sh), 1169 (m), 1146 (sh), 1108 (w), 1069 (w), 1030 (w), 965 (s), 937 (vs), 882 (m), 863 (m), 759 (vs). UV-vis (MeCN) λ, nm (ε, M^{-1} cm^{-1}): 206 (82.8×10^3); 252 (61.0×10^3); 366 (45.3×10^3).

Preparation of 4-bromo-N,N-di-*p*-ditolylaniline (P4). Di-*p*-tolylamine (4.761 g, 24.1 mmol), 1-bromo-4-iodobenzene (7.520 g, 26.6 mmol), tris(dibenzylideneacetone) dipalladium(0) (0.221 g, 0.241 mmol), 1,1'- bis(diphenylphosphino) ferrocene (0.405 g, 0.731 mmol), and sodium *tert*-butoxide (3.48 g, 36.2 mmol) were dissolved in toluene (15 mL). The resulting red solution was refluxed for 75 hours and filtered once cool to produce a brown solution. The solvent was removed *in vacuo* to produce a brown oil which after purification by column chromatography in hexane:dichloromethane 97:3 yielded compound **P4** as an off white solid (6.723 g, 19.1 mmol, 79%). ^{1}H-NMR (500 MHz, $CDCl_3$) δ 7.27 (d, *J* = 8.9 Hz, 2H), 7.06 (d, *J* = 8.4 Hz, 4H), 6.97 (d, *J* = 8.4 Hz, 4H), 6.89 (d, *J* = 8.8 Hz, 2H), 2.31 (s, 6H).

Preparation of 4-trimethylsilyethylphenyl-4,4'-ditolylamine (P5). Compound **P4** (3.007 g, 8.54 mmol), copper iodide (0.157 g, 0.824 mmol), triphenylphosphine (0.232 g, 0.885 mmol), bis(triphenylphosphine) palladium(II) dichloride (0.171 g, 0.244 mmol), and trimethylsilylacetylene (1.8 ml, 1.242 g, 12.6 mmol) were dissolved in triethylamine (30 mL). The resulting yellow solution was refluxed for 20 hours and filtered before stirring with satd.

aqueous NH_4Cl (40 mL) and extraction with 2 × 40 mL ethyl acetate. The organic layers were dried over $MgSO_4$ and then the solvent was removed *in vacuo* to give **P5** as a yellow oil (2.416 g, 6.54 mmol, 77% yield). ^{1}H-NMR (500 MHz, $CDCl_3$) δ 7.27 (d, *J* = 8.8 Hz, 2H), 7.07 (d, *J* = 8.2 Hz, 4H), 6.98 (d, *J* = 8.4 Hz, 4H), 6.88 (d, *J* = 8.8 Hz, 2H), 2.31 (s, 6H), 0.23 (s, 9H).

Preparation of 4-ethynylphenyl-4,4'-ditolylamine (P6). To compound **P5** (2.416 g, 6.54 mmol) in methanol (6 mL) and tetrahydrofuran (6 mL), K_2CO_3 (1.826 g, 13.2 mmol) was added and resulting mixture was stirred for 3 hours. After filtering, the resulting yellow solution was stirred with satd. aqueous NH_4Cl (40 mL) before extracting into 2 × 40 mL of ethyl acetate. The organic layers were washed with brine and dried over $MgSO_4$ before the solvent was removed *in vacuo* to give compound **P6** (1.490 g, 5.01 mmol, 77%) as a yellow solid. ^{1}H-NMR (500 MHz, $CDCl_3$) δ 7.30 – 7.27 (m, 2H), 7.08 (d, *J* = 8.1 Hz, 4H), 7.02 – 6.98 (m, 4H), 6.92 – 6.88 (m, 2H), 3.00 (s, 1H), 2.32 (s, *J* = 6.3 Hz, 6H). ATR: 3262 (s), 3032 (w), 2919.21 (w), 2858 (m), 2102 (m), 2009 (m), 1643 (s), 1498 (s), 1317 (s), 1293 (s), 1270 (sh) 1174 (m), 1109 (m), 1019 (w) 833 (sh), 813 (s) cm^{-1}.

Preparation of 4-iodojulolidine (P7). To a solution of julolidine (2.500 g, 14.4 mmol) in dioxane (60 mL) and pyridine (75 mL) at 0°C, I_2 (11 g, 43.3 mmol) was added. After stirring at room temperature for 2.5 h, the reaction was quenched with satd. aqueous sodium thiosulfate. Extracting into dichloromethane, washing with water, then drying with $MgSO_4$ and removing the solvent under vacuum gave compound **P7** (3.588 g, 12.0 mmol, 83%) as a dark orange oil. ^{1}H-NMR (500 MHz, $CDCl_3$) δ 7.05 (t, *J* = 0.9 Hz, 2H), 3.19 – 3.04 (m, 4H), 2.69 (t, *J* = 6.5 Hz, 4H), 2.00 – 1.87 (m, 4H).

Preparation of 4-ethynyljulolidine (P8). To a mixture of **P7** (3.203 g, 10.7 mmol), bis(triphenylphosphine) palladium(II) dichloride (0.209 g, 0.30 mmol), and copper iodide (0.099 g, 0.90 mmol) in diisopropylamine (100 mL) ethynyltrimethylsilane (1.8 mL, 1.24 g, 13.8 mmol) was added. The resulting mixture was stirred at room temperature for 3 days before addition to 125 mL of hexane and filtering through celite. The solvent was removed to give a dark oil which was dissolved in a mixture of methanol (25 mL) and tetrahydrofuran (25 mL). Potassium carbonate (4.503 g, 32.5 mmol) was added, and the mixture stirred for 3 hours before filtering and removing the solvent under vacuum. Purification by column chromatography using DCM eluent gave compound **P8** (1.186 g, 6.01 mmol, 56%) as a yellow oil. ^{1}H-NMR (500 MHz, $CDCl_3$) δ 6.93 (t, *J* = 0.8 Hz, 2H), 3.17 (t, *J* = 5.6 Hz, 4H), 2.91 (s, 1H), 2.70 (t, *J* = 6.4 Hz, 4H), 1.96 – 1.91 (m, 4H).

3. X-ray Crystallographic Details

Sample Growth, Data Collection and Refinement. Crystals of $[NBu_4]_2$[**1**], $[NBu_4]_2$[**2**]•0.25MeCN•0.25Et_2O, $[NBu_4]_2$[**3**] and $[NBu_4]_2$[**4**] were grown by diffusion of diethyl ether into acetonitrile solutions. Crystals of $[NBu_4]_2$[**P2**]•Et_2O were grown by diffusion of diethyl ether into an acetone solution. Data were collected on a Rigaku XtalLab Synergy S diffractometer using a Photon-Jet Cu or Mo microfocus source and Hypix hybrid photon counting detector. Data reduction, cell refinement and absorption correction were carried out

using Rigaku CrysAlisPro,[9] and the structure was solved with SHELXT[10] in Olex2 V1.5.[11] Refinement was achieved by full-matrix least-squares on all F_0^2 data using SHELXL (v. 2018-3),[12] also in Olex 2 V1.5. The asymmetric units (Figures S9 to S13) contain the complete molecular anion for $[NBu_4]_2$[**1**], $[NBu_4]_2$[**3**] and $[NBu_4]_2$[**4**], with two crystallographically independent $[NBu_4]^+$, while the asymmetric unit for $[NBu_4]_2$[**2**] •0.25MeCN•0.25Et_2O contains two crystallographically independent molecular anions, plus four $[NBu_4]^+$ and 50% occupied Et_2O and MeCN molecules. The asymmetric unit of precursor $[NBu_4]_2$[**P2**] also contains two crystallographically independent molecular anions and four $[NBu_4]^+$, plus two Et_2O. Disorder in tetrabutylammonium cations in $[NBu_4]_2$[P**2**], $[NBu_4]_2$[**2**] and $[NBu_4]_2$[**3**], and in the julolidinyl group of $[NBu_4]_2$[**3**], required application of restraints to thermal parameters and some distances, restraints were also applied to some thermal parameters in $[NBu_4]_2$[**4**]. $[NBu_4]_2$[**2**] required use of a mask (SQUEEZE) due to solvent electron density peaks that would not refine, void space was located in $[NBu_4]_2$[**1**] and $[NBu_4]_2$[**4**], this contained minimal electron density and may be responsible for the low stability and difficult growth of these crystals.

X-ray crystallographic data and refinement parameters are summarised overleaf in Table S1.

Table S1. Crystallographic Data and Refinement Details for $[NBu_4]_2$[**1**] and $[NBu_4]_2$[**2**]

	$[NBu_4]_2$[**P2**]•Et_2O	$[NBu_4]_2$[**1**]	$[NBu_4]_2$[**2**]•0.25MeCN •0.25Et_2O	$[NBu_4]_2$[**3**]	$[NBu_4]_2$[**4**]
Formula	$C_{47}H_{94}IMo_6N_4O_{19}$	$C_{54}H_{98}Mo_6N_4O_{18}$	$C_{67.5}H_{109.25}Mo_6N_{4.25}O_{18.25}$	$C_{58}H_{102}Mo_6N_4O_{18}$	$C_{76}H_{124}Mo_6N_6O_{17}$
M	1707.79	1667.00	1847.97	1719.07	1969.44
crystal system	Monoclinic	Tetragonal	Monoclinic	Triclinic	Tetragonal
space group	*P2₁/c*	*I-4*	*P2₁/n*	*P-1*	*I-4*
a/Å	24.1429(2)	34.6856(4)	39.5053(4)	11.7095(2)	36.1329(5)
b/Å	12.2225(1)	34.6856(4)	11.9905(1)	12.0820(2)	36.1329(5)
c/Å	43.8261(4)	11.9055(3)	40.8345(4)	25.6940(6)	13.4562(3)
α/deg	90	90	90	94.122(2)	90
β/deg	94.254(1)	90	107.896(1)	98.862(2)	90
γ/deg	90	90	90	94.302(2)	90
V/Å^3	12896.9(2)	14323.4(5)	18406.9(3)	3568.7(1)	17568.2(6)
Z	8	8	8	2	8
Radiation	Mo Kα (λ = 0.71073 Å)	Cu Kα (λ = 1.54184 Å)	Mo Kα (λ = 0.71073 Å)	Mo Kα (λ = 0.71073 Å)	Cu Kα (λ = 1.54184 Å)
T/K	100.0(1)	100.00(1)	100.0(1)	100.0(1)	100.0(1)
μ/mm^{-1}	1.675	8.863	0.848	1.087	7.305
Cryst. size/mm	0.12 × 0.1 × 0.08	0.66 × 0.30 × 0.04	0.33 × 0.25 × 0.21	0.12 × 0.1 × 0.08	0.54 × 0.08 × 0.06
Cryst. description	Light orange needle	Dark orange plate	Orange block	Red plate	Dark orange needle
No. reflns collected	425344	34698	560609	98520	37878
No. of indep. reflns [R_{int}]	36589 [R_{int} = 0.0590]	13401 [R_{int} = 0.0566]	53063 [R_{int} = 0.0598]	19194 [R_{int} = 0.0702]	14546 [R_{int} = 0.0568]
2θ_{full}/deg<br>2θ_{max}/deg	50.484<br>61.976	135.368<br>154.274	50.484<br>62.198	50.484<br>61.782	134.158<br>134.158
Completeness at θ_{full} (Laue)	100%	99.6%	99.9%	99.9%	98.7%
Completeness at θ_{full} (point group)	100%	93.7%	99.9%	99.9%	92.7%
Reflections with $I > 2\sigma(I)$	32236	11380	44307	13542	12058
Goodness-of-fit on F^2	1.105	1.092	1.140	1.013	1.021
final R_1, wR_2 [$I > 2\sigma(I)$]^a	R_1 = 0.0348, wR_2 = 0.0721	R_1 = 0.0573, wR_2 = 0.1567	R_1 = 0.0607, wR_2 = 0.1219	R_1 = 0.0628, wR_2 = 0.1545	R_1 = 0.0668, wR_2 = 0.1736
(all data)	R_1 = 0.0422, wR_2 = 0.0740	R_1 = 0.0715, wR_2 = 0.1768	R_1 = 0.0739, wR_2 = 0.1256	R_1 = 0.0900, wR_2 = 0.1665	R_1 = 0.0794, wR_2 = 0.1834
Peak and hole/e Å^{-3}	2.02/-1/52	0.89/-1.27	2.21/-1.70	1.83/-1.15	2.47/-0.56
Flack parameter	N/A	0.001(10)	N/A	N/A	0.022(10)

Structures and Selected Bond Lengths. The contents of the asymmetric units of $[NBu_4]_2$[**P2**] and $[NBu_4]_2$[**1**] to $[NBu_4]_2$[**4**] are illustrated below in Figures S9 to S13.

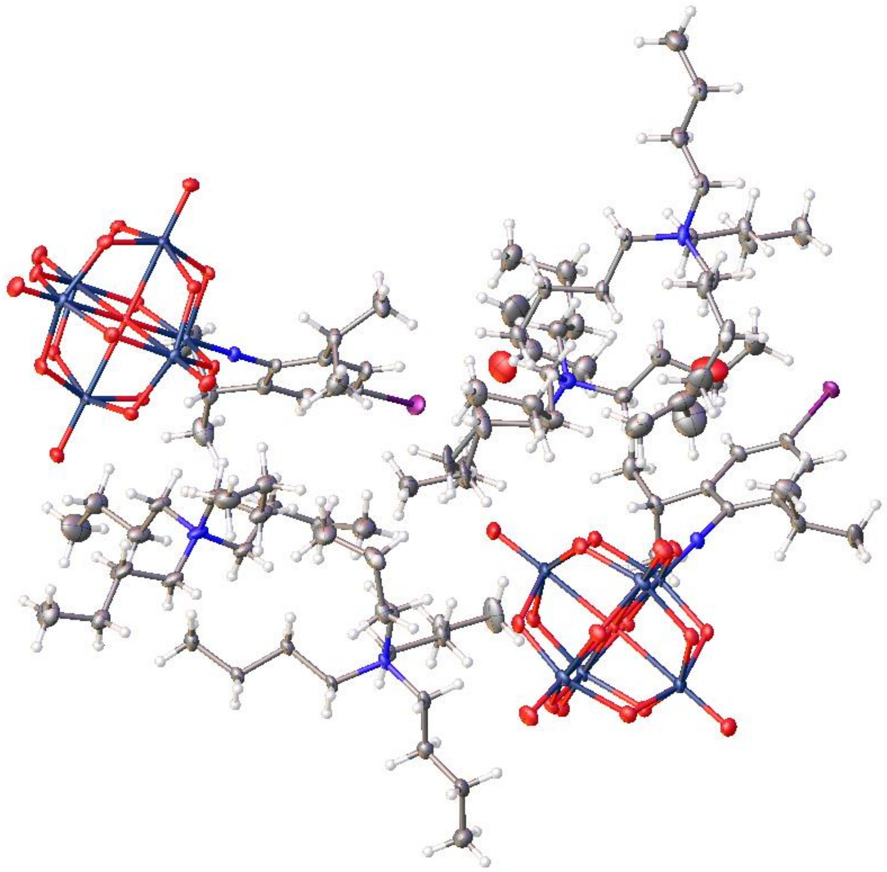

Figure S9 ORTEP representation of the asymmetric unit in $[NBu_4]_2$[**P2**]. Thermal ellipsoids are at the 30% probability level. Colour scheme: Mo is navy; O, red; C, gray; N, blue; I, purple; H atoms are represented by white spheres of arbitrary radii.

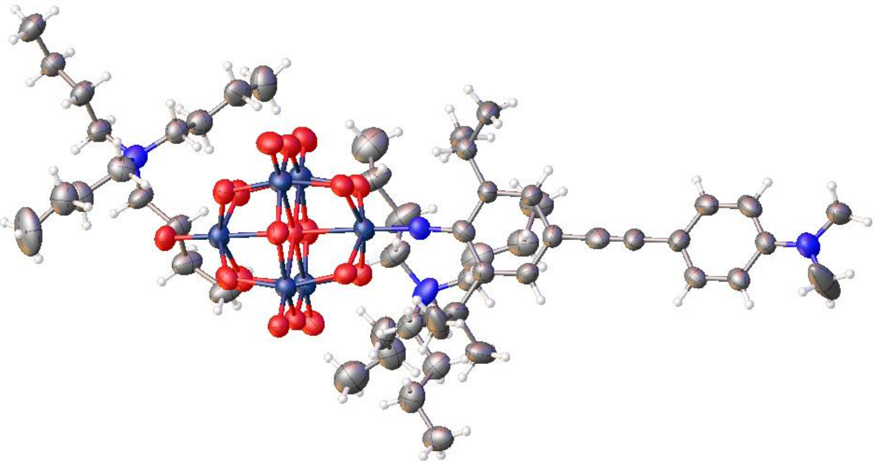

Figure S10 ORTEP representation of the asymmetric unit in $[NBu_4]_2$[**1**]. Thermal ellipsoids are at the 30% probability level. Colour scheme: Mo is navy; O, red; C, gray; N, blue; H atoms are represented by white spheres of arbitrary radii.

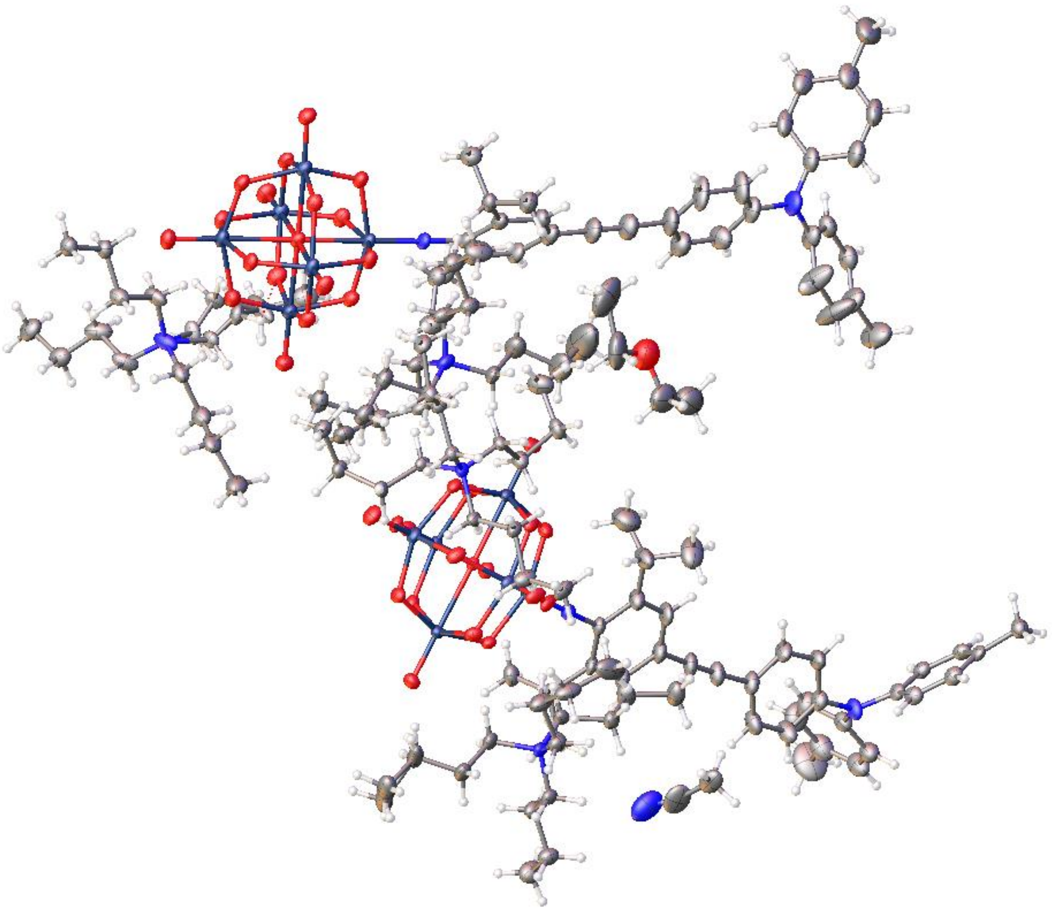

Figure S11 ORTEP representation of the asymmetric unit in $[NBu_4]_2$[**2**]. Thermal ellipsoids are at the 30% probability level and disordered parts are omitted for clarity. Colour scheme: Mo is navy; O, red; C, grey; N, blue; H atoms are represented by white spheres of arbitrary radii.

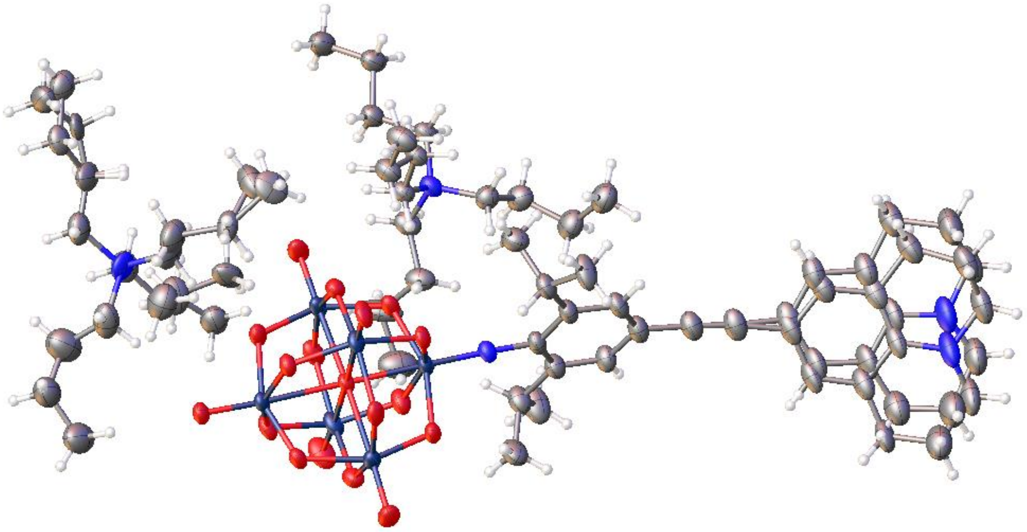

Figure S12 ORTEP representation of the asymmetric unit in $[NBu_4]_2$[**3**]. Thermal ellipsoids are at the 30% probability level. Colour scheme: Mo is navy; O, red; C, grey; N, blue; H atoms are represented by white spheres of arbitrary radii.

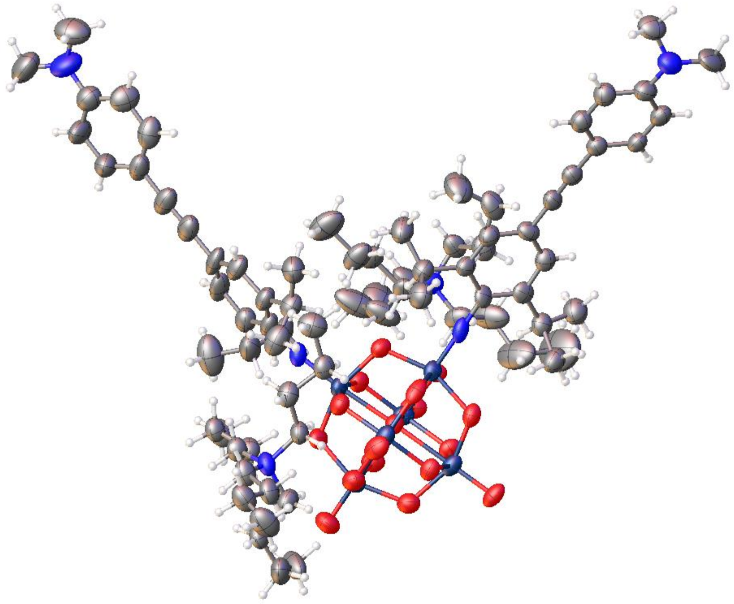

Figure S13 ORTEP representation of the asymmetric unit in $[NBu_4]_2$[**4**]. Thermal ellipsoids are at the 30% probability level. Colour scheme: Mo is navy; O, red; C, grey; N, blue; H atoms are represented by white spheres of arbitrary radii.

Selected bond lengths and angles for the anions are collected in Table S2. The structures show the typical imido-Lindqvist pattern of a shortened bond length from the imido-Mo (Mo^{im}) to the central oxygen (O^c), lengthened equatorial bond lengths from Mo^{im} to the oxygens bridging to the belt Mo positions (Mo^b), and a lengthened axial bond length from the *trans*-Mo (Mo^t) to O^c.[5-8,13] Low bond length precision in $[NBu_4]_2$[**1**] and $[NBu_4]_2$[**4**] prevents meaningful discussion of bond distances within the π-bridges and donor groups, but generally, these seem to be consistent with those previously observed for diphenylacetylene POM imido derivatives.[8]

Table S2. Selected bond lengths (Å) and angles (°) of [**P2**]$^{2-}$ and [**1**]$^{2-}$ to [**4**]$^{2-}$.

	Mo^{im}-N^{im}	Mo^{im}-O^b (av)	Mo^t-O^b (av)	N^{im}-C	Mo^{im}-O^c	Mo^t-O^c	Mo^{im}-N-C	Alkyne Twist Angle
[**P2**]$^{2-\ a}$	1.741(2)	1.944(2)	1.921(2)	1.381(3)	2.198(2)	2.369(2)	177.2(2)	N/A
[**1**]$^{2-}$	1.74(1)	1.97(1)	1.92(1)	1.38(2)	2.205(8)	2.363(8)	176(1)	17.6
[**2**]$^{2-\ a}$	1.738(3)	1.953(3)	1.920(3)	1.390(5)	2.201(3)	2.361(3)	174.2(3)	11.8
[**3**]$^{2-}$	1.736(4)	1.949(4)	1.913(4)	1.382(6)	2.203(3)	2.350(3)	177.7(4)	44.5
[**4**]$^{2-}$	1.72(1)^b	1.95(1)	1.92(1)	1.40(2)^b	2.232(9)^b	2.380(9)^b	175(1)^b	27.6 / 10.3^c

$^{\boldsymbol{a}}$Values reported are averages of two crystallographically independent anions. ^{b}Average of the two Mo imido groups. ^{c}Values reported independently due to large difference in twist angle. The mean – 19° is very similar to that observed in *mono* analogue [**1**]$^{2-}$. Esds on mean values are simple averages of the esds of contributing distances. They indicate the average esd on a contributing bond length, not the standard deviation of the mean. Mo^{im} is the imido carrying Mo atom, Mo^t the Mo *trans* to the imido (across the central oxygen), O^c the central oxygen, O^b the oxygens bridging to belt Mo positions to which distances are necessarily averaged. Mean terminal Mo=O distances in all structures range from 1.68(1) to 1.70(1) Å, these distances show no pattern either within or between structures.

4. Cyclic Voltammograms

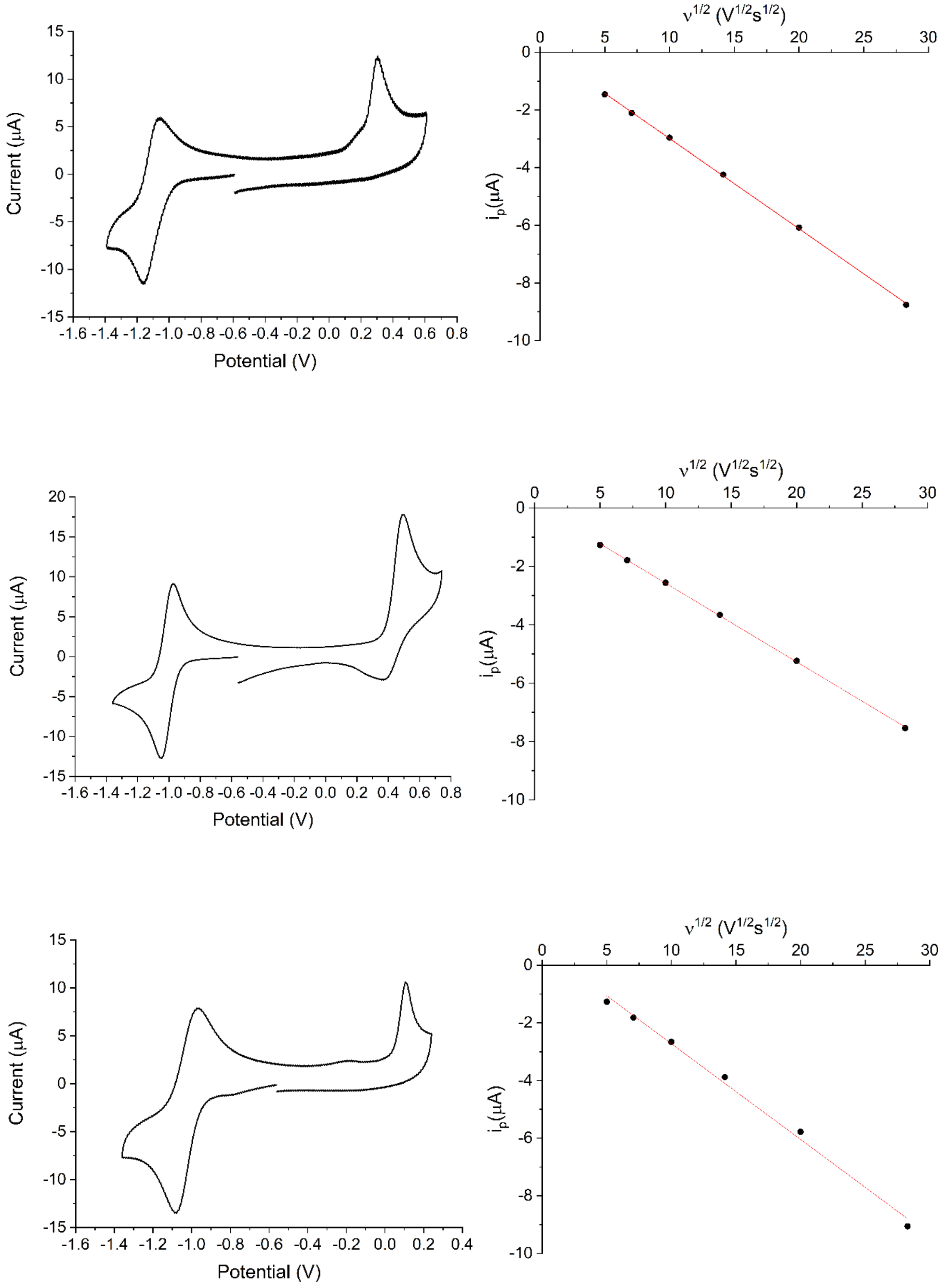

Figure S14 Left: Cyclic voltammograms of $[NBu_4]_2$[**1**] (top), $[NBu_4]_2$[**2**] (middle) and $[NBu_4]_2$[**3**] (bottom) showing amine donor oxidation peaks at *ca* 0.4 V and the reversible POM reduction peak at *ca.* -1V (both *vs* Fc/Fc^+). Scan rate 100 mV s^{-1}, GC working electrode, 0.1 M $[NBu_4][BF_4]$ in acetonitrile as electrolyte. **Right:** Peak current *vs* square root of scan rate for the first POM reduction of $[NBu_4]_2$[**1**] (top), $[NBu_4]_2$[**2**] (middle) and $[NBu_4]_2$[**3**] (bottom).

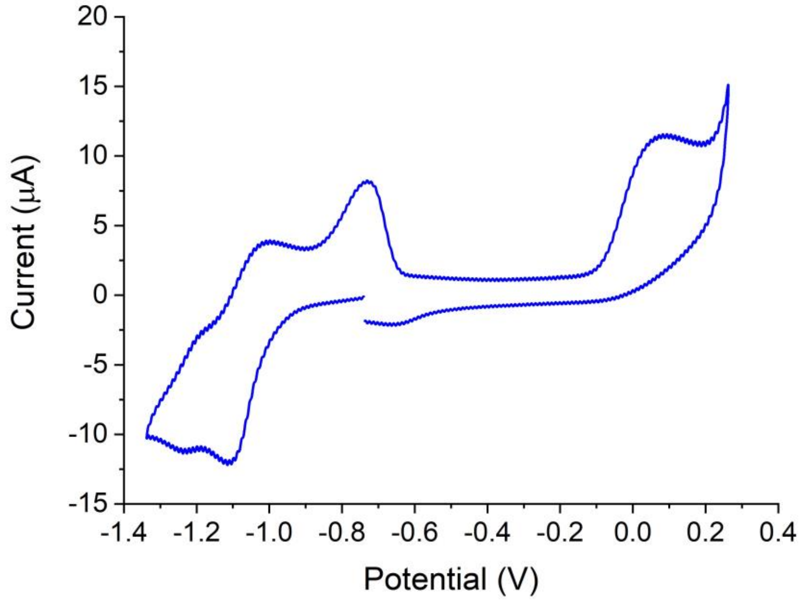

Figure S15: Full range cyclic voltammogram of $[NBu_4]_2$[**4**] showing the irreversible amine donor oxidation peak at *ca* 0.15, quasi-reversible POM reduction peak at *ca.* -1.1 V, and a reoxidation peak of the destroyed material at -0.8 V (all *vs* Fc/Fc^+). Scan rate 100 mV s^{-1}, GC working electrode, 0.1 M $[NBu_4][BF_4]$ in acetonitrile as electrolyte.

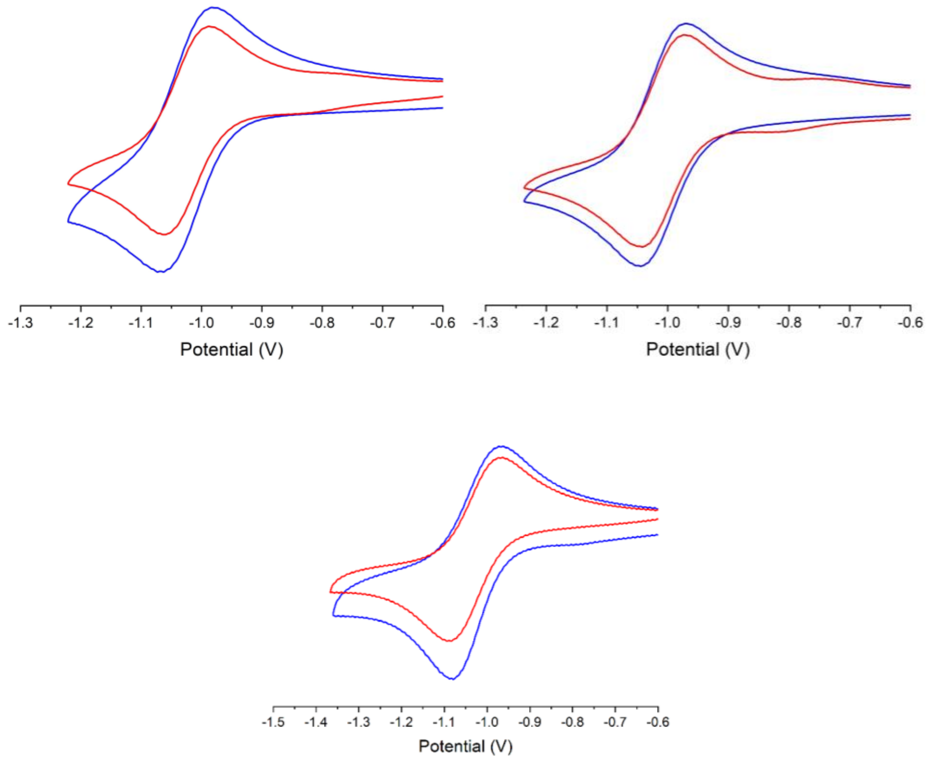

Figure S16 Cyclic voltammograms of $[NBu_4]_2$[**1**] (left), $[NBu_4]_2$[**2**] (right) and $[NBu_4]_2$[**3**] (bottom) before (blue) and after bulk electrolysis for 23 min at -0.7 V vs Ag/Ag^+. To aid comparison, the baseline current of the post-BE scans at the start potential have been shifted to the same value as the initial scan.

5. Spectroelectrochemistry and Electrochemically Switched HRS

Experimental details of spectroelectrochemistry experiments are provided in the main paper, some additional data is provided below:

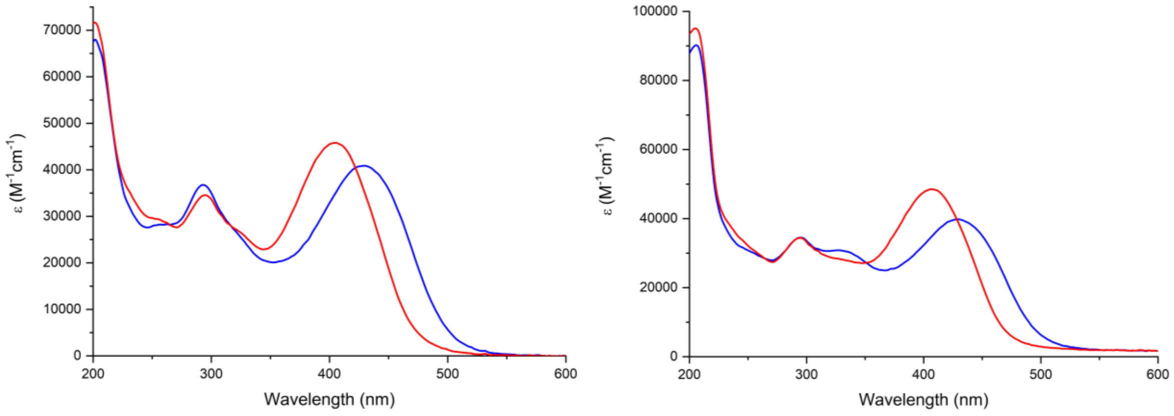

Figure S17: UV-vis spectra of $[NBu_4]_2$[**1**] (left) and $[NBu_4]_2$[**2**] (right) before (blue) and after (red) electrochemical reduction (concentration *ca*. 0.8 × 10^{-3} M in 0.3 M NBu_4BF_4). A recovery of 98% and 97% of the spectra of $[NBu_4]_2$[**1**] and $[NBu_4]_2$[**2**] were obtained upon reoxidation.

Electrochemically switched HRS measurements were performed on $[NBu_4]_2$[**3**] in a custom-designed two compartment cell with the working compartment constructed from a 1 cm path length Pyrex fluorescence cuvette as described previously.[14] Pt gauze was used as the working electrode, Ag wire (jacketed in a glass tube and fritted disk) as the reference, and a Pt wire coil as the counter. The analyte concentration was *ca* 10^{-4} M in 0.3 M $[NBu_4][BF_4]$: this high concentration was needed for electrochemical performance and to ensure an adequate difference between the sample and solvent/electrolyte baseline. HRS signal was recorded every 5 seconds as described above, with 10 measurements taken before applying potential to establish a baseline value for the oxidized states. A. To achieve switching a reductive electrochemical potential of -1.3 V vs Ag was applied using a Princeton Applied Research Parstat 2273 for 400 seconds before re-oxidation with a potential of 0 V vs Ag for a further 400 seconds. This cycle time is necessitated by the relatively large sample volume. Data are presented below in Figure S18.

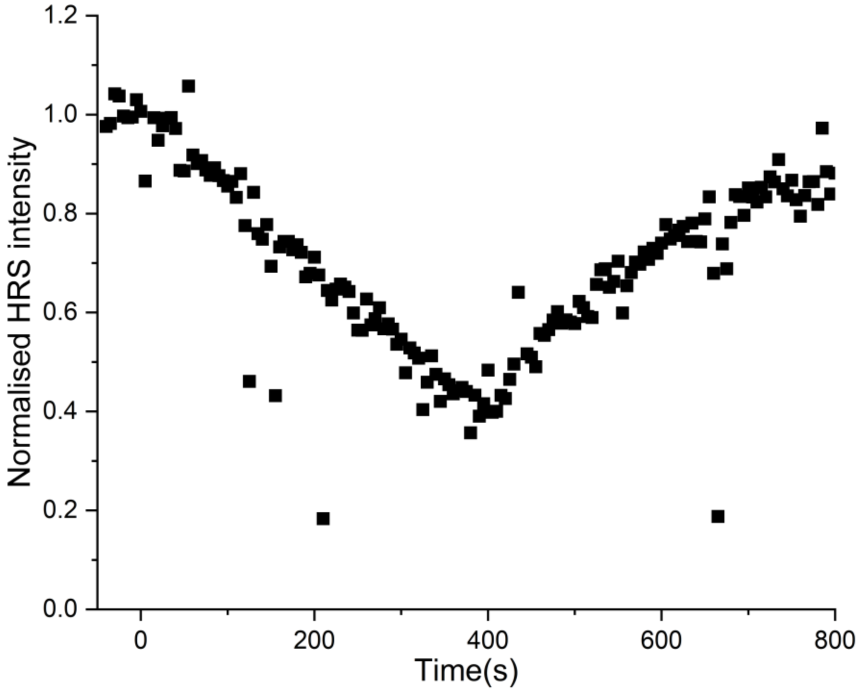

Figure S18: Response of the HRS signal intensity to reduction of $[NBu_4]_2[\mathbf{3}]$ (-1.3 V *vs* Ag wire) over first 400 seconds, and reoxidation (0 V *vs* Ag). Analyte concentration *ca.* 10^{-4} M in 0.3 M $[NBu_4][BF_4]$.

6. Quantum chemistry calculations

Geometry optimizations. Full geometry optimizations were performed at the density functional theory (DFT) level using the ωB97X-D exchange-correlation functional (XCF).[15] This range-separated hybrid functional combines a modified B97 exchange functional[16] for short-range interactions (from 78 to 0%) with HF exchange for long-range interactions (from 22 to 100%), using the default range-separating parameter, $\omega = 0.2$ Bohr^{-1}. Correlation is described by the B97 correlation functional,[16] and empirical atom-atom London dispersion corrections are included as well. For the C, H, N, and O atoms, the atomic basis set consists of 6-311G(d)[17] while LANL2TZ[18] is used for the Mo atoms. TIGHT convergence thresholds on the residual forces on the atoms (1.5×10^{-5} Hartree/Bohr or Hartree/radian) were applied. The reliability of the ωB97X-D/6-311G(d)/LANL2TZ method for the geometry optimization of POM derivatives was demonstrated in comparison with other XC functionals in a previous work.[19] To describe the solvent effects (acetonitrile), geometry optimizations were performed in solution using the integral equation formalism (IEF) of the polarizable continuum model (PCM) (IEF-PCM), which represents the solvent by a dielectric continuum characterized by its dielectric permittivity ($\varepsilon_0 = 35.688$ for acetonitrile).[20] For the reduced states $[\mathbf{X}]^{3-}$, unrestricted settings were used, for ground states values of the $\hat{S}^2$ operator were all $\cong 0.75$, indicating the absence of spin contamination.

Properties of the excited states. Using the optimized geometries, the time-dependent density functional theory (TD-DFT) method[21] with the same XCF, basis set, and IEF-PCM scheme was used to characterize the (lowest-energy) excited states, considering their vertical excitation

energies, $\Delta E_{ge} = E_e - E_g$ (from the ground state g to the excited state e), the corresponding transition dipole moment, μ_{ge}, which is related to the oscillator strength, $f_{ge} = \frac{2}{3}\,\Delta E_{ge}\,\mu_{ge}^2$, and the difference of dipole moment between states g and e, $\Delta\mu_{ge} = \mu_e - \mu_g$. The excited state dipole moments were calculated by using the nonequilibrium IEF-PCM solvation approach (ε_∞ = 1.807 for acetonitrile). Nonequilibrium solvation TDDFT calculations also provided the difference of electron density between the excited and the ground states, $\Delta\rho(\vec{r}) = \rho_e(\vec{r}) - \rho_g(\vec{r})$. Following Le Bahers *et al*,[22] the barycenters of the positive [$\Delta\rho^+(\vec{r})$] and negative [$\Delta\rho^-(\vec{r})$] electron density variations were calculated and the distance between them defines the charge-transfer distance (d_{CT}) while the integration over the whole space of $\Delta\rho^+(\vec{r})$ [or $\Delta\rho^-(\vec{r})$] gives the amount of charge transferred (q_{CT}). The product of these two quantities, $q_{CT}\,d_{CT}$, gives $\Delta\mu_{ge}$. The 30 lowest excitation energies (and oscillator strengths) were calculated. For the reduced species, unrestricted calculations were carried out. For the ground states values of the $\hat{S}^2$ operator were all ≅0.75, indicating the absence of spin contamination. Small spin contaminations were observed for several excited states, and values of $\hat{S}^2$are presented in Table S4.

First hyperpolarizabilities. Using again the same optimized geometries, the SHG β tensor components were evaluated by employing the quadratic response TD-DFT method[23,24] with the same basis set, XCF, and solvation model as for the excited states calculations. Both static and dynamic (incident wavelengths of 1064 nm and 1200 nm) responses were calculated. Computing β of large compounds, including those having donor and/or acceptor substituents, is a challenge for TD-DFT because of the intrinsic nonlocal nature of the response and the approximate XCFs (potential and kernel).[25] However, ωB97X-D is a reliable XCF for calculating the β tensors owing to its substantial amount of long-range HF exchange, as demonstrated in previous investigations where the performance of DFT XCFs was assessed with respect to benchmark wavefunction methods.[26-28] Ref. 19 has also evidenced that using local XCFs or XCFs with small amount of HF exchange leads to overestimated first hyperpolarizabilities. The unit sphere representation (USR) was adopted[29] to visualize the β tensors. First, the induced electric dipole moments

$$\vec{\mu}_{ind} = \overleftrightarrow{\beta} : \vec{E}^2(\theta, \phi) \qquad (1)$$

are evaluated, where $\overleftrightarrow{\beta}$ is the first hyperpolarizability tensor and $\vec{E}(\theta, \phi)$ is a unit vector of electric field, of which the polarization is defined by the θ and ϕ angles (spherical coordinates). Then, the induced dipoles are plotted on a sphere centered on the molecule center of mass. This allows highlighting the directions along which the second-order polarizations are the strongest (i.e. the largest induced dipoles), its orientation (the acceptor-donor direction).

In addition to considering the global β_{HRS} response and its depolarization ratio (ρ), the molecular response has been analyzed by assuming that only a few tensor components are non-zero. For linear, dipolar [**1**]$^{2-}$ to [**3**]$^{2-}$, a single tensor component β_{zzz} is assumed. For C_{2v} anion [**4**]$^{2-}$ input values are β_{HRS} and ρ while the output values are those tensor components in an effective/ideal Cartesian frame. Assuming Kleinman's conditions are satisfied for a C_{2v}

symmetry molecule, there are three independent tensor components, namely β_{zzz}, $\beta_{zyy} = \beta_{yzy} = \beta_{yyz}$, and $\beta_{zxx} = \beta_{xzx} = \beta_{xxz}$. Then, assuming planar symmetry, only two remain, β_{zzz} and $\beta_{zyy} = \beta_{yzy} = \beta_{yyz}$. Their relationships with the observable in VV and HV configurations are, where $\frac{\langle \beta_{ZZZ}^2 \rangle}{\langle \beta_{ZXX}^2 \rangle} = \rho$

$$\langle \beta_{ZZZ}^2 \rangle = \frac{1}{7}{\beta_{zzz}}^2 + \frac{6}{35}\beta_{zzz}\beta_{zyy} + \frac{9}{35}{\beta_{zyy}}^2 \qquad (2)$$

$$\langle \beta_{ZXX}^2 \rangle = \frac{1}{35}{\beta_{zzz}}^2 - \frac{2}{105}\beta_{zzz}\beta_{zyy} + \frac{11}{105}{\beta_{zyy}}^2 \qquad (3)$$

Starting from the macroscopic responses, the β_{zzz} and β_{zyy} quantities have been determined for [**4**]$^{2-}$. The solutions of the second order equations reported (in the main manuscript) are those that are more realistic given the nature of the molecules and by comparison with the USR, with the sign selected such that the main donor-POM axis points towards negative *z*, making β_{zzz} positive and β_{zyy} negative.

Computer codes. All DFT and TD-DFT calculations were performed using the Gaussian16 package.[30] The molecular structures, USR pictures, the electron density difference plots, and the simulated UV/vis absorption spectra were generated using the DrawMol software.[31]

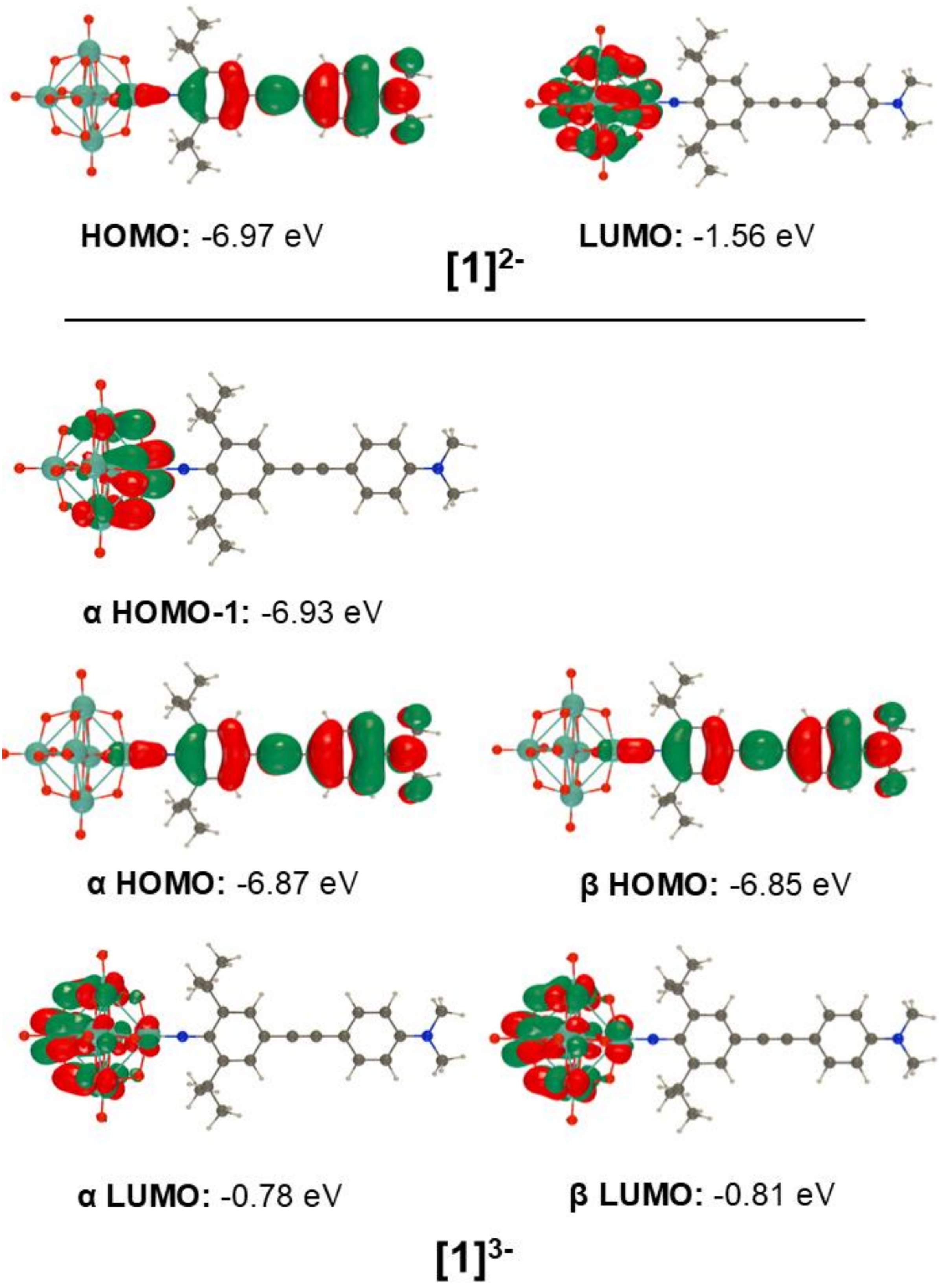

Figure S19: HOMO/HOMO-1 and LUMO orbitals of [**1**]$^{2-}$ (top) and reduced state [**1**]$^{3-}$ (bottom) calculated at the TDDFT/wB97X-D/6-311G(d)/LanL2TZ level of theory.

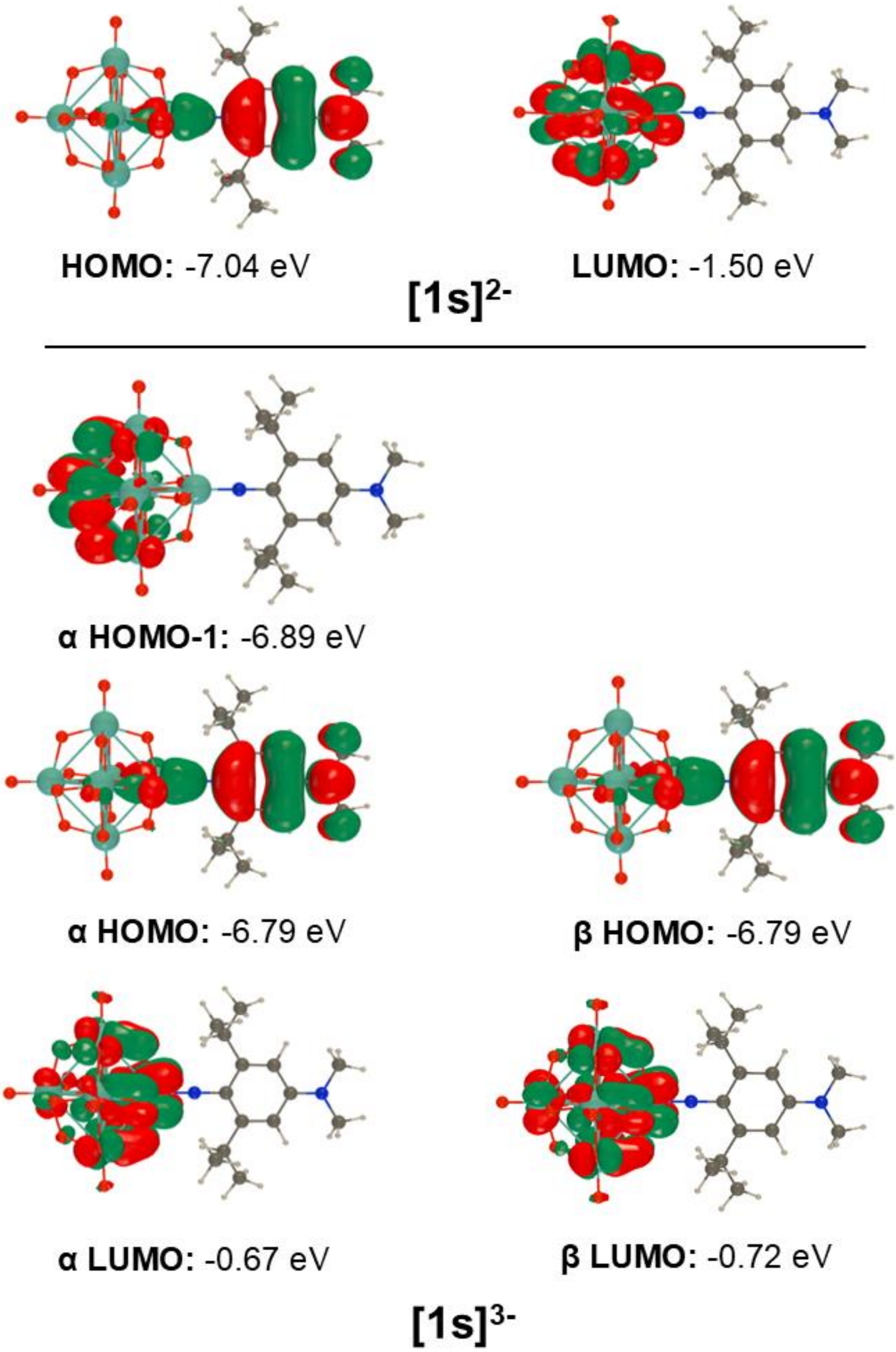

Figure S20: HOMO/HOMO-1 and LUMO orbitals of [**1s**]$^{2-}$ (top) and reduced state [**1s**]$^{3-}$ (bottom) calculated at the TDDFT/wB97X-D/6-311G(d)/LanL2TZ level of theory.

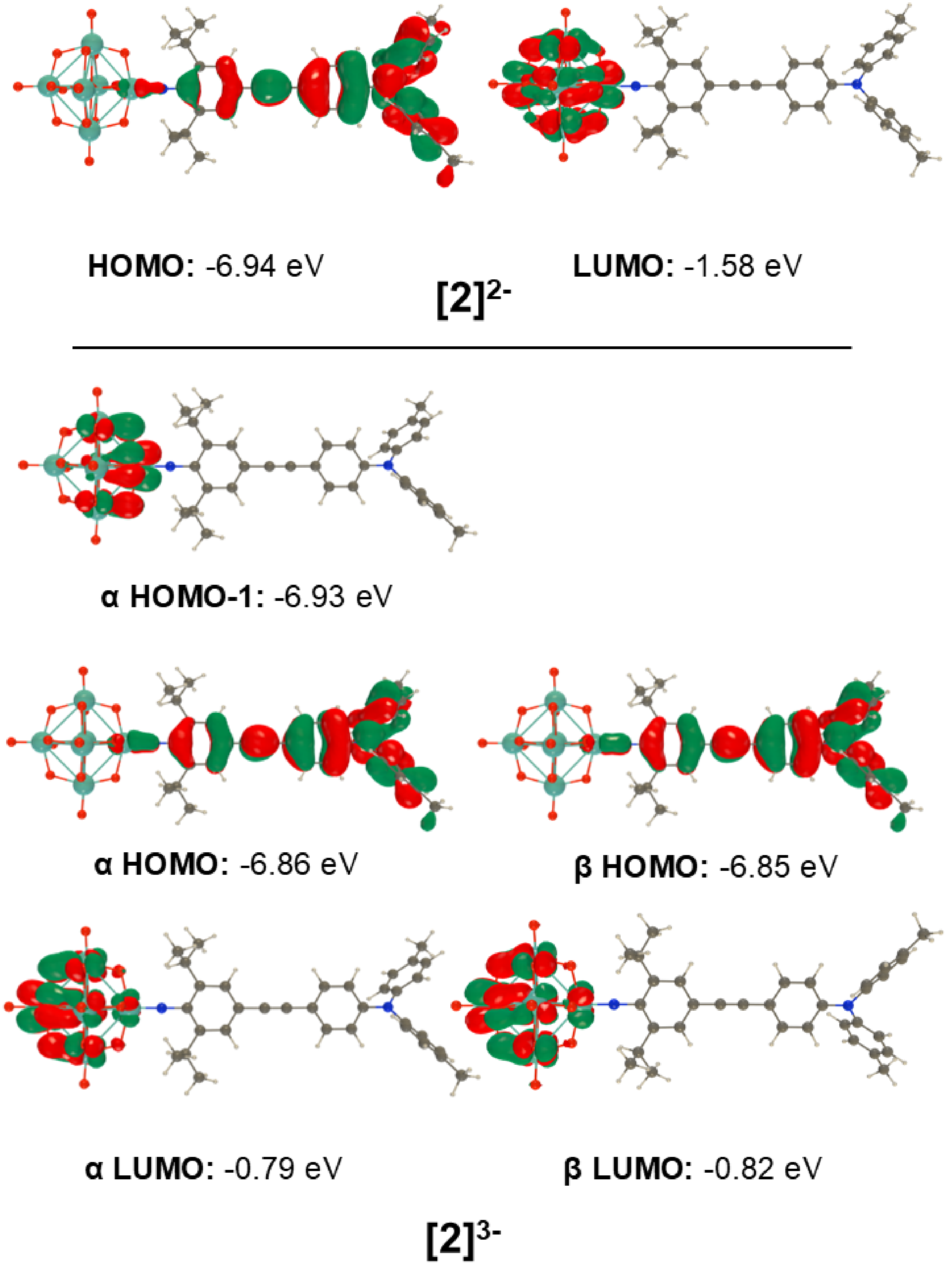

Figure S21: HOMO/HOMO-1 and LUMO orbitals of [**2**]$^{2-}$ (top) and reduced state [**2**]$^{3-}$ (bottom) calculated at the TDDFT/wB97X-D/6-311G(d)/LanL2TZ level of theory.

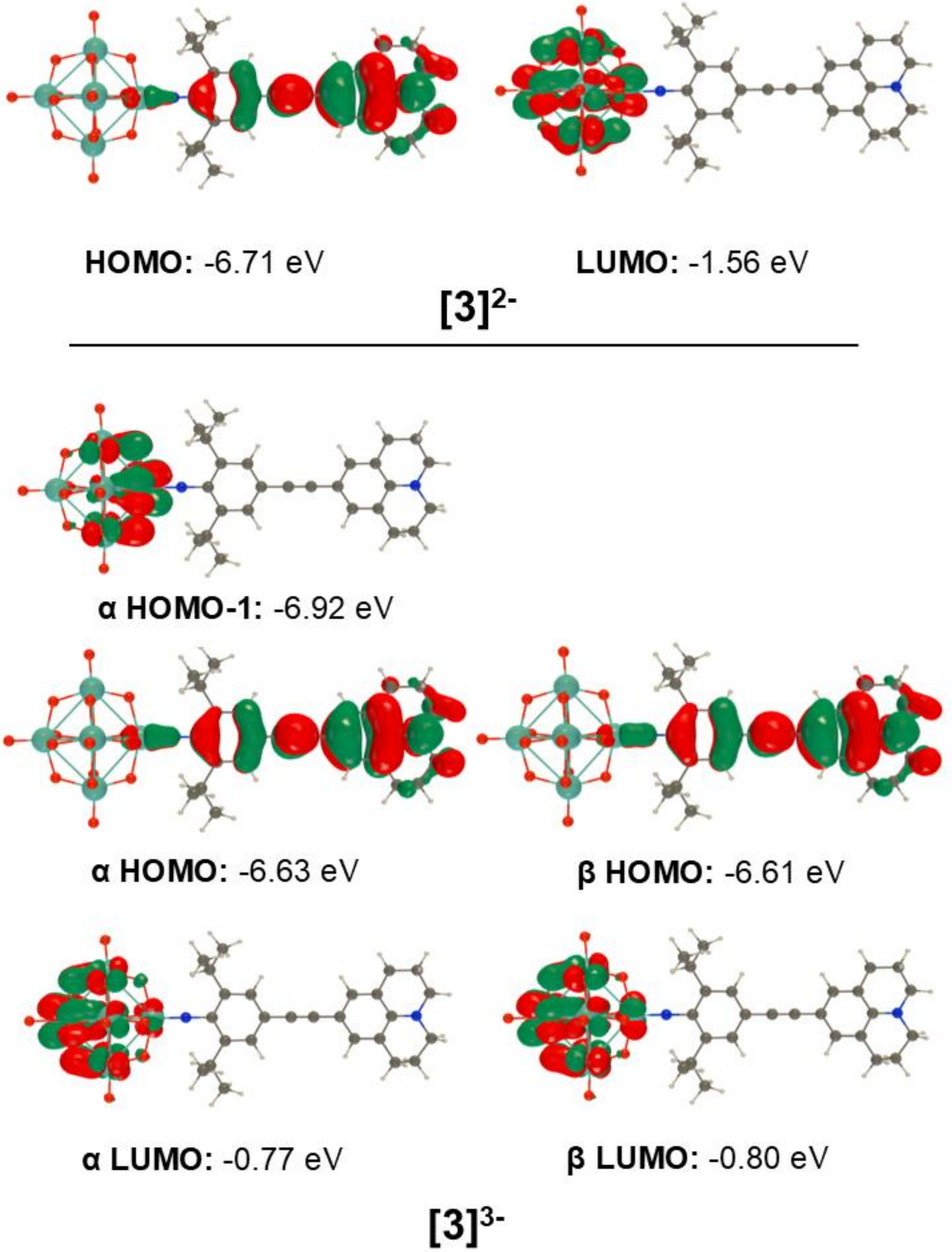

Figure S22: HOMO/HOMO-1 and LUMO orbitals of [**3**]$^{2-}$ (top) and reduced state [**3**]$^{3-}$ (bottom) calculated at the TDDFT/wB97X-D/6-311G(d)/LanL2TZ level of theory

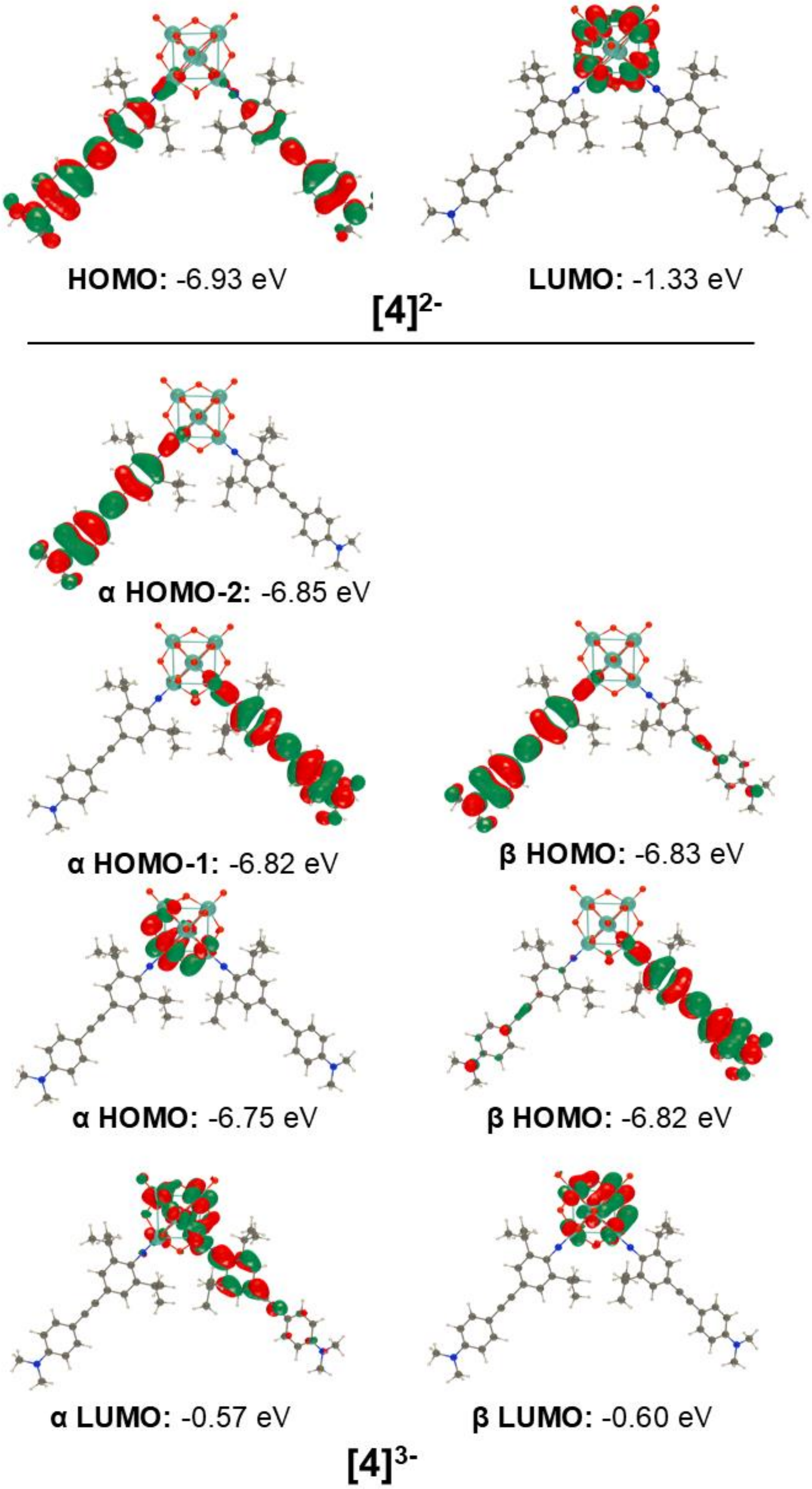

Figure S23: HOMO and LUMO orbitals of [**4**]$^{2-}$ (top) and reduced state [**4**]$^{3-}$ (bottom) calculated at the TDDFT/wB97X-D/6-311G(d)/LanL2TZ level of theory.

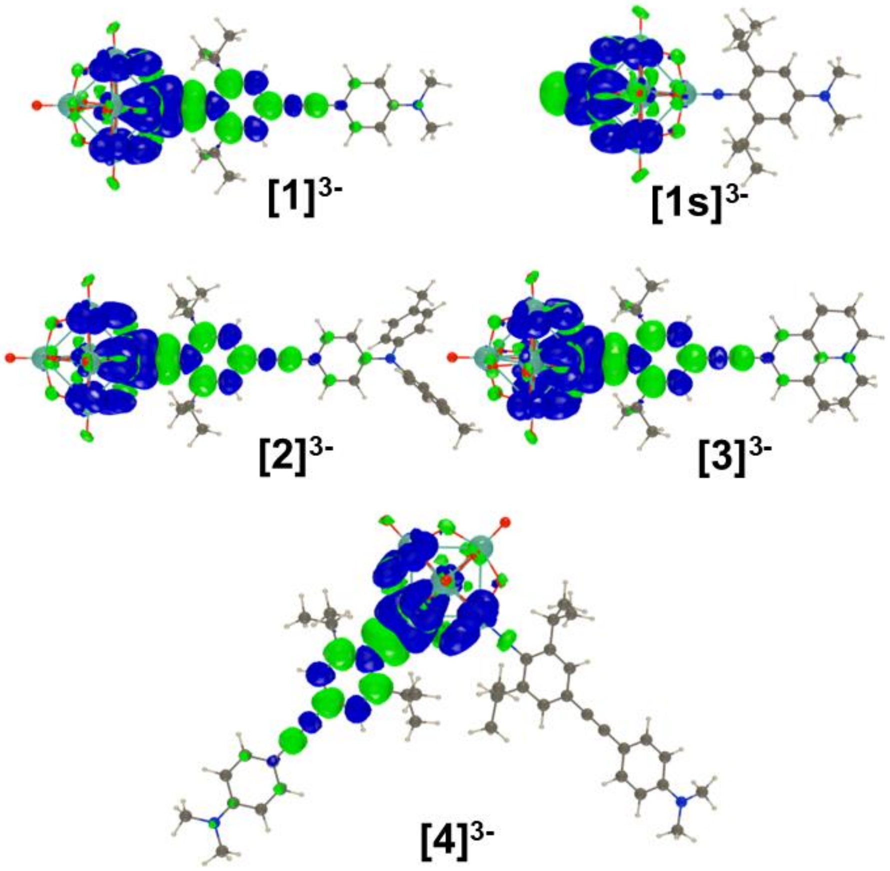

Figure S24: Spin densities of the reduced species as evaluated at the IEF-PCM(acetonitrile) /TDDFT/ωB97X-D/6-311G(d)/LanL2TZ level (isovalue = 0.0002 a.u.).

Table S3. Selected bond lengths (Å) and angles (°) of anions [**1**]$^{2-/3-}$, [**1s**]$^{2-/3-}$, [**2**]$^{2-/3-}$, [**3**]$^{2-/3-}$ and [**4**]$^{2-/3-}$. Mo^{im} – imido bearing Mo; O - central O atomin the POM. Alkyne means the angle between the two six-membered rings on each side of the alkyne.

	Mo^{im}-N^{im}	N^{im}-C	O-Mo^{im}-N^{im}	Mo^{im}-N^{im}-C	Alkyne twist angle
[**1**]$^{2-}$<br>[**1**]$^{3-}$	1.726<br>1.737	1.373<br>1.376	176.3<br>179.3	178.0<br>178.5	4.2<br>3.9
[**1s**]$^{2-}$<br>[**1s**]$^{3-}$	1.726<br>1.731	1.364<br>1.371	177.8<br>179.6	179.5<br>179.4	-<br>-
[**2**]$^{2-}$<br>[**2**]$^{3-}$	1.725<br>1.737	1.373<br>1.376	177.4<br>178.8	178.6<br>177.8	1.8<br>2.7
[**3**]$^{2-}$<br>[**3**]$^{3-}$	1.726<br>1.737	1.373<br>1.376	176.3<br>179.4	177.8<br>178.4	2.9<br>6.1
[**4**]$^{2-}$	1.728<br>1.728	1.374<br>1.374	175.0<br>174.9	175.9<br>175.6	1.0<br>0.3
[**4**]$^{3-}$	1.739<br>1.739	1.373<br>1.375	173.3<br>178.5	174.4<br>178.1	0.0<br>1.3

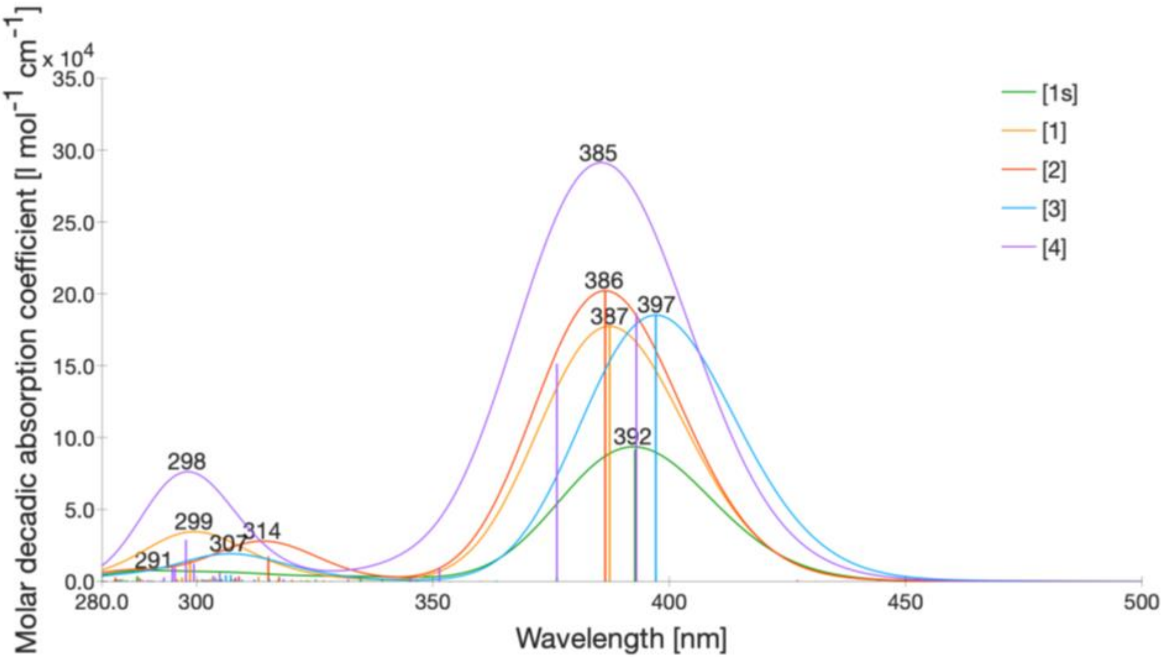

Figure S25: Computed absorption spectra of the oxidized forms [**1s**]$^{2-}$ and [**1**]$^{2-}$ to [**4**]$^{2-}$ as calculated at the IEFPCM (solvent = acetonitrile) TDDFT/wB97X-D/6-311G(d)/LanL2TZ level of approximation. FWHM = 0.3 eV. 30 excited states have been calculated.

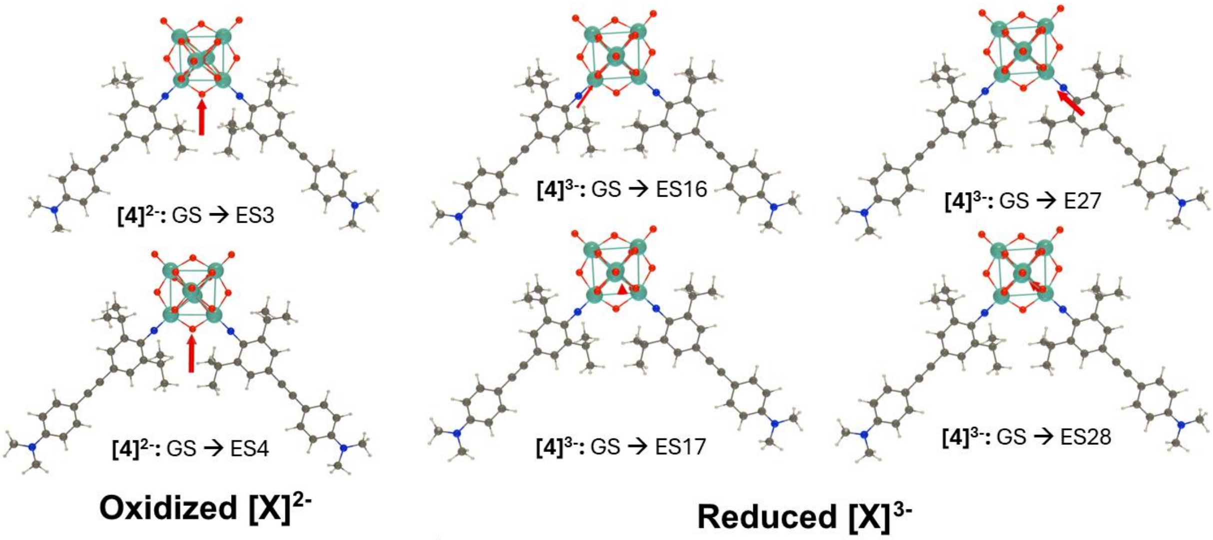

Figure S26. Charge transfer vectors (from negative to positive barycenter of $\Delta\rho$) upon excitation from GS to the nth ES of the oxidized and reduced states of the 2-D anion [**4**]$^{2-/3-}$ as evaluated at the IEF-PCM(acetonitrile)/TDDFT/ωB97X-D/6-311G(d)/LanL2TZ level. The nonequilibrium solvation approach was adopted.

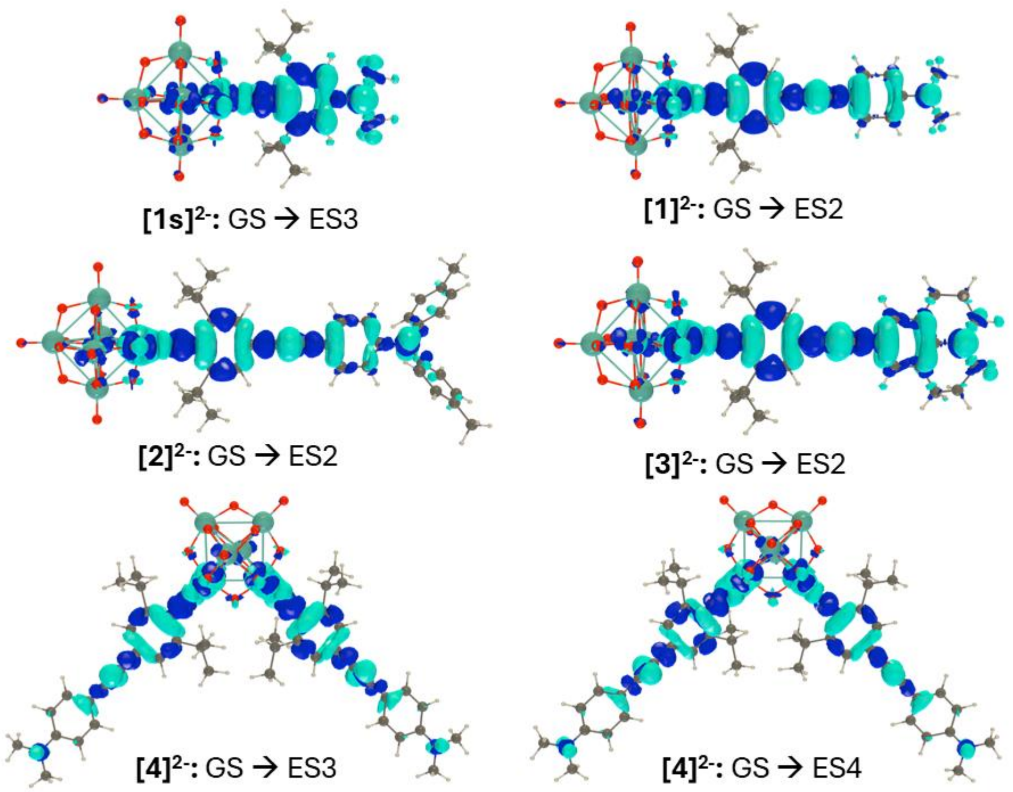

Figure S27. Variation of electron density (Δρ) upon excitation from the GS to the nth ES of the oxidized species [**1s**]$^{2-}$ and [**1**]$^{2-}$ to [**4**]$^{2-}$ as evaluated at the IEF-PCM(acetonitrile)/TDDFT/ωB97X-D/6-311G(d)/LanL2TZ level; light and dark blue correspond to negative and positive Δρ, respectively (isovalue = 0.0008 a.u.). The nonequilibrium solvation approach was adopted.

Table S4. Spin contaminations $\hat{S}^2$, computed amounts of charge transfer (q_{CT}), distances of charge transfer (d_{CT}), and variations of dipole moment upon excitation from the GS to the nth dominant lowest-energy excited state ($\Delta\mu_{GE}$) of the both oxidized [**X**]$^{2-}$ and reduced [**X**]$^{3-}$ species as calculated at the IEFPCM(acetonitrile) TDDFT/wB97X-D/6-311G(d)/LanL2TZ level of approximation. The nonequilibrium solvation approach was adopted.

	n	$\hat{S}^2$	q_{CT} (*e*)	d_{CT} (Å)	$\Delta\mu_{GE}$ (D)
[**1s**]$^{2-}$	3	-	0.63	3.17	9.52
[**1s**]$^{3-}$	14	0.76	0.60	2.79	8.10
[**1**]$^{2-}$	2	-	0.64	4.35	13.47
[**1**]$^{3-}$	20	0.96	0.52	3.26	8.06
[**2**]$^{2-}$	2	-	0.63	4.15	12.56
[**2**]$^{3-}$	21	0.94	0.51	2.89	7.10
[**3**]$^{2-}$	2	-	0.71	4.79	16.35
[**3**]$^{3-}$	20	1.00	0.55	3.72	9.89
[**4**]$^{2-}$	3	-	0.61	2.84	8.32
	4	-	0.59	2.93	8.30
[**4**]$^{3-}$	16	1.02	0.54	2.07	5.41
	17	1.08	0.63	0.82	2.48
	27	1.12	0.49	2.65	6.28
	28	1.60	0.71	1.38	4.71

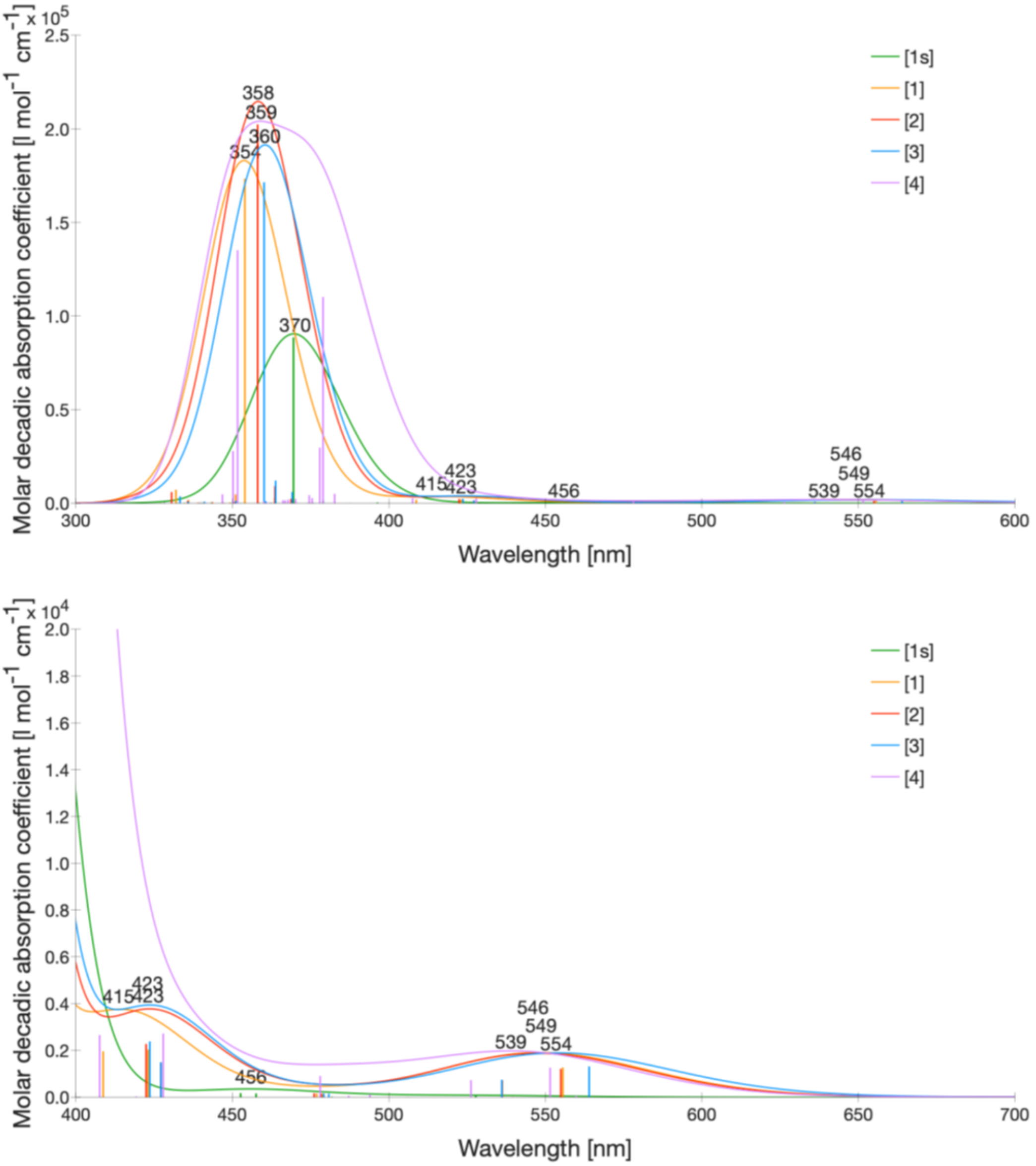

Figure S28. Computed absorption spectra of the reduced forms [**1s**]$^{3-}$ and [**1**]$^{3-}$ to [**4**]$^{3-}$ as calculated at the IEFPCM (solvent = acetonitrile) TDDFT/wB97X-D/6-311G(d)/LanL2TZ level of approximation. FWHM = 0.3 eV. 30 excited states have been calculated.

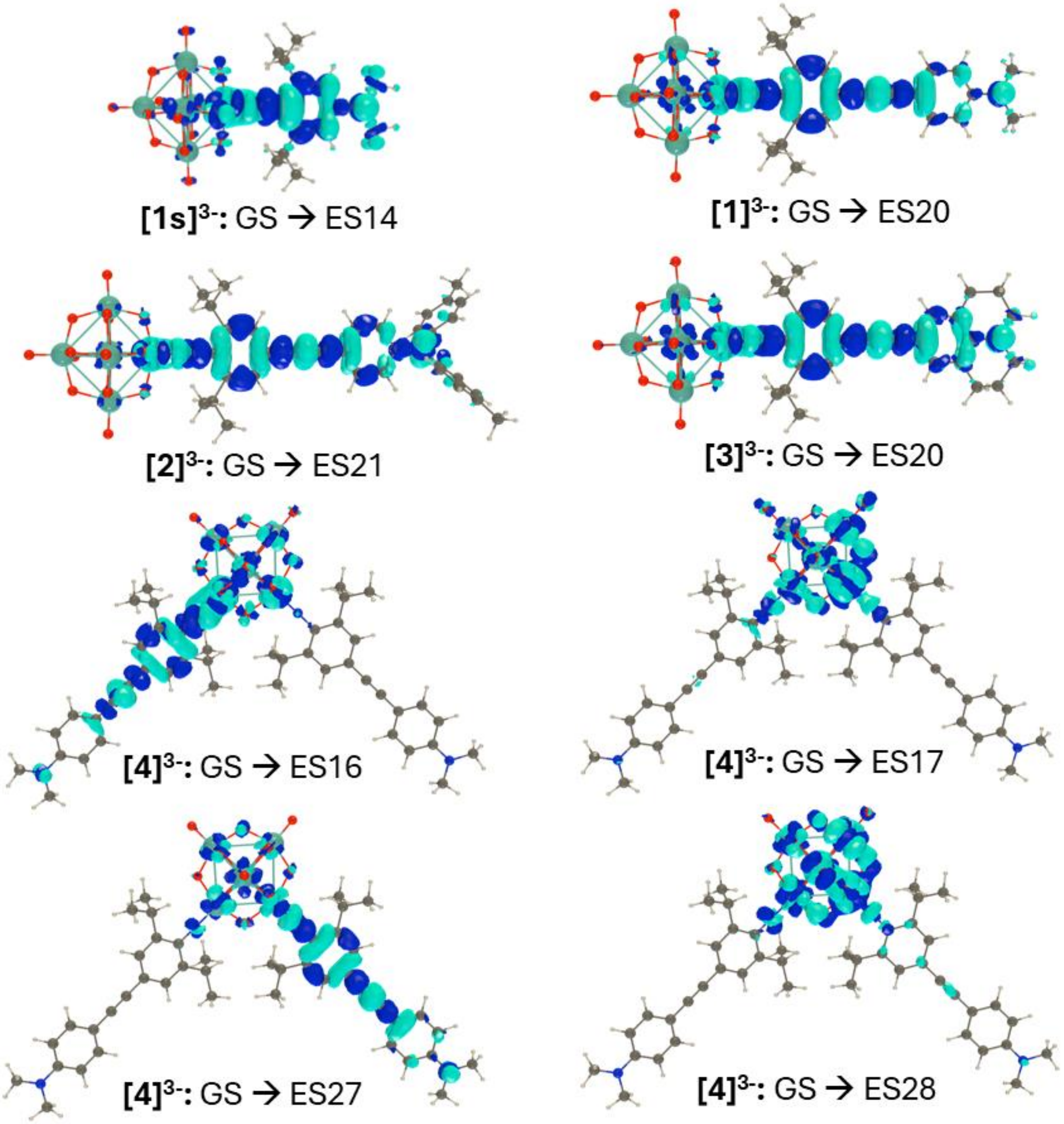

Figure S29. Variation of electron density (Δρ) upon excitation from the GS to the nth ES of the reduced species [**1s**]$^{3-}$ and [**1**]$^{3-}$ to [**4**]$^{3-}$ as evaluated at the IEF-PCM(acetonitrile)/TDDFT/ωB97X-D/6-311G(d)/LanL2TZ level; light and dark blue correspond to negative and positive Δρ, respectively (isovalue = 0.0008 a.u.). The nonequilibrium solvation approach was adopted.

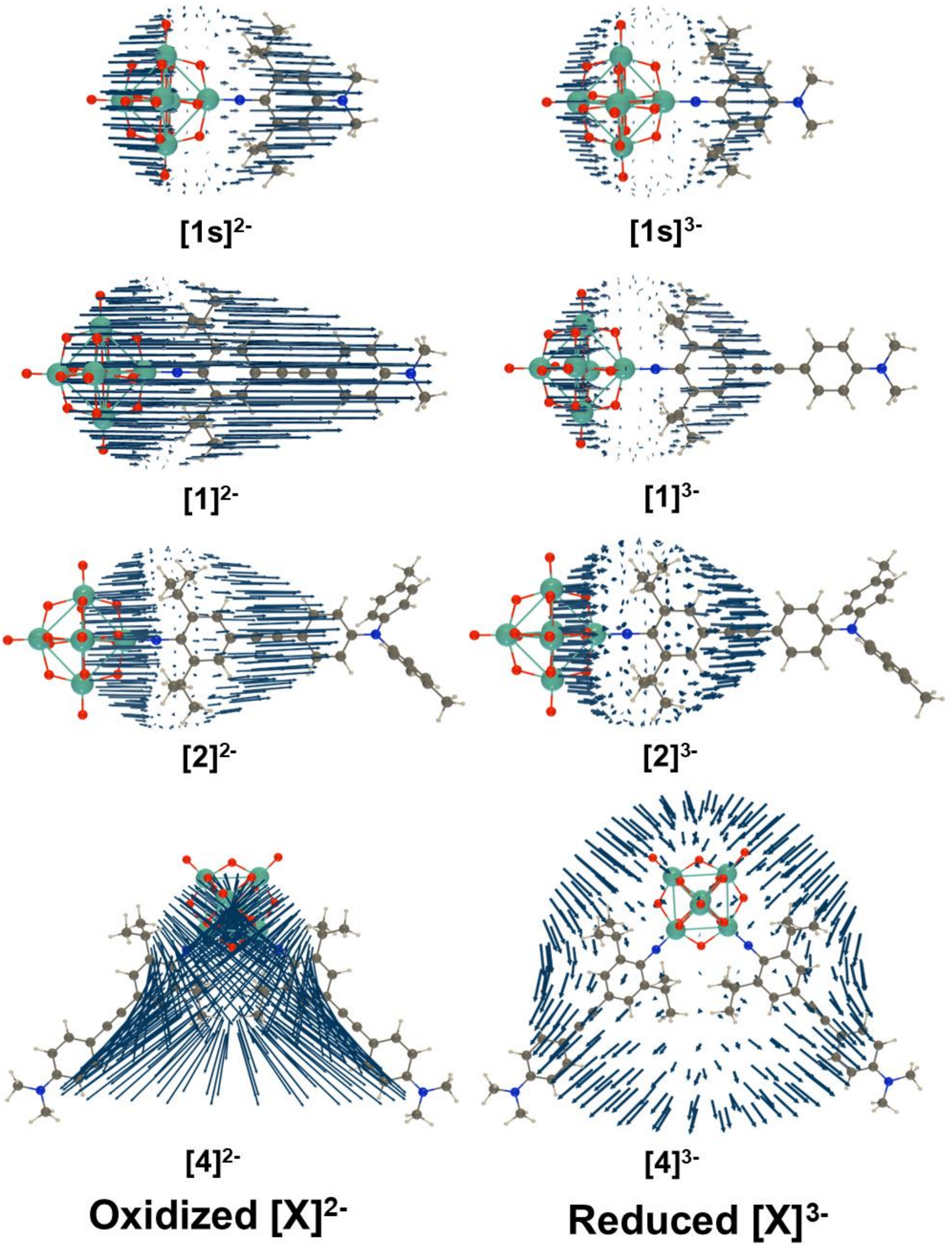

Figure S30. Unit sphere representation (USR) of the first hyperpolarizability tensor (λ = 1064 nm) of oxidized [**X**]$^{2-}$ and reduced [**X**]$^{3-}$ (**X** = **1s, 1, 2, 4**) anions as calculated at the IEFPCM(solvent = acetonitrile) TDDFT/wB97X-D/6-311G(d)/LanL2TZ level of approximation. Factor = 10^{-4} Å/a.u.$_{\beta}$.

References

1. D'Souza, B. R.; Lane, T. K.; Louie, J. Iron-Catalyzed Cycloaddition of Alkynenitriles and Alkynes. *Org. Let.* **2011**, *13*, 2936-2939

2. Man, W. Y.; Vincent, K. B.; Spencer, H. J.; Yufit, D. S.; Howardm J. A. K.; Low, P. J. Synthesis, Structure and Electrochemical Properties of Triarylamine Bridged Dicobaltdicarbon Tetrahedrane Clusters, *J. Clust. Sci.* **2012**, *23*, 853-873.

3. Yamada, S.; Morita, M.; Konno, T. Multi-color photoluminescence induced by electron-density distribution of fluorinated bistolane derivatives. *J. Fluor. Chem.* **2017**, *202*, 54-64.

4. Breiten, B.; Wu, Y.; Jarowski, P. D.; Gisselbrecht, J.-P.; Boudon, C.; Griesser, M.; Onitsch, C.; Gescheidt, G.; Schweizer, W. B.; Langer, N.; Lennartz, C.; Diederich, F. Donor-substituted octacyano[4]dendralenes: a new class of cyano-rich non-planar organic acceptors, *Chem. Sci*. **2011**, *2*, 88-93.

5. Lu, M.; Kang, J.; Wang, D.; Peng, Z. Enantiopure 1,1'-Binaphthyl-Based Polyoxometalate-Containing Molecular Hybrids. *Inorg. Chem.* **2005**, *44*, 7711-7713.

6. Xu, L.; Lu, M.; Xu, B.; Wei, Y.; Peng, Z.; Powell, D. R. Towards Main-Chain-Polyoxometalate-Containing Hybrid Polymers: A Highly Efficient Approach to Bifunctionalized Organoimido Derivatives of Hexamolybdates. *Angew. Chem. Int. Ed.* **2002**, *41*, 4129-4132.

7. Bar-Nahum, I.; Narasimhulu, K. V. ; Weiner, L.; Neumann, R. Phenanthroline −Polyoxometalate Hybrid Compounds and the Observation of Intramolecular Charge Transfer. *Inorg. Chem*. **2005**, *44*, 4900−4902.

8. (a) Al-Yasari, A.; Van Steerteghem, N.; El Moll, H.; Clays, K.; Fielden, J. Donor–Acceptor Organo-Imido Polyoxometalates: High Transparency, High Activity Redox-Active NLO Chromophores. *Dalton Trans*. **2016**, *45*, 2818-2822. (b) Al-Yasari, A.; Van Steerteghem, N.; Kearns, H.; El Moll, H.; Faulds, K.; Wright, J. A.; Brunschwig, B. S.; Clays, K.; Fielden, J. Organoimido-Polyoxometalate Nonlinear Optical Chromophores: A Structural, Spectroscopic, and Computational Study. *Inorg. Chem*. **2017**, *17*, 10181-10194. (c) Al-Yasari, A.; Spence, P.; El Moll, H.; Van Steerteghem, N.; Horton, P. N.; Brunschwig, B. S.; Clays, K.; Fielden, J. Fine-tuning Polyoxometalate Non-Linear Optical Chromophores: a Molecular Electronic "Goldilocks" Effect. *Dalton Trans*. **2018**, *47*, 10415-10419. (d) Jones, C. F.; Hood, B. R.; de Coene, Y.; Lopez-Poves, I.; Champagne, B.; Clays, K.; Fielden, J. Bridge Improvement Work: Maximising Non-Linear Optical Performance in Polyoxometalate Derivatives. *Chem. Commun.* **2024**, *60*, 1731-1734. (e) Al-Yasari, A.; El Moll, H.; Purdy, R.; Vincent, K. B.; Spence, P.; Malval, J.-P.; Fielden, J. Optical, third order non-linear optical and electrochemical properties of dipolar, centrosymmetric and C_{2v} organoimido polyoxometalate derivatives. *Phys. Chem. Chem. Phys.* **2021**, *23*, 11807-11817.

9. *CrysAlisPro* (Version 1.171.40.68a), Rigaku Oxford Diffraction, Rigaku Corporation, Tokyo, Japan, **2019**.

10. G. M. Sheldrick, *Acta Cryst. A*, **2015**, *71*, 3.

11. O. V. Dolomanov, L. J. Bourhis, R. J. Gildea, J. A. K. Howard, H. Puschmann, *J. Appl. Crysallogr*. **2009**, *42*, 339.

12. G. M. Sheldrick, *Acta. Cryst. C*, **2015**, *71*, 3.

13. (a) Strong, J. B.; Yap, G. P. A.: Ostrander, R.; Liable-Sands, L. M.; Rheingold, A. L.; Thouvenot, R.; Gouzerh, P.; Maatta. E. A. A New Class of Functionalized Polyoxometalates: Synthetic, Structural, Spectroscopic, and Electrochemical Studies of Organoimido Derivatives of $[Mo_6O_{19}]^{2-}$. *J. Am. Chem. Soc.* **2000**, *122*, 639-649. (b) Xu, B.; Wei, Y.; Barnes, C. L.; Peng, Z. Hybrid Molecular Materials Based on Covalently Linked Inorganic Polyoxometalates and Organic Conjugated Systems. *Angew. Chem. Int. Ed.* **2001**, *40*, 2290-2292.

14. Hood, B. R.; de Coene, Y.; Torre Do Vale Froes, A. V.; Jones, C. F.; Beaujean, P.; Liégois, V.; MacMillan, F.; Champagne, B.; Clays, K.; Fielden, J. Electrochemically-Switched 2nd Order Non-Linear Optical Response in an Arylimido-Polyoxometalate with High Contrast and Cyclability. *Angew. Chem. Int. Ed.* **2023**, *62*, e202215537.

15. Chai, J. D.; Head-Gordon, M. Long-Range Corrected Hybrid Density Functionals with Damped Atom-Atom Dispersion Corrections. *Phys. Chem. Chem. Phys.* **2008**, *10*, 6615-6620.

16. Becke, A. D. Density-Functional Thermochemistry. V. Systematic Optimization of Exchange Correlation Functionals. *J. Chem. Phys*. **1997**, *107*, 8554-8560

17. Krishnan, R.; Binkley, J. S.; Seeger, R.; Pople, J. A. Self-consistent Molecular Orbital Methods. XX. A Basis Set for Correlated Wavefunctions. *J. Chem. Phys.* **1980**, *72*, 650-654.

18. Roy, L.E.; Hay, P. J.; Martin, R. L. Revised Basis Sets for the LANL Effective Core Potentials. *J. Chem. Theory Comput.* **2008**, *4*, 1029-1031

19. Rtibi, E.; Abderrabba, M.; Ayadi, S.; Champagne, B. *Inorg. Chem*. **2019**, *58*, 11210-11219.

20. Tomasi, J.; Mennucci, B.; Cammi, R. Quantum Mechanical Continuum Solvation Models. *Chem. Rev*. **2005**, *105*, 2999-3094.

21. Casida, M. E.; pp 155-192 in *Recent advances in Density Functional Theory*, ed. Chong, D. P.; World Scientific: Singapore, **1995**.

22. Le Bahers, T.; Adamo, C.; Ciofini, I. A Qualitative Index of Spatial Extent in Charge-Transfer Excitations. *J. Chem. Theory Comput.* **2011**, *7*, 2498-2506.

23. Van Gisbergen, S. J. A.; Snijders, J. G.; Baerends, E. J. Calculating Frequency-Dependent Hyperpolarizabilities using Time-Dependent Density Functional Theory. *J. Chem. Phys*. **1998**, *109*, 10644-10656.

24. Helgaker, T.; Coriani, S.; Jørgensen, P.; Kristensen, K.; Olsen, J.; Ruud, K. Recent Advances in Wave Function-Based Methods of Molecular Property Calculautions. *Chem. Rev.* **2012**, *112*, 543-631.

25. Champagne, B.; Beaujean, P.; de Wergifosse, M.; Cardenuto, M.; Liégeois, V.; Castet, F. pp 117-138 in *Frontiers of Quantum Chemistry*, Eds: Wojcik, M.; Nakatsuji, H.; Kirtman, B.; Ozaki, Y.; Springer: Singapore, **2018**.

26. de Wergifosse, M.; Champagne, B. Electron Correlation Effects on the First Hyperpolarizability of Push-Pull π-Conjugated Systems. *J. Chem. Phys.* **2011**, *134*, 074113.

27. Johnson, L. E.; Dalton, L. R.; Robinson, B. H. Optimising Calculations of Electronic Excitations and Relative Hyperpolarizabilities of Electrooptic Chromophores. *Acc. Chem. Res*. **2014**, *47*, 3258-3265.

28. Garrett, K.; Sosa Vazquez, X. A.; Egri, S. B.; Wilmer, J.; Johnson, L. E.; Robinson, B. H.; Isborn, C. M. Optimum Exchange for Calculation of Excitation Energies and Hyperpolarizabilities of Organic Electro-optic Chromophores, *J. Chem. Theory Comput.* **2014**, *10*, 3821-3831.

29. Tuer, A.; Krouglov, S.; Cisek, R.; Tokarz, D.; Barzda, V. Three-Dimensional Visualization of the First Hyperpolarizability Tensor. *J. Comput. Chem.* **2011**, *32*, 1128-1134.

30. Frisch, M. J.; Trucks, G. W.; Schlegel, H. B.; Scuseria, G. E.; Robb, M. A.; Cheeseman, J. R.; Scalmani, G.; Barone, V.; Petersson, G. A.; Nakatsuji, H.; Li, X.; Caricato, M.; Marenich, A. V.; Bloino, J.; Janesko, B. G.; Gomperts, R.; Mennucci, B.; Hratchian, H. P.; Ortiz, J. V.; Izmaylov, A. F.; Sonnenberg, J. L.; Williams-Young, D.; Ding, F.; Lipparini, F.; Egidi, F.; Goings, J.; Peng, B.; Petrone, A.; Henderson, T.; Ranasinghe, D.; Zakrzewski, V. G.; Gao, J.; Rega, N.; Zheng, G.; Liang, W.; Hada, M.; Ehara, M.; Toyota, K.; Fukuda, R.; Hasegawa, J.; Ishida, M.; Nakajima, T.; Honda, Y.; Kitao, O.; Nakai, H.; Vreven, T.; Throssell, K.; Montgomery Jr., J. A.; Peralta, J. E.; Ogliaro, F.; Bearpark, M. J.; Heyd, J. J.; Brothers, E. N.; Kudin, K. N.; Staroverov, V. N.; Keith, T. A.; Kobayashi, R.; Normand, J; Raghavachari, K.; Rendell, A. P.; Burant, J. C.; Iyengar, S. S.; Tomasi, J.; Cossi, M.; Millam, J. M.; Klene, M.; Adamo, C.; Cammi, R.; Ochterski, J. W.; Martin, R. L.; Morokuma, K.; Farkas, O.; Foresman, J. B.; Fox, D. J. Gaussian16 Revision A.03, **2016**.

31. DrawMol, Liégeois, V. U. Namur, www.unamur.be/drawmol.
